# Data and non-linear models for the estimation of biomass growth and carbon fixation in managed forests

**DOI:** 10.1016/j.dib.2019.103841

**Published:** 2019-03-16

**Authors:** Ariane Albers, Pierre Collet, Anthony Benoist, Arnaud Hélias

**Affiliations:** a IFP Energies Nouvelles, 1 et 4 Avenue de Bois-Préau, 92852 Rueil-Malmaison, France; b LBE, Montpellier SupAgro, INRA, UNIV Montpellier, Narbonne, France; c Elsa, Research Group for Environmental Lifecycle and Sustainability Assessment, Montpellier, France; d CIRAD – UPR BioWooEB, Avenue Agropolis, F-34398, Montpellier, France; e Chair of Sustainable Engineering, Technische Universität Berlin, Berlin, Germany

**Keywords:** Biogenic carbon modelling, Carbon fixation, Forestry biomass, Non-linear growth

## Abstract

The data and analyses presented support the research article entitled “Coupling partial-equilibrium and dynamic biogenic carbon models to assess future transport scenarios in France” (Albers et al., 2019). Carbon sequestration and storage in forestry products (e.g. transport fuels) is sought as a climate change mitigation option. The data presented support and inform dynamic modelling approaches to predict biomass growth and carbon fixation dynamics, of a tree or forest stand, over specific rotation lengths. Data consists of species-specific yield tables, parameters for non-linear growth models and allometric equations. Non-linear growth models and allometric equations are listed and described. National statistics and surveys of the wood supply chain serve to identify main tree species, standing wood volumes and distributions within specific geographies; here corresponding to managed forests in France. All necessary data and methods for the computation of the annual fixation flows are presented.

**Table:** Specifications table

Subject area	Biology, Ecological modelling
More specific subject area	Dynamic modelling of forest biomass growth and annual carbon fixation
Type of data	Text, figures, tables
How data was acquired	Combination of secondary sources from public datasets available online and peer-reviewed literature, including national statistics and surveys, yield tables, non-linear growth parameters and allometric relations.
Data format	Filtered and analysed secondary data.
Experimental factors	Some data was re-expressed into different units when necessary to inform the models.
Experimental features	Non-linear growth was computed using data retrieved from yield tables. Initial parameters were compiled from literature to fit the non-self-starting non-linear regression model used for growth. Allometric equations were compiled and selected for tree volume estimations. Finally, mean biomass growth and carbon fixation was computed per one tonne of forestry biomass of interest.
Data source location	Managed forest systems in France or from other regions when data was not available for France (see Table 1).
Data accessibility	All data used and generated is included in this article and in its Supplementary Material
Related research article	A. Albers, P. Collet, D. Lorne, A. Benoist, A. Hélias, Coupling partial-equilibrium and dynamic biogenic carbon models to assess future transport scenarios in France, Appl. Energy. 239 (2019) 316–330. https://doi.org/10.1016/j.apenergy.2019.01.186

**Table:** 

**Value of the data** • A large compilation of secondary data, useful to facilitate dynamic carbon modelling of biomass growth and carbon fixation in managed forest systems. • Part of the data is generic enough to be used to model stands of unknown or mixed species. • The proposed modelling approach is flexible and applicable to any tree species and management practice (R script to fit non-self-starting non-linear regression growth parameters included). • Annual carbon stocking factors are provided for all tree species of the French wood supply chain.

## 1 Data

The data presented provides the basis for a non-linear forestry biomass growth model, whose outputs were used for modelling time-dependent carbon fixation in forest biomass [1]. This data article aggregates data from various datasets, including national statistics and surveys, yield tables, non-linear growth parameters and allometric relations (Table 1). The wood supply chain in France is represented by 12 main forest tree species (Table 2). National surveys and statistical results describe the distribution per tree species, used for weighted mean estimates (Table 3). Yield tables tabulate the age-dependent mean tree development and productivity of fully stocked managed stands, measured largely from long-standing experimental forest stand surveys. Yield table data is used to estimate i) initial parameters to fit non-self-starting non-linear regression models to predict tree growth, ii) age-dependent growth variables, and iii) site-dependent management practices (e.g. thinning periods, rotation cycles). Allometric models are used for volume estimation. All data sources primary originate from French studies, for geographical coherence. However, adequate European studies were retained when French data was unavailable (Table 4). Biomass yield and carbon content were obtained by applying specific conversion factors (Table 5). The *Supplementary Material* provides technical guidance and data for all assessed tree species concerning selected yield tables, regression analysis and parameters, biomass yield calculations, and annual carbon stocking factors. It includes a R [2] script to compute the regression parameters for running the growth model, applicable to future studies.

**Table 1:** General sources.

Specific data	Databases	Source
Species traits	Global TRY Plant Trait Database (https://www.try-db.org/TryWeb/Home.php)	[3]
National forestry inventories	National Institute of Geographic and Forest Information, Ministry of Agriculture, Agro-food and Forests	[4]
Wood density	International DRYAD Global Wood Density Database (http://datadryad.org/)	[5]
Allometric equations	GlobAllomeTree international database platform (http://www.globallometree.org/about/)	[6]
Carbon content	Food and Agricultural institute (FAO), Forestry Commission, and other	[7], [8]

**Table 2:** Species traits of forest species wood supply chain in France.

Common name	Species botanical name	Family	Genus	Species epithet	Leaf type	Leaf Phenology
Douglas fir	*Pseudotsuga menziesii*	Pinaceae	Pseudotsuga	menziesii	needle	evergreen
Norway spruce	*Piceaabies*	Pinaceae	Picea	abies	needle	evergreen
Maritime pine	*Pinus pinaster*	Pinaceae	Pinus	pinaster	needle	evergreen
Silver fir	*Abies alba*	Pinaceae	Abies	Alba	needle	evergreen
Scots pine	*Pinus sylvestri*	Pinaceae	Pinus	sylvestri	needle	evergreen
Sweet chestnut	*Castanea sativa*	Fagacea	Castanea	sativa	broadleaf	deciduous
Hornbeam	*Carpinus betulus*	Corylaceae	Carpinus	betulus	broadleaf	deciduous
Ash	*Fraxinus excelsior*	Oleaceae	Fraxinus	excelsior	broadleaf	deciduous
European beech	*Fagus sylvatica*	Fagacea	Fagus	sylvatica	broadleaf	deciduous
Sessile oak	*Quercus petraea*	Fagacea	Quercus	petraea	broadleaf	deciduous
English oak	*Quercus robur*	Fagacea	Quercus	robur	broadleaf	deciduous
White oak	*Quercus pubescens*	Fagacea	Quercus	pubescens	broadleaf	deciduous

Source: Global TRY Plant Trait Database [3].

**Table 3:** National inventory (2012–2016) and distribution of living standing volume per forest tree species in France.

Common name	Species	Distribution standing volume [Bm^3^]	Distribution standing volume [%]
Douglas fir	*P. menziesii*	106	4
Norway spruce	*P. abies*	213	8
Maritime pine	*P. pinaster*	133	5
Silver fir	*A. alba*	213	8
Scots pine	*P. sylvestri*	160	6
Other conifers	*Pinaceae* spp	146	6
Sweet chestnut	*C. sativa*	135	5
Hornbeam	*C. betulus*	108	4
Ash	*F. excelsior*	108	4
European beech	*F. sylvatica*	297	11
Sessile oak	*Q. petraea*	297	11
English oak	*Q. robur*	297	11
White oak	*Q. pubescens*	108	4
Other broadleaved	*Fagacea* spp	365	14

Source: [4].

**Table 4:** Specifications on analysed yield tables per forest tree species.

Common name	Species	Country	Eco-region	Geographical specifications	Yield class	Source	Page in source document
Douglas fir	*P. menziesii*	France	West Massif Central	Creuse, Corrèze et Haute-Vienne	2	[9]	50
Norway spruce	*P. abies*	France	South Massif Central	Montagne Noire, Monts de Lacune-Sommail-Espinouse, Levezou and Aigoual	16	[9]	134
Maritime pine	*P. pinaster*	France	South-West	Landes de Gascogne	3	[9]	54
Silver fir	*A. alba*	France	Jura	N/A	12	[9]	112
Scots pine	*P. sylvestri*	France	Sologne	N/A	3	[9]	20
Other conifers	*C. sativa*	Spain	North Spain	N/A	4	[10]	131
Sweet chestnut	*C. betulus*	N/A	European part	Eco-regions of deciduous forests and forest steppe	2	[11]	375
Hornbeam	*F. excelsior*	N/A	Northern Eurasia	N/A	2	[11]	108
Ash	*F. sylvatica*	France	North-West	N/A	6	[9]	84
European beech	*Q. petraea*	France	Loire	N/A		[9]	
Sessile oak	*Q. robur*	N/A	European part	Eco-regions of mixed forests, deciduous forests and forest steppe	1a	[11]	294
English oak	*Q. pubescens*	N/A	European part	Eco-regions of mixed forests, deciduous forests and forest steppe	2	[11]	295

**Table 5:** Wood density and carbon content per forest tree species.

Common name	Species	Wood density [t·m^−3^]	Carbon content [C·t^−1^]
Douglas fir	*P. menziesii*	0.4533	0.5280
Norway spruce	*P. abies*	0.3700	0.4980
Maritime pine	*P. pinaster*	0.4140	0.5212
Silver fir	*A. alba*	0.3530	0.4750
Scots pine	*P. sylvestri*	0.4219	0.5036
Other conifers	*Pinaceae* spp	0.4024	0.5052
Sweet chestnut	*C. sativa*	0.4400	0.5010
Hornbeam	*C. betulus*	0.7060	0.4899
Ash	*F. excelsior*	0.5597	0.4918
European beech	*F. sylvatica*	0.5855	0.4709
Sessile oak	*Q. petraea*	0.5597	0.4970
English oak	*Q. robur*	0.5597	0.5016
White oak	*Q. pubescens*	0.5597	0.4948
Other broadleaved	*Fagacea* spp	0.5672	0.4924

Note: General recommended factors are 0.5 t m^−3^ for conifers/evergreen and 0.6–0.7 t m^−3^ for broadleaves/deciduous. The carbon content for all tree organs (different tree compartments), can be estimated with a factor of 0.5, by neglecting the lower carbon concentration in the needles/leaves [12].

## 2 Experimental design, materials, and methods

The presented data is used to inform the models described in the following sub-sections.

### 2.1 Modelling non-linear growth

The cumulative tree growth is represented by the non-linear Chapman-Richards (CR) curve. The CR equation (Eq. (1)) is based on species- and site-dependent parameters and one independent variable, with the following notation [13]:

(1) $$\begin{array}{l} \omega \left(t_{i}\right)=\;A{\left(1-\beta exp^{-kt}\right)}^{p}+\text{ε}\;\\ \text{with}\;p=1/\left(1-m\right) \end{array}$$

where ω expresses the potential growth of a tree species i in height and circumference (response growth variables) at age t (independent variable), A,β,k,p are parameters, exp is the basis of natural logarithm and Ɛ the term for random error; with β is fixed to 1 [14], and the allometric constant m fixed to 0.5 (0<m<1) [13]. CR forms a sigmoid and asymptotic curve with a point of inflection determined by the allometric constant p, approaching a maximum threshold of the response variable, the asymptote A. The empirical growth parameter k scales the absolute growth, governing the rate at which A approaches its potential maximum.

### 2.2 Initial parameters to fit non-self-starting non-linear regression model

The statistical model using the CR curve $[\omega \;∼\;f\left(t_{i},\;\;\theta \right)+\varepsilon$] fits the vector of parameters θ to the growth variable ω; whereby the function f represents a non-linear combination of the parameters. Initial parameters to fit the non-self-starting non-linear regression model (Table 6) were developed for k andp. Values for k lie between 0.02 and 0.04, depending on the studied species and for p 2. The acceptable values for *k* range between 0.2 and 2.5. A is estimated as twice the maximum value given for age in the species-specific yield tables.

**Table 6:** Initial parameter for Chapman-Richards non-linear regression.

Common name	Species	Initial parameters		
		A	k	p
Douglas fir	*P. menziesii*	140	0.03	2
Norway spruce	*P. abies*	172	0.03	2
Maritime pine	*P. pinaster*	140	0.03	2
Silver fir	*A. alba*	326	0.03	2
Scots pine	*P. sylvestri*	180	0.03	2
Other conifers	*Pinaceae* spp	172	0.03	2
(Sweet) Chestnut	*C. sativa*	120	0.03	2
Hornbeam	*C. betulus*	200	0.02	2
Ash	*F. excelsior*	320	0.03	2
European Beech	*F. sylvatica*	300	0.02	2
White oak	*Q. petraea*	240	0.04	2
English oak	*Q. robur*	320	0.02	2
Sessile oak	*Q. pubescens*	400	0.04	2
Other broadleaves	*Fagacea* spp	300	0.04	2

Sources: A. Pommerening, pers. comm.; H. Pretzsch, pers. comm.

### 2.3 Allometric equations and specifications

Allometric models presented in Table 7 are used for tree volume estimation.

**Table 7:** Overview of retained allometric equations for volume estimations.

Species	Allometric equation	Coefficients					Volume	Location	Creator	Source
		α	β	γ	δ	ε				
*P. menziesii*	${\text{V}}_{T_{above}}=\text{(}\text{a}+\text{β}\times \text{Ci}\text{)}\times \text{(}1+\text{δ}/\text{(}{\text{Ci}}^{2}\text{)}\text{)}\times {\text{Ci}}^{2}\times \text{H}/\text{(}4\text{x}10^{4}\times \text{π}$)	5.3E-1	−5.3E-4	–	5.7E+1	–	Total AG	FRA	INRA	[15]
*P. abies*	${\text{V}}_{T_{above}}=\text{(}\text{a}+\text{β}\times \text{Ci}\text{)}\times {\text{Ci}}^{2}\times \text{H}/\text{(}4\text{x}10^{4}\times \text{π}$)	6.3E-1	−9.5E-4	–	–	–	Total AG	FRA	INRA	[15]
*P. pinaster*	${\text{V}}_{T_{above}}=\text{(}\text{a}+\text{β}\times \text{Ci}\text{)}+\text{γ}\;\;\times {\text{Ci}}^{\frac{1}{2}/\text{H}}\times \text{(}1+\text{(}\delta /Ci^{2}\text{)}\text{)}\times {\;\text{Ci}}^{2}\;\times \text{H}/\text{(}4E+04^{4}\times \text{π}\text{)}$	2.4E-1	9.7E-4	4.0E-1	2E+2	–	Total AG	FRA	INRA	[15]
*A. alba*	${{\text{V}}_{\text{stem}}=\text{(}{\text{a}+\text{β}\times \text{(}\text{Ci}/\text{π}\text{)}}^{2}\text{)}\;\times \;\text{H}+\;\text{γ}\times \text{(}\text{Ci}/\text{π}\text{)}}^{2}$	−2.8E+0	3.4E-2	8.4E-2	–	–	Stem UB	ITA	CMCC	[16]
*P. sylvestri*	${\text{V}}_{T_{above}}=\text{(}\text{a}+\text{β}\times \text{Ci}\text{)}+\text{γ}\;\;\times {\text{Ci}}^{\frac{1}{2}/\text{H}}\times \text{(}1+\text{(}\delta /Ci^{2}\text{)}\text{)}\;\times {\;\text{Ci}}^{2}\;\times \text{H}/\text{(}4\text{x}10^{4}\times \text{π}\text{)}$	3.0E-1	3.2E-4	3.8E-1	2E+2	–	Total AG	FRA	INRA	[15]
*Pinaceae* spp	${\text{V}}_{T_{above}}=\text{(}\text{a}+\text{β}\times \text{Ci}\text{)}+\text{γ}\;\;\times {\text{Ci}}^{\frac{1}{2}/\text{H}}\times \text{(}1+\text{(}\delta /Ci^{2}\text{)}\text{)}\;\times {\;\text{Ci}}^{2}\;\times \text{H}/\text{(}4\text{x}10^{4}\times \text{π}\text{)}$	3.0E-1	3.2E-4	3.8E-1	2E+2	–	Total AG	FRA	INRA	[15]
*C. sativa*	${{\text{V}}_{\text{stem}}=\;\text{α}\;\times \text{(}Ci/\pi \text{)}}^{2}\times \text{H}+\text{β}$	3.8E-2	8.5E-1	–	–	–	Stem UB	FRA	FCBA	[17]
*C. betulus*	${{\text{V}}_{\text{stem}}=\;\text{α}\;\times \text{(}Ci/\pi \text{)}}^{2}\times \text{H}+\text{β}$	3.3E-2	3.0E+0	–	–	–	Stem UB	FRA	FCBA	[17]
*F. excelsior*	${{\text{V}}_{\text{stem}}=\text{(}Ci/\pi \text{)}}^{\text{α}}\times {\text{H}}^{\text{β}}\times {\;\text{e}}^{-\text{γ}}$	2.0E+0	7.7E-1	2.5E+0	–	–	Stem UB	NDL	CMCC	[18]
*F. sylvatica*	${\text{V}}_{T_{above}}=\text{(}\text{a}+\text{β}\times \text{Ci}\text{)}+\text{γ}\;\;\times {\text{Ci}}^{\frac{1}{2}/\text{H}}\times \text{(}1+\text{(}\delta /Ci^{2}\text{)}\text{)}\;\times {\;\text{Ci}}^{2}\;\times \text{H}/\text{(}4\text{x}10^{4}\times \text{π}\text{)}$	4.0E-1	2,7E-4	4.2E-1	4.5E+1	–	Total AG	FRA	INRA	[15]
*Q. petraea*	${\text{V}}_{T_{above}}=\text{(}\text{a}+\text{β}\times \text{Ci}\text{)}+\text{γ}\;\;\times {\text{Ci}}^{\frac{1}{2}/\text{H}}\times \text{(}1+\text{(}\delta /Ci^{2}\text{)}\text{)}\;\times {\;\text{Ci}}^{2}\;\times \text{H}/\text{(}4\text{x}10^{4}\times \text{π}\text{)}$	4.7E-1	−3.5E-4	3.8E-1	–	–	Total AG	FRA	INRA	[15]
*Q. robur*	${{\text{V}}_{\text{stem}}=\text{(}Ci/\pi \text{)}}^{\text{α}}\times {\text{H}}^{\text{β}}\times {\;\text{e}}^{-\text{γ}}$	2.0E+0	8.6E-1	2.9E+0	–	–	Stem UB	NDL	CMCC	[18]
*Q. pubescens*	${{\text{V}}_{\text{stem}}=\;\text{α}\;\times 10^{\text{(}\text{β}\times \text{LOG}\text{(}\text{Ci}/\text{π}\text{)}\text{)}}+\text{γ}\times \text{LOG}\text{(}Ci/\pi \text{)}}^{2}\times +\text{δ}\times \text{LOG}\text{(}\text{H}\text{)}+\;\text{ε}\times \text{LOG}{\text{(}\text{H}\text{)}}^{2}$	3.5E-4	1.1E+0	3.1E-1	5.4E-1	2.1E-1	Stem UB	ROU	CMCC	[19]
*Fagacea* spp	${\text{V}}_{T_{above}}=\text{(}\text{a}+\text{β}\times \text{Ci}\text{)}+\text{γ}\;\;\times {\text{Ci}}^{\frac{1}{2}/\text{H}}\times \text{(}1+\text{(}\delta /Ci^{2}\text{)}\text{)}\times {\;\text{Ci}}^{2}\;\times \text{H}/\text{(}4\text{x}10^{4}\times \text{π}\;\text{)}$	4.7E-1	3.5E-4	3.8E-1	–	–	Total AG	FRA	INRA	[15]

Acronyms: H: top height; DBH: Diameter breast height; Ci: Circumference; Total AG: total aboveground; Stem UB: stem under bark; FRA: France; ITA: Italy; NDL: Netherlands; ROU: Romania.

Note: Equations are all expressed in Ci and the given units needed respective conversions to be expressed in common units. The volume is expressed in stem under bark (i.e. bark and wood) or total aboveground tree volume. The total aboveground volume includes stem under bark, needles/leaves and branches. The group “other conifers” (*Pinaceae* spp*)* and “other broadleaved” (*Fagacea* spp*)* use the same volume relations as Scots pine and Sessile oak respectively, due to their representativeness.

Source: Allometric equations analysed and selected from Ref. [6]; and respective references in the table.

### 2.4 Mean biomass growth development of all species

**Fig. 1:**
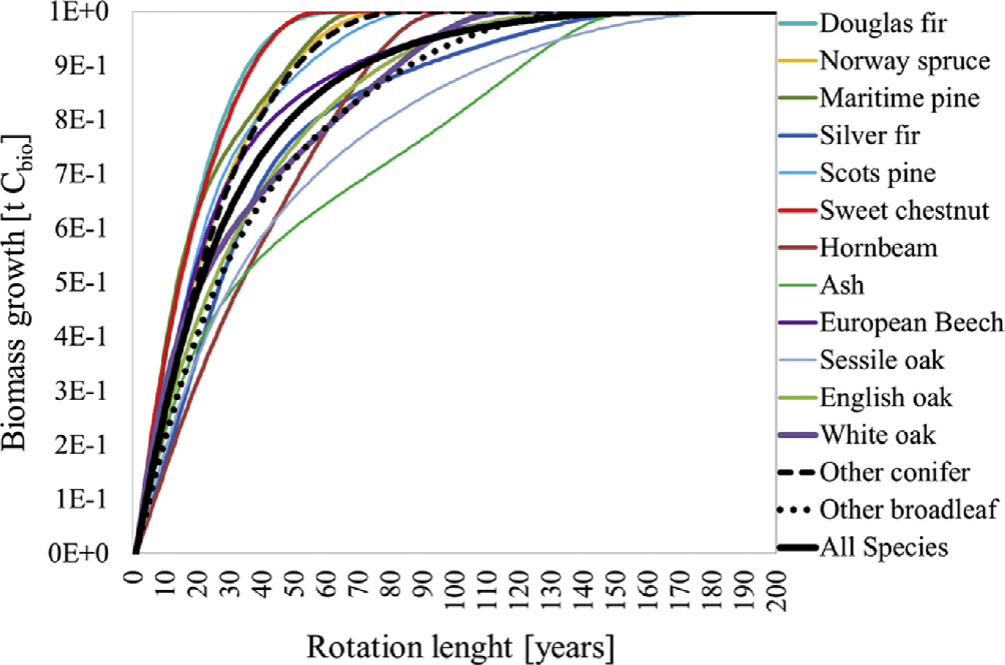
Mean biomass growth in tonnes of carbon per tree species.

Fig. 1 shows the non-linear mean biomass growth per tree species. For the computation of annual C_bio_ fixation flows [t C_bio_·yr^−1^] in biomass (as presented with the stocking factors in the *Supplementary material*) see section 2.3.1. in the companion research article [1]. Data from Table 3 to Table 7 are used for these calculations.
