# Supplementary material for: Data and non-linear models for the estimation of biomass growth and carbon fixation in managed forests
Source: Data Brief. 2019 Mar 16;23:103841. doi: 10.1016/j.dib.2019.103841 (PMC6660611; doi:10.1016/j.dib.2019.103841)
Supplement: Supplementary file 1 — Multimedia component 1 [file mmc1.pdf]

Conflict of Interest and Authorship Conformation Form

Please check the following as appropriate:

- o All authors have participated in (a) conception and design, or analysis and interpretation of the data; (b) drafting the article or revising it critically for important intellectual content; and (c) approval of the final version.
- o This manuscript has not been submitted to, nor is under review at, another journal or other publishing venue.
- o The authors have no affiliation with any organization with a direct or indirect financial interest in the subject matter discussed in the manuscript
- o The following authors have affiliations with organizations with direct or indirect financial interest in the subject matter discussed in the manuscript:

Author's name and Affiliations

Ariane ALBERS (corresponding author)
IFP Energies Nouvelles, 1 et 4 Avenue de Bois-Préau, 92852 Rueil-Malmaison, France
LBE, Montpellier SupAgro, INRA, UNIV Montpellier, Narbonne, France
Elsa, Research Group for Environmental Lifecycle and Sustainability Assessment, Montpellier, France
Pierre COLLET
IFP Energies Nouvelles, 1 et 4 Avenue de Bois-Préau, 92852 Rueil-Malmaison, France
Anthony BENOIST
Elsa, Research Group for Environmental Lifecycle and Sustainability Assessment, Montpellier, France
CIRAD – UPR BioWooEB, Avenue Agropolis, F-34398 Montpellier, France
Arnaud HELIAS
LBE, Montpellier SupAgro, INRA, UNIV Montpellier, Narbonne, France
Elsa, Research Group for Environmental Lifecycle and Sustainability Assessment, Montpellier, France
Chair of Sustainable Engineering, Technische Universität Berlin, Berlin, Germany

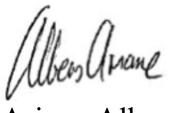

Ariane Albers
