# Supplementary material for: Data and non-linear models for the estimation of biomass growth and carbon fixation in managed forests
Source: Data Brief. 2019 Mar 16;23:103841. doi: 10.1016/j.dib.2019.103841 (PMC6660611; doi:10.1016/j.dib.2019.103841)
Supplement: Multimedia component 2 [file mmc2.docx]

Supplementary Material for:

Data and non-linear models for the estimation of biomass growth and carbon fixation in managed forests

Ariane Albers ^1,2,3,^*, Pierre Collet ^1^, Anthony Benoist ^3,4^, Arnaud Hélias ^2,3,5^

^1^ IFP Energies Nouvelles, 1 et 4 Avenue de Bois-Préau, 92852 Rueil-Malmaison, France

^2^ LBE, Montpellier SupAgro, INRA, UNIV Montpellier, Narbonne, France

^3^ Elsa, Research Group for Environmental Lifecycle and Sustainability Assessment, Montpellier, France

^4^ CIRAD – UPR BioWooEB, Avenue Agropolis, F-34398 Montpellier, France

^5^ Chair of Sustainable Engineering, Technische Universität Berlin, Berlin, Germany

* Corresponding author. E-mail address: [ariane.albers@ifpen.fr](mailto:ariane.albers@ifpen.fr)

# Diagram for modelling biomass growth and carbon fixation


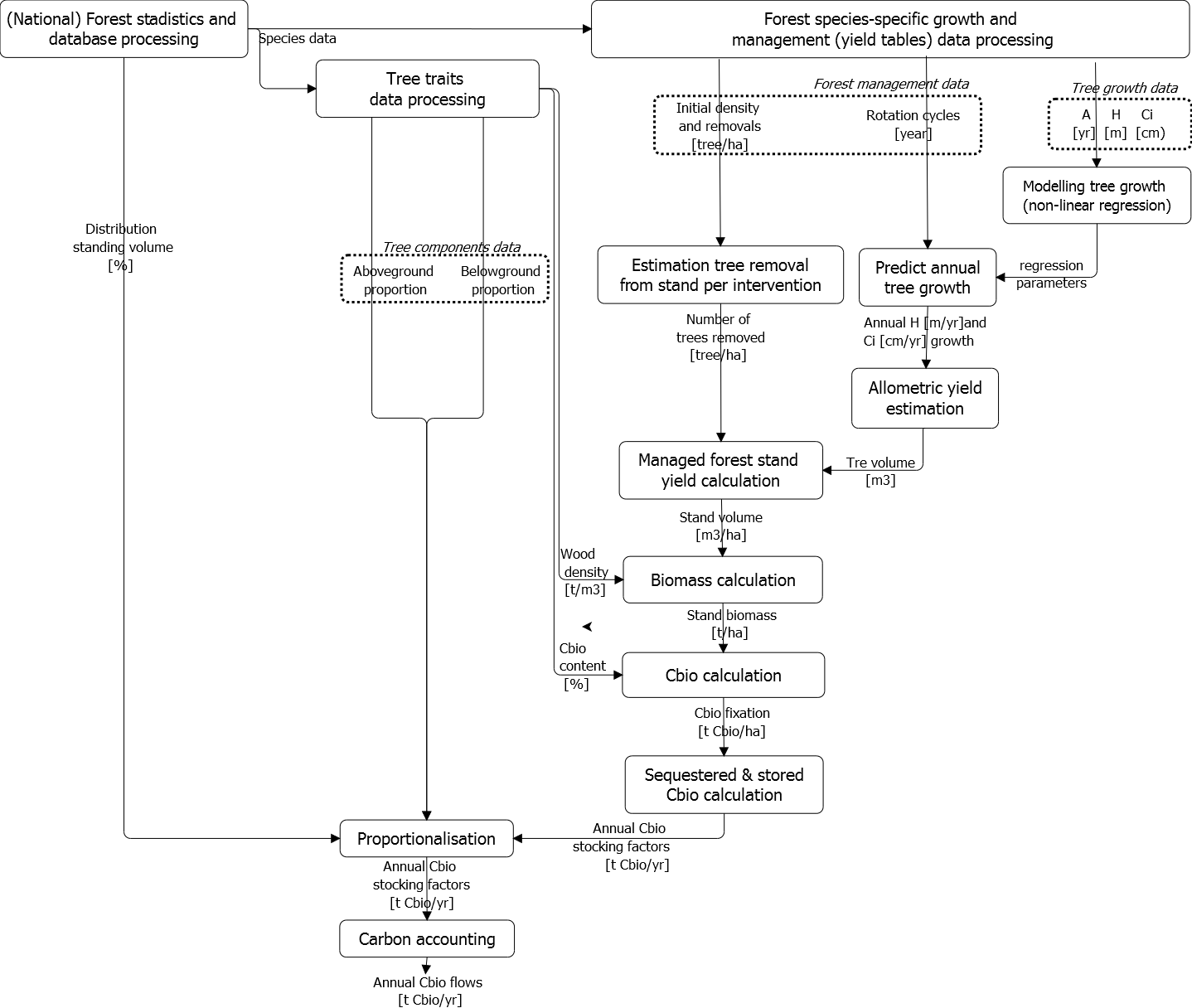


Figure 1: Detailed diagram for computing biogenic carbon (C_bio_) stocking in forestry biomass with a dynamic C_bio_ model

# The non-linear Chapman Modelling growth model

The cumulative tree growth and yield development is a function of age-to-height and age-to-diameter-breast-height or age-to-circumference. Tree growth eventually reaches a threshold, when decline and growth reach equilibrium [1]. This threshold is asymptotic. The Chapman-Richards (CR) model can represent this non-linear growth curve [2,3]. It forms a sigmoid and asymptotic curve with a point of inflection commonly present in biological growth models [4]. The equation is applicable to both individual mean tree development and whole tree population of a forest stand, and is therefore largely used and recommended in empirical forest studies to model site-specific tree development [1,3–8]. The CR model is known for being very flexible and accurate, yet on the “slight expense of biological realism” [9].

# R script for the non-linear regression analysis of tree growth

An R [10] script facilitates computing the regression parameters for running the growth model. The statistic R^2^ is not recommended for evaluating the goodness of fit of non-linear regression models [11,12], thus simple correlation between yield table and predicted values was used [11].

R script including comments:

# Include full route to the target source file containing a yield table (columns: age, height, circumference)

route = "//FILENAME.csv"

# Name the selected species

species = "SPECIESNAME"

# Loading yield table data from a CSV

mydata<-read.table(paste(" ", route, sep=""), header=TRUE, sep=",", dec=".")

age <- c(mydata[1])

age <- as.numeric(unlist(age))

topHeight <- c(mydata[2])

topHeight <- as.numeric(unlist(topHeight))

circonf <- c(mydata[3])

circonf <- as.numeric(unlist(circonf))

# Adjust species-dependent k and p parameter initial values (example k and p values for Douglas fir)

A_init <- 2*max(circonf)

k_init <- 0.03

p_init <- 2

# Predict age-circumference with Chapman-Richards model (adapted from [9])

nlsfitSS <- nls(circonf ~ A *(1 - exp(-k * age))^p,

data=mydata,

start = list(A = A_init, k = k_init, p = p_init))

nlsfitSS

# Estimate goodness of fit, by calculating correlation between the yield table values and the predicted values

# R^2 is not recommended for non-linear regressions: [11]

GoF <- cor(circonf,predict(nlsfitSS))

GoF

# Extrapolate function to an advanced age, to visualise the asymptote

age_as <- 500

predCirconf <- coef(nlsfitSS)[1] * (1-exp(-coef(nlsfitSS)[2] *age_as)) ^ coef(nlsfitSS)[3]

predCirconf

# Plot results and asymptote

x <- 1:age_as

y <- coef(nlsfitSS)[1] * (1-exp(-coef(nlsfitSS)[2] * x)) ^ coef(nlsfitSS)[3]

plot(x, y, col="red", xlab="Age (years)", ylab="Circumference (cm)") #type="o"

lines(age, circonf, col="black", type="o", lwd = 2)

title(c("Tree circumference (mean from fitted data): ", species))

mylabel1 = bquote(A:asymptote == .(format(coef(nlsfitSS)[1], digits = 4)))

mylabel2 = bquote(k == .(format(coef(nlsfitSS)[2], digits = 3)))

mylabel3 = bquote(p == .(format(coef(nlsfitSS)[3], digits = 3)))

mylabel4 = bquote(GoF (correlation) == .(format(GoF, digits = 3)))

# Adjust label positions

text(x = max(x)-100, y = max(y)-120, labels = mylabel1)

text(x = max(x)-100, y = max(y)-130, labels = mylabel2)

text(x = max(x)-100, y = max(y)-140, labels = mylabel3)

text(x = max(x)-100, y = max(y)-150, labels = mylabel4)

# Adjust species-dependent k and p parameter initial values (example k and p values for Douglas fir)

A_init <- 2*max(topHeight)

k_init <- 0.03

p_init <- 2

# Predict age-topHeight with Chapman-Richards model

nlsfitSS <- nls(topHeight ~ A * (1 - exp(-k * age))^p,

data=mydata,

start = list(A = A_init, k = k_init, p = p_init))

nlsfitSS

# Estimate goodness of fit, by calculating correlation between the yield table values and the predicted values

# R^2 is not recommended for non-linear regressions: [11] and <https://www.researchgate.net/post/How_to_assess_goodness_of_fit_for_a_non-linear_model>

GoF <- cor(topHeight, predict(nlsfitSS))

GoF

# open second plot window

dev.new()

# plot results and asymptote

x <- 1:500

y <- coef(nlsfitSS)[1] * (1-exp(-coef(nlsfitSS)[2] * x)) ^ coef(nlsfitSS)[3]

plot(x, y, col="red", xlab="Age (years)", ylab="Height (m)") #type="o"

lines(age, topHeight, col="black", type="o", lwd = 2)

# Adjust label positions

title(c("Tree height (mean from fitted data): ", species))

mylabel1 = bquote(A:asymptote == .(format(coef(nlsfitSS)[1], digits = 4)))

mylabel2 = bquote(k == .(format(coef(nlsfitSS)[2], digits = 3)))

mylabel3 = bquote(p == .(format(coef(nlsfitSS)[3], digits = 3)))

mylabel4 = bquote(GoF (correlation) == .(format(GoF, digits = 3)))

text(x = max(x)-100, y = max(y)-12, labels = mylabel1)

text(x = max(x)-100, y = max(y)-14, labels = mylabel2)

text(x = max(x)-100, y = max(y)-16, labels = mylabel3)

text(x = max(x)-100, y = max(y)-18, labels = mylabel4)

# Details of biogenic carbon modelling per species

The presented data in this section describes growth and biogenic carbon fixation (C_bio_) of each analysed forest tree species used by the methods and models described in the following sub-sections. The stocking factors are applicable for historic modelling approaches, it is to say rotation cycles taking place before final harvest.

## Douglas fir (*Pseudotsuga menziesii*)

Representation in France

- 1827 firstly introduced in France [13]. The forest stands in France are young: 87% of stands do not exceed 40 years old [14]
- 80% of Douglas fir forestland is concentrated in the mid-mountain territory of the Massif Central: La Bourgogne, Le Limousine, Auvergne, Rhône-Alpes et Midi-Pyrénées [15]
- Today France belongs to the main Douglas fir producers in Europe with standing volume of almost 120 Mm^3^ distributed over 400 000 hectares [16].
- The annual biological growth of the standing volume is estimated at about 6 Mm^3^ [17].

Regression analysis

Age-height and age-circumference mean tree growth development provided in the yield table of the experimental silviculture plot (2.0 x 2.0 m) by Décourt (1972) [18] at the administrative departments Creuse, Corrèze et Haute-Vienne situated in the West part of the Massif Central (375-915 m of altitude). Data for growth estimations taken from productivity class 2 (out of three yield classes), listed in Table 1 .

Table 1: Mean growth of Douglas fir (*p. menziesii*), productivity class 2, West of Massif Central

| Year | Top height | Circumference |
| --- | --- | --- |
| [yr] | [m] | [cm] |
| 15 | 11.2 | 60.7 |
| 20 | 14.8 | 73.4 |
| 25 | 18.2 | 85.2 |
| 30 | 21.2 | 96.4 |
| 35 | 24.0 | 107 |
| 40 | 26.6 | 116.9 |
| 45 | 29.0 | 126.3 |
| 50 | 31.2 | 135.3 |
| 55 | 33.2 | 143.7 |
| 60 | 35.1 | 151.7 |
| 65 | 36.9 | 159.1 |
| Source : [11, p.50] | | |

Model parameter results are given in Table 2. The goodness of fit (GoF) for both age-height and age-circumference suggests a perfect fit. Results are plotted in Figure 2 and Figure 3, showing a comparison of the GoF from experimental yield table values and the fitted model.

Table 2: Characteristics and model fitting data of Douglas fir (*p. menziesii*)

|  | A | k | p | RSS | GoF |
| --- | --- | --- | --- | --- | --- |
| Age-height growth | 54.82308 | 0.01854 | 1.11732 | 0.02671 | 1 |
| Age-circumference growth | 455.700 | 0.0040 | 0.7092 | 0.4748 | 1 |

| 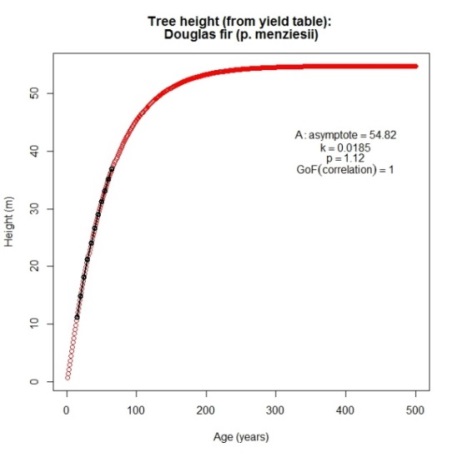 | 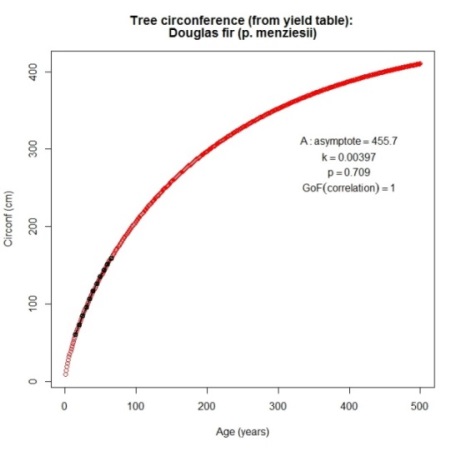 |
| --- | --- |
| Figure 2: Age-height mean growth curve and model parameter results of Douglas fir (*p. menziesii*). Black dots represent yield table data, red circles model-predicted values | Figure 3: Age-circumference mean growth curve model parameter results of Douglas fir (*p. menziesii*). Black dots represent yield table data, red circles model-predicted values |

Biomass growth

Cutting/thinning operations in accordance with the yield table (Table 3) to represent growth and carbon sequestration dynamics per species and year. Initial density from seedlings is 2100 stems and first thinning period at age 25 with five-year rotation cycles. The dominant height is calculated from the Chapman-Richards model by non-linear regression. The total rotation length is 70 years which also represents the year of final cut (clear cut of all remaining stands). In total 10 cutting interventions are performed (including final cut).

Table 3: Douglas fir (*p. menziesii*) forest management practices with rotation length of 70 years

|  |  | Age | Top height | Density before cut | Stems removed | Stems removed |
| --- | --- | --- | --- | --- | --- | --- |
|  |  | [yr] | [m] | [stem·ha^-1^] | [stem·ha^-1^] | [%] |
| Rotation length | 70 | - | - | - | - | - |
| Total interventions | 10 | - | - | - | - | - |
| Initial density | 2100 | - | - | - | - | - |
| Cut 1 | - | 25 | 18 | 1924 | 176 | 8% |
| Cut 2 | - | 30 | 21 | 1605 | 319 | 15% |
| Cut 3 | - | 35 | 24 | 1297 | 308 | 15% |
| Cut 4 | - | 40 | 27 | 1056 | 241 | 11% |
| Cut 5 | - | 45 | 29 | 856 | 200 | 10% |
| Cut 6 | - | 50 | 31 | 706 | 150 | 7% |
| Cut 7 | - | 55 | 33 | 594 | 112 | 5% |
| Cut 8 | - | 60 | 35 | 510 | 84 | 4% |
| Cut 9 | - | 65 | 37 | 454 | 56 | 3% |
| Final clear cut | - | 70 | 38 | 0 | 454 | 22% |
| Source: [11, p.50] and fitted height values | | | | | | |

Aboveground volume estimations based on allometric equation and coefficients. Growth dynamics of individual stem volume (Figure 4) and managed stand (Figure 5), with 3.8 m^3^ and 1724 m^3^·ha^-1^ respectively over a 70-years rotation cycle.

| 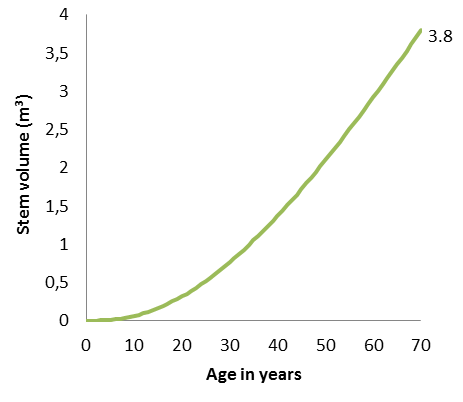 | 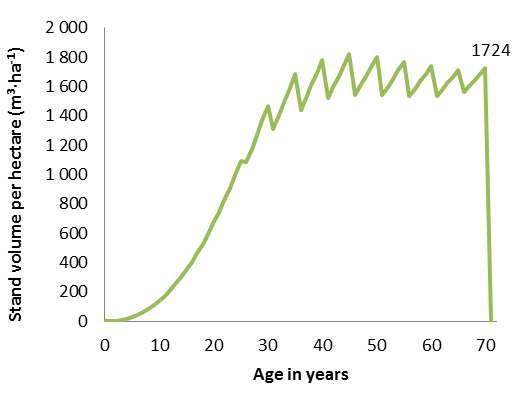 |
| --- | --- |
| Figure 4: Douglas fir (*p. mensiezii*) mean aboveground individual stem volume (m^3^) over 70-year rotation cycle | Figure 5: Douglas fir (*p. mensiezii*) mean aboveground stand volume per hectare (m^3^·ha^-1^) with thinning periods and clear-cut at age 70 |

Carbon fixation and stocking

Carbon sequestration and storage of Douglas fir over a 70-year rotation cycle (Table 4). Stocking factors applicable for rotation cycles before harvest (historic modelling approach).

Table 4: C_bio_ stocking factors for Douglas fir (*p. menziesii*), 70-year rotation cycle

| Age  [yr] | C_bio_  growth  [t C_bio]_ | Annual C_bio_  stocking factors  [t C_bio_·yr^-1^] | Age  [yr] | C_bio_  growth  [t C_bio]_ | Annual C_bio_ stocking factors  [t C_bio_·yr^-1^] | Age  [yr] | C_bio_  growth  [t C_bio]_ | Annual C_bio_ stocking factors  [t C_bio_·yr^-1^] |
| --- | --- | --- | --- | --- | --- | --- | --- | --- |
| 0 | 0.000000 | 0.044427 | 30 | 0.844380 | 0.012815 | 60 | 0.998181 | 0.000445 |
| 1 | 0.044427 | 0.043257 | 31 | 0.857195 | 0.012060 | 61 | 0.998627 | 0.000360 |
| 2 | 0.087684 | 0.042085 | 32 | 0.869255 | 0.011330 | 62 | 0.998987 | 0.000287 |
| 3 | 0.129769 | 0.040912 | 33 | 0.880585 | 0.010624 | 63 | 0.999274 | 0.000226 |
| 4 | 0.170680 | 0.039739 | 34 | 0.891209 | 0.009942 | 64 | 0.999500 | 0.000175 |
| 5 | 0.210419 | 0.038566 | 35 | 0.901151 | 0.009258 | 65 | 0.999675 | 0.000108 |
| 6 | 0.248985 | 0.037395 | 36 | 0.910409 | 0.008630 | 66 | 0.999782 | 0.000080 |
| 7 | 0.286380 | 0.036226 | 37 | 0.919039 | 0.008027 | 67 | 0.999862 | 0.000059 |
| 8 | 0.322606 | 0.035061 | 38 | 0.927066 | 0.007447 | 68 | 0.999922 | 0.000045 |
| 9 | 0.357667 | 0.033901 | 39 | 0.934513 | 0.006891 | 69 | 0.999966 | 0.000034 |
| 10 | 0.391569 | 0.032747 | 40 | 0.941405 | 0.006334 | 70 | 1.000000 | 0.000000 |
| 11 | 0.424316 | 0.031600 | 41 | 0.947738 | 0.005832 | 61 | 0.998181 | 0.000445 |
| 12 | 0.455915 | 0.030461 | 42 | 0.953570 | 0.005353 | 62 | 0.998627 | 0.000360 |
| 13 | 0.486376 | 0.029331 | 43 | 0.958923 | 0.004898 | 63 | 0.998987 | 0.000287 |
| 14 | 0.515707 | 0.028213 | 44 | 0.963821 | 0.004465 | 64 | 0.999274 | 0.000226 |
| 15 | 0.543920 | 0.027106 | 45 | 0.968286 | 0.004030 | 65 | 0.999500 | 0.000175 |
| 16 | 0.571026 | 0.026013 | 46 | 0.972316 | 0.003649 | 66 | 0.999675 | 0.000108 |
| 17 | 0.597040 | 0.024936 | 47 | 0.975965 | 0.003290 | 67 | 0.999782 | 0.000080 |
| 18 | 0.621975 | 0.023875 | 48 | 0.979255 | 0.002954 | 68 | 0.999862 | 0.000059 |
| 19 | 0.645850 | 0.022832 | 49 | 0.982208 | 0.002638 | 69 | 0.999922 | 0.000045 |
| 20 | 0.668682 | 0.021808 | 50 | 0.984846 | 0.002318 | 70 | 0.999966 | 0.000034 |
| 21 | 0.690490 | 0.020806 | 51 | 0.987164 | 0.002050 |  |  |  |
| 22 | 0.711297 | 0.019827 | 52 | 0.989214 | 0.001802 |  |  |  |
| 23 | 0.731123 | 0.018871 | 53 | 0.991015 | 0.001574 |  |  |  |
| 24 | 0.749994 | 0.017937 | 54 | 0.992589 | 0.001364 |  |  |  |
| 25 | 0.767931 | 0.017002 | 55 | 0.993954 | 0.001147 |  |  |  |
| 26 | 0.784934 | 0.016122 | 56 | 0.995101 | 0.000980 |  |  |  |
| 27 | 0.801056 | 0.015266 | 57 | 0.996081 | 0.000829 |  |  |  |
| 28 | 0.816322 | 0.014434 | 58 | 0.996911 | 0.000695 |  |  |  |
| 29 | 0.830755 | 0.013625 | 59 | 0.997606 | 0.000576 |  |  |  |

## Norway Spruce (*Picea abies*)

Regression analysis

Age-height and age-circumference mean tree growth development provided in yield tables of experimental silviculture plots by P. Deplat and R. Bolliet (1979) [18] at the South of Massif Central (Montagne Noire, Monts de Lacune-Sommail-Espinouse, Levezou and Aigoual). Data for growth estimations taken from productivity class 16 (out of nine yield classes) with reference height of 23.4 m, shown in Table 5.

Table 5: Mean growth of Norway spruce (*p. abies*), productivity class 16, South of Massif Central

| Year | Top height | Diameter breast height | Circumference |
| --- | --- | --- | --- |
| [yr] | [m] | [cm] | [cm] |
| 30 | 13.1 | 22 | 69.1 |
| 37 | 17.1 | 28 | 88.0 |
| 44 | 20.6 | 33 | 103.7 |
| 51 | 23.5 | 38 | 119.4 |
| 58 | 25.9 | 42 | 131.9 |
| 65 | 28.0 | 46 | 144.5 |
| 72 | 29.8 | 50 | 157.1 |
| 79 | 31.3 | 53 | 166.5 |
| 86 | 32.7 | 56 | 175.9 |
| Source: [11, p. 134] | | | |

Model parameter results are illustrated in Table 6. The goodness of fit (GoF) for both age-height and age-circumference corresponds to 1. Results are plotted in Figure 6 and Figure 7, showing a comparison of the GoF from experimental yield table values and the fitted model.

Table 6: Characteristics and model fitting data of Norway spruce (*p. abies*)

|  | A | k | p | RSS | GoF |
| --- | --- | --- | --- | --- | --- |
| Age-height growth | 37.6413 | 0.0317 | 2.1372 | 0.09754 | 1 |
| Age-circumference growth | 246.1658 | 0.0185 | 1.4775 | 2.527 | 1 |

| 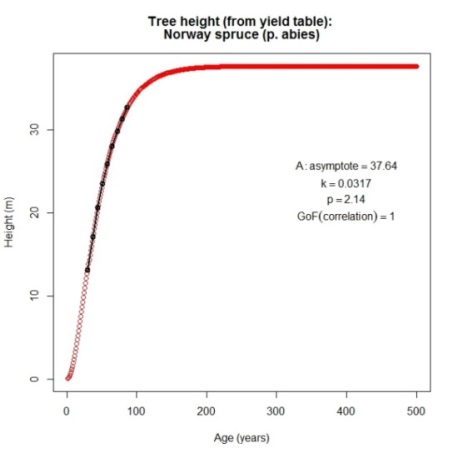 | 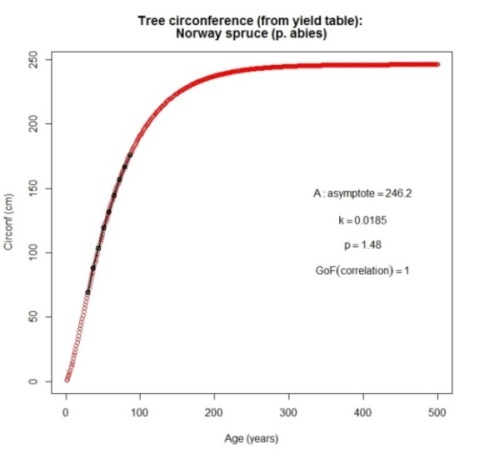 |
| --- | --- |
| Figure 6: Age-height mean growth curve and model parameter results of Norway spruce (p. abies). Black dots represent yield table data, red circles model-predicted values | Figure 7: Age-circumference mean growth curve and model parameter results of Norway spruce (*p. abies*). Black dots represent yield table data, red circles model-predicted values |

Biomass growth

Cutting/thinning operations in accordance with the yield table (Table 7) to represent growth and carbon sequestration dynamics per species and year. Initial density from seedlings is 2100 stems and first thinning period at age 30 with seven-year rotation cycles. The dominant height is calculated from the Chapman-Richards model by non-linear regression. The total rotation length is 86 years which also represents the year of final cut (clear cut of all remaining stands). In total 9 cutting interventions are performed (including final cut).

Table 7: Even-aged forest stand forest management practices with rotation length of 86 years

|  |  |  | | Age | Top height | Density before cut | Stems removed | Stems removed |
| --- | --- | --- | --- | --- | --- | --- | --- | --- |
|  |  |  |  | [yr] | [m] | [stem·ha^-1^] | [stem·ha^-1^] | [%] |
| Rotation length | 86 | | - | | - | - | - | - |
| Total thinning operations | 9 | | - | | - | - | - | - |
| Initial density | 2100 | | - | | - | - | - | - |
| Cut 1 | - | | 30 | | 13 | 1417 | 683 | 33% |
| Cut 2 | - | | 37 | | 17 | 1093 | 324 | 15% |
| Cut 3 | - | | 44 | | 20 | 844 | 249 | 12% |
| Cut 4 | - | | 51 | | 23 | 651 | 193 | 9% |
| Cut 5 | - | | 58 | | 26 | 502 | 149 | 7% |
| Cut 6 | - | | 65 | | 28 | 389 | 113 | 5% |
| Cut 7 | - | | 72 | | 30 | 299 | 90 | 4% |
| Cut 8 | - | | 79 | | 31 | 231 | 68 | 3% |
| Final clear cut | - | | 86 | | 33 | 0 | 231 | 11% |
| Source: [11, p. 134] | | | | | | | | |

Aboveground volume estimations based on allometric equation and coefficients. Growth dynamics of individual stem volume (Figure 8) and managed stand (Figure 9), with 0.84 m^3^ and 194 m^3^·ha^-1^ respectively over 86-years rotation cycle.

| 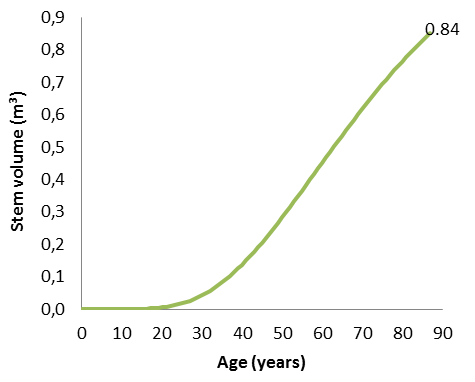 | 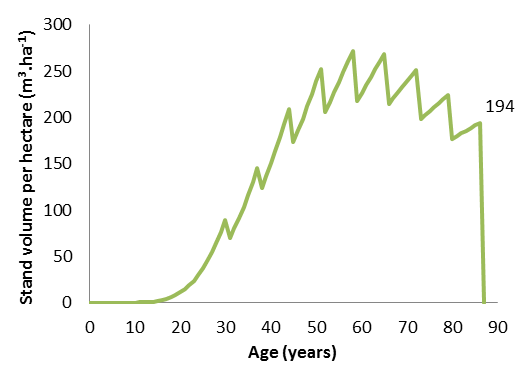 |
| --- | --- |
| Figure 8: Norway spruce (*p. abies*) mean aboveground individual stem volume (m^3^) over 86-year rotation cycle | Figure 9: Norway spruce (*p. abies*) mean aboveground volume per hectare (m^3^·ha^-1^) with thinning periods and clear cut at age 86 |

Carbon fixation

Carbon sequestration and storage of Norway spruce (*p. abies*) over a 86-year rotation cycle (Table 8). Stocking factors applicable for rotation cycles before harvest (historic modelling approach).

Table 8: Cbio stocking factors for Norway spruce (p. abies), 86-year rotation cycle

| Age  [yr] | Cbio  growth  [t C_bio_] | Annual Cbio stocking factors  [t C_bio_·yr^-1^] | Age  [yr] | Cbio  growth  [t Cbio] | Annual Cbio stocking factors  [t Cbio·yr-1] | Age  [yr] | Cbio  growth  [t Cbio] | Annual Cbio stocking factors  [t C_bio_·yr-1] |
| --- | --- | --- | --- | --- | --- | --- | --- | --- |
| 0 | 0.000000 | 0.031460 | 36 | 0.786141 | 0.011217 | 72 | 0.994921 | 0.001119 |
| 1 | 0.031460 | 0.031249 | 37 | 0.797358 | 0.010833 | 73 | 0.996040 | 0.000944 |
| 2 | 0.062708 | 0.030994 | 38 | 0.808191 | 0.010456 | 74 | 0.996983 | 0.000781 |
| 3 | 0.093702 | 0.030693 | 39 | 0.818648 | 0.010088 | 75 | 0.997764 | 0.000632 |
| 4 | 0.124395 | 0.030348 | 40 | 0.828736 | 0.009731 | 76 | 0.998395 | 0.000498 |
| 5 | 0.154743 | 0.029957 | 41 | 0.838467 | 0.009385 | 77 | 0.998893 | 0.000380 |
| 6 | 0.184700 | 0.029521 | 42 | 0.847852 | 0.009048 | 78 | 0.999273 | 0.000279 |
| 7 | 0.214221 | 0.029040 | 43 | 0.856900 | 0.008721 | 79 | 0.999553 | 0.000194 |
| 8 | 0.243261 | 0.028517 | 44 | 0.865621 | 0.008398 | 80 | 0.999747 | 0.000126 |
| 9 | 0.271778 | 0.027951 | 45 | 0.874018 | 0.008079 | 81 | 0.999872 | 0.000073 |
| 10 | 0.299729 | 0.027346 | 46 | 0.882098 | 0.007766 | 82 | 0.999946 | 0.000037 |
| 11 | 0.327075 | 0.026704 | 47 | 0.889864 | 0.007460 | 83 | 0.999982 | 0.000014 |
| 12 | 0.353778 | 0.026027 | 48 | 0.897323 | 0.007160 | 84 | 0.999996 | 0.000003 |
| 13 | 0.379806 | 0.025320 | 49 | 0.904483 | 0.006867 | 85 | 1.000000 | 0.000000 |
| 14 | 0.405126 | 0.024587 | 50 | 0.911350 | 0.006578 |  |  |  |
| 15 | 0.429713 | 0.023832 | 51 | 0.917928 | 0.006292 |  |  |  |
| 16 | 0.453545 | 0.023062 | 52 | 0.924220 | 0.006008 |  |  |  |
| 17 | 0.476607 | 0.022281 | 53 | 0.930228 | 0.005727 |  |  |  |
| 18 | 0.498887 | 0.021495 | 54 | 0.935954 | 0.005449 |  |  |  |
| 19 | 0.520383 | 0.020713 | 55 | 0.941404 | 0.005176 |  |  |  |
| 20 | 0.541096 | 0.019940 | 56 | 0.946580 | 0.004907 |  |  |  |
| 21 | 0.561035 | 0.019183 | 57 | 0.951487 | 0.004641 |  |  |  |
| 22 | 0.580218 | 0.018448 | 58 | 0.956128 | 0.004377 |  |  |  |
| 23 | 0.598666 | 0.017743 | 59 | 0.960505 | 0.004114 |  |  |  |
| 24 | 0.616409 | 0.017071 | 60 | 0.964618 | 0.003853 |  |  |  |
| 25 | 0.633480 | 0.016437 | 61 | 0.968471 | 0.003596 |  |  |  |
| 26 | 0.649917 | 0.015842 | 62 | 0.972067 | 0.003343 |  |  |  |
| 27 | 0.665759 | 0.015287 | 63 | 0.975410 | 0.003095 |  |  |  |
| 28 | 0.681046 | 0.014765 | 64 | 0.978504 | 0.002851 |  |  |  |
| 29 | 0.695811 | 0.014271 | 65 | 0.981355 | 0.002611 |  |  |  |
| 30 | 0.710082 | 0.013793 | 66 | 0.983966 | 0.002375 |  |  |  |
| 31 | 0.723876 | 0.013327 | 67 | 0.986341 | 0.002145 |  |  |  |
| 32 | 0.737203 | 0.012874 | 68 | 0.988486 | 0.001922 |  |  |  |
| 33 | 0.750077 | 0.012437 | 69 | 0.990407 | 0.001707 |  |  |  |
| 34 | 0.762514 | 0.012016 | 70 | 0.992114 | 0.001501 |  |  |  |
| 35 | 0.774530 | 0.011610 | 71 | 0.993615 | 0.001305 |  |  |  |

## Maritime pine (*Pinus pinaster*)

Regression analysis

Age-height and age-circumference mean tree growth development provided in yield tables of experimental silviculture plots by N. Décourt et B. Lemoine (1969) [18] at the Landes de Gascogne in the South-West of France. Data for growth estimations taken from productivity class 3 (out of five yield classes), listed in Table 9.

Table 9: Mean growth of Maritime pine (*p. pinaster*) productivity class 3, Landes de Gascogne

| Year | Top height | Circumference |
| --- | --- | --- |
| [yr] | [m] | [cm] |
| 12 | 7.5 | 42 |
| 16 | 9.7 | 53 |
| 20 | 11.7 | 64 |
| 24 | 13.7 | 74 |
| 28 | 15.4 | 85 |
| 34 | 17.6 | 99 |
| 40 | 19.3 | 110 |
| 46 | 20.5 | 118 |
| 54 | 21.6 | 125 |
| Source: [11, p. 54] | | |

Model parameter results are illustrated in Table 10. The goodness of fit (GoF) for both age-height and age-circumference corresponds to 0.999 and 0.998 respectively. Results are plotted in Figure 10 and Figure 11, showing a comparison of the GoF from experimental yield table values and the fitted model.

Table 10: Characteristics and model fitting data of Maritime pine (*p. pinaster*)

|  | A | k | p | RSS | GoF |
| --- | --- | --- | --- | --- | --- |
| Age-height growth | 24.05081 | 0.04805 | 1.45498 | 0.3298 | 0.999 |
| Age-circumference growth | 146.1314 | 0.04069 | 1.37386 | 27.09 | 0.998 |

| 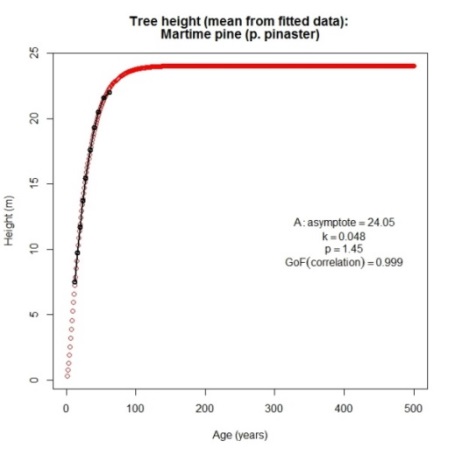  Figure 10: Age-height mean growth curve and model parameter results of Maritime pine (*p. pinaster*). Black dots represent yield table data, red circles model-predicted values | 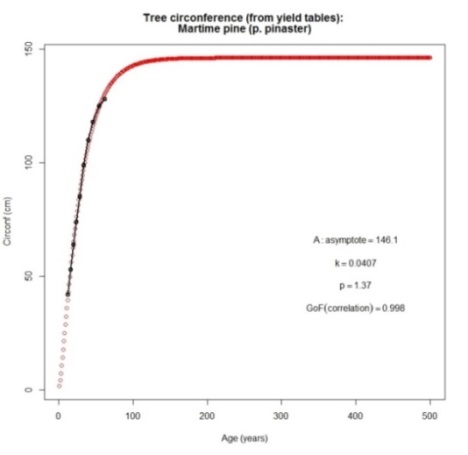  Figure 11: Age-circumference mean growth curve and model parameter results of Maritime pine (*p. pinaster*). Black dots represent yield table data, red circles model-predicted values |
| --- | --- |

Biomass growth

Cutting/thinning operations in accordance with the yield table (Table 11) to represent growth and carbon sequestration dynamics per species and year. Initial density from seedlings is 2140 stems and first thinning period at age 16 with mixed rotation cycles, it is to say four-year cycle for young stands, six-year cycle adult stands and eight-year cycle for mature stands. The dominant height is calculated from the Chapman-Richards model by non-linear regression. The total rotation length is 86 years which also represents the year of final cut (clear cut of all remaining stands). In total 9 cutting interventions are performed (including final cut).

Table 11: Forest management practices with rotation length of 70 years

|  |  | Age | Top height | Density before cut | Stems removed | Stems removed |
| --- | --- | --- | --- | --- | --- | --- |
|  |  | [yr] | [m] | [stem·ha^-1^] | [stem·ha^-1^] | [%] |
| Rotation length | 70 | - | - | - | - | - |
| Total thinning operations | 10 | - | - | - | - | - |
| Initial density | 2140 | **-** | - | - | - | - |
| Cut 1 | - | 16 | 11 | 1553 | 587 | 27% |
| Cut 2 | - | 20 | 14 | 1106 | 447 | 21% |
| Cut 3 | - | 24 | 16 | 792 | 314 | 15% |
| Cut 4 | - | 28 | 18 | 591 | 201 | 9% |
| Cut 5 | - | 34 | 20 | 436 | 155 | 7% |
| Cut 6 | - | 40 | 22 | 360 | 76 | 4% |
| Cut 7 | - | 46 | 23 | 312 | 48 | 2% |
| Cut 8 | - | 54 | 25 | 284 | 28 | 1% |
| Cut 9 | - | 62 | 26 | 272 | 12 | 1% |
| Final clear cut | - | 70 | 26 | 0 | 272 | 13% |
| Source: [11, p. 54] |  |  |  |  |  |  |

Aboveground volume estimations based on allometric equation and coefficients. Growth dynamics of individual stem volume (Figure 12) and managed stand (Figure 13), with 1.91 m^3^ and 514 m^3^·ha^-1^ respectively over 70-years rotation cycle.

| 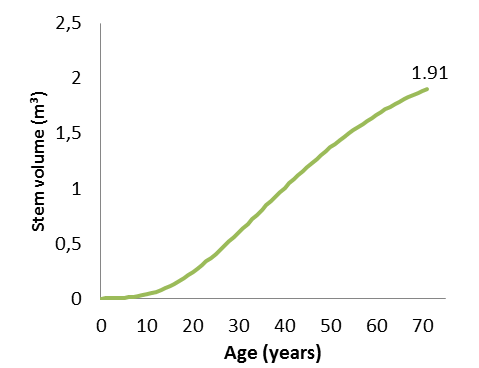 | 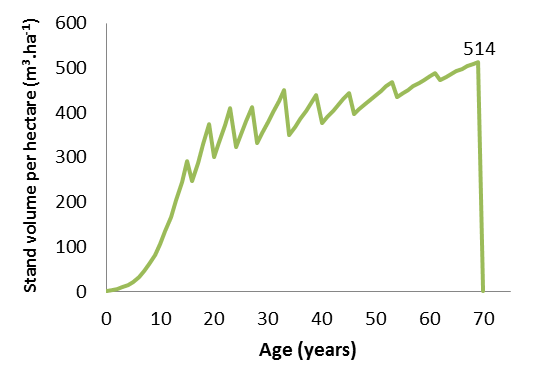 |
| --- | --- |
| Figure 12: Maritime pine (*p. pinaster*) mean aboveground individual stem volume (m^3^) over 70-year rotation cycle | Figure 13: Maritime pine (*p. pinaster*) mean aboveground volume per hectare (m^3^·ha^-1^) with thinning periods and clear cut at age 70 |

Carbon fixation

Carbon sequestration and storage of Maritime pine (*p. pinaster*) over a 70-year rotation cycle (Table 12). Stocking factors applicable for rotation cycles before harvest (historic modelling approach).

Table 12: C_bio_ stocking factors for Maritime pine (*p. pinaster*), 70-year rotation cycle

| Age  [yr] | C_bio_  growth  [t C_bio]_ | Annual C_bio_ stocking factors  [t C_bio_·yr^-1^] | Age  [yr] | C_bio_  growth  [t C_bio]_ | Annual C_bio_ stocking factors  [t C_bio_·yr^-1^] | Age  [yr] | C_bio_  growth  [t C_bio]_ | Annual C_bio_ stocking factors  [ t C_bio_·yr^-1^] |
| --- | --- | --- | --- | --- | --- | --- | --- | --- |
| 0 | 0.000000 | 0.044981 | 30 | 0.758903 | 0.008870 | 60 | 0.978993 | 0.004247 |
| 1 | 0.044981 | 0.044625 | 31 | 0.767773 | 0.008626 | 61 | 0.983240 | 0.003776 |
| 2 | 0.089606 | 0.044086 | 32 | 0.776399 | 0.008407 | 62 | 0.987016 | 0.003275 |
| 3 | 0.133692 | 0.043355 | 33 | 0.784806 | 0.008298 | 63 | 0.990291 | 0.002772 |
| 4 | 0.177047 | 0.042426 | 34 | 0.793104 | 0.008075 | 64 | 0.993062 | 0.002263 |
| 5 | 0.219472 | 0.041296 | 35 | 0.801179 | 0.008026 | 65 | 0.995325 | 0.001760 |
| 6 | 0.260768 | 0.039966 | 36 | 0.809205 | 0.007969 | 66 | 0.997085 | 0.001279 |
| 7 | 0.300735 | 0.038441 | 37 | 0.817174 | 0.007909 | 67 | 0.998364 | 0.000839 |
| 8 | 0.339176 | 0.036729 | 38 | 0.825083 | 0.007854 | 68 | 0.999204 | 0.000472 |
| 9 | 0.375904 | 0.034845 | 39 | 0.832937 | 0.007845 | 69 | 0.999676 | 0.000324 |
| 10 | 0.410749 | 0.032812 | 40 | 0.840782 | 0.007772 | 70 | 1.000000 | 0.000000 |
| 11 | 0.443561 | 0.030659 | 41 | 0.848553 | 0.007774 |  |  |  |
| 12 | 0.474221 | 0.028428 | 42 | 0.856327 | 0.007761 |  |  |  |
| 13 | 0.502649 | 0.026173 | 43 | 0.864088 | 0.007736 |  |  |  |
| 14 | 0.528822 | 0.023980 | 44 | 0.871825 | 0.007703 |  |  |  |
| 15 | 0.552802 | 0.022184 | 45 | 0.879527 | 0.007684 |  |  |  |
| 16 | 0.574985 | 0.019967 | 46 | 0.887211 | 0.007609 |  |  |  |
| 17 | 0.594952 | 0.018447 | 47 | 0.894820 | 0.007564 |  |  |  |
| 18 | 0.613399 | 0.016989 | 48 | 0.902385 | 0.007492 |  |  |  |
| 19 | 0.630388 | 0.015846 | 49 | 0.909876 | 0.007389 |  |  |  |
| 20 | 0.646235 | 0.014394 | 50 | 0.917266 | 0.007257 |  |  |  |
| 21 | 0.660629 | 0.013478 | 51 | 0.924523 | 0.007095 |  |  |  |
| 22 | 0.674107 | 0.012611 | 52 | 0.931618 | 0.006904 |  |  |  |
| 23 | 0.686718 | 0.011968 | 53 | 0.938522 | 0.006697 |  |  |  |
| 24 | 0.698686 | 0.011108 | 54 | 0.945219 | 0.006434 |  |  |  |
| 25 | 0.709794 | 0.010622 | 55 | 0.951653 | 0.006164 |  |  |  |
| 26 | 0.720416 | 0.010162 | 56 | 0.957817 | 0.005854 |  |  |  |
| 27 | 0.730577 | 0.009838 | 57 | 0.963671 | 0.005505 |  |  |  |
| 28 | 0.740416 | 0.009366 | 58 | 0.969177 | 0.005119 |  |  |  |
| 29 | 0.749782 | 0.009121 | 59 | 0.974295 | 0.004698 |  |  |  |

## Silver fir (*Abies alba*)

Regression analysis

Age-height and age-circumference mean tree growth development provided in yield tables of experimental silviculture plots by J.H. Bartet (1976) [18] at the Jura (500 to 1200 m altitude). Data for growth estimations taken from productivity class 12 (out of twelve yield classes) with reference height at 30.40 m, as listed in Table 13 .

Table 13: Mean growth of Silver fir (*a. alba*), productivity class 12, Jura

| Year | Top height | Diameter breast height | Circumference |
| --- | --- | --- | --- |
| [yr] | [m] | [cm] | [cm] |
| 43 | 12.7 | 22 | 69.1 |
| 51 | 16.8 | 30 | 94.2 |
| 59 | 20.2 | 37 | 116.2 |
| 67 | 23.0 | 44 | 138.2 |
| 79 | 26.4 | 52 | 163.4 |
| 91 | 28.9 | 58 | 182.2 |
| 103 | 30.9 | 63 | 197.9 |
| 115 | 32.5 | 67 | 210.5 |
| 127 | 34.0 | 70 | 219.9 |
| 139 | 35.4 | 72 | 226.2 |
| 151 | 36.7 | 75 | 235.6 |
| 163 | 38.0 | 77 | 241.9 |
| Source: [11, p. 112]  Note: $Circumference: Ci \left( t \right)=DBH \times\pi$ | | | |

Model parameter results are illustrated in Table 14. The goodness of fit (GoF) for both age-height and age-circumference corresponds to 0.998 and 0.999 respectively. Results are plotted in Figure 14 and Figure 15, showing a comparison of the GoF from experimental yield table values and the fitted model.

Table 14: Characteristics and model fitting data of Silver fir (*a. alba*)

|  | A | k | p | RSS | GoF |
| --- | --- | --- | --- | --- | --- |
| Age-height growth | 39.516 | 0.02229 | 2.22016 | 2.697 | 0.998 |
| Age-circumference growth | 250.14932 | 0.02571 | 3.11092 | 43.04 | 0.999 |

| 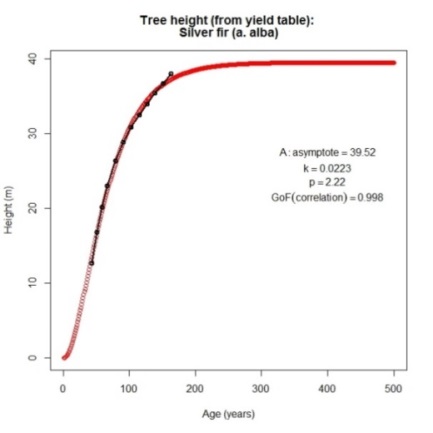 | 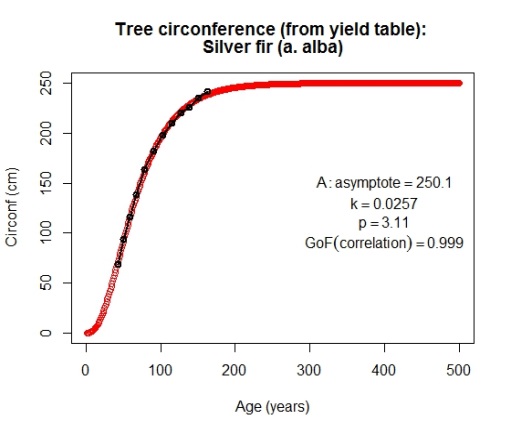 |
| --- | --- |
| Figure 14: Age-height mean growth curve and model parameter results of Silver fir (*a. alba*). Black dots represent yield table data, red circles model-predicted values | Figure 15: Age-circumference mean growth curve and model parameter results of Silver fir (*a. alba*). Black dots represent yield table data, red circles model-predicted values |

Biomass growth

Cutting/thinning operations in accordance with the yield table (Table 15) to represent growth and carbon sequestration dynamics per species and year. Initial density from seedlings is 2200 stems and first thinning period at age 43 with mixed rotation cycles, it is to say eight-year rotation for younger stands and twelve-year cycle for more mature stands. The dominant height is calculated from the Chapman-Richards model by non-linear regression. The total rotation length is 163 years which also represents the year of final cut (clear cut of all remaining stands). In total 12 cutting interventions are performed (including final cut).

Table 15: Even-aged forest stand forest management practices with rotation length of 163 years

|  |  | Age | Top height | Density before cut | Stems removed | Stems removed |
| --- | --- | --- | --- | --- | --- | --- |
|  |  | [yr] | [m] | [stem·ha^-1^] | [stem·ha^-1^] | [%] |
| Rotation length | 163 | - | - | - | - | - |
| Total thinning operations | 12 | - | - | - | - | - |
| Initial density | 2200 | - | - | - | - | - |
| Cut 1 | - | 43 | 14 | 2200 | 885 | 40% |
| Cut 2 | - | 51 | 17 | 1315 | 440 | 20% |
| Cut 3 | - | 59 | 20 | 875 | 245 | 11% |
| Cut 4 | - | 67 | 22 | 630 | 183 | 8% |
| Cut 5 | - | 79 | 26 | 447 | 100 | 5% |
| Cut 6 | - | 91 | 29 | 347 | 68 | 3% |
| Cut 7 | - | 103 | 31 | 279 | 51 | 2% |
| Cut 8 | - | 115 | 33 | 228 | 40 | 2% |
| Cut 9 | - | 127 | 35 | 189 | 32 | 1% |
| Cut 10 | - | 139 | 36 | 156 | 27 | 1% |
| Cut 11 | - | 151 | 37 | 129 | 22 | 1% |
| Final clear cut | - | 163 | 37 | 107 | 107 | 5% |
| Source: [11, p. 112] | | | | | | |

Aboveground volume estimations based on allometric equation and coefficients. Growth dynamics of individual stem volume (Figure 16) and managed stand (Figure 17), with 7.88 m^3^ and 844 m^3^·ha^-1^ respectively over 163-years rotation cycle.

| 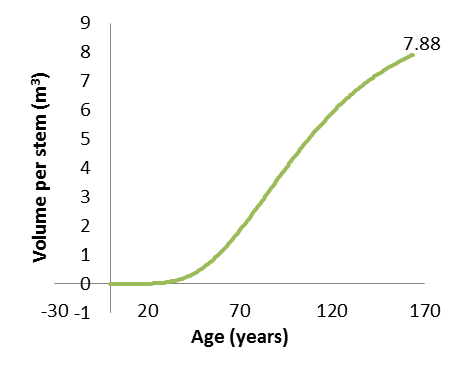 | 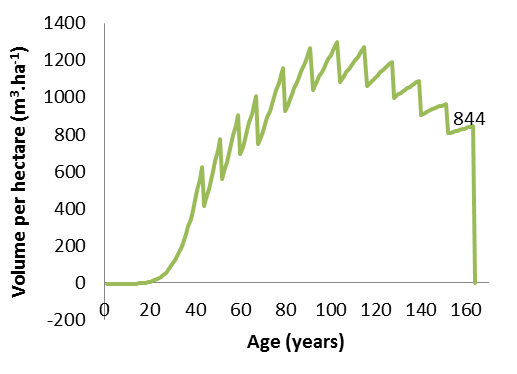 |
| --- | --- |
| Figure 16: Silver fir (*a. alba*) mean aboveground individual stem volume (m^3^) over 163-year rotation cycle | Figure 17: Silver fir (*a. alba*) mean aboveground volume per hectare (m^3^·ha^-1^) with thinning operations and clear cut at age 163 |

Carbon fixation

Carbon sequestration and storage of Silver fir (*a. alba*) over a 163-year rotation cycle Table 16. Stocking factors applicable for rotation cycles before harvest (historic modelling approach).

Table 16: C_bio_ stocking factors for Silver fir (*a. alba*), 163-year rotation cycle

| Age  [yr] | C_bio_  growth  [t C_bio]_ | Annual C_bio_ stocking factors  [t C_bio_·yr^-1^] | Age  [yr] | C_bio_  growth  [t C_bio]_ | Annual C_bio_ stocking factors  [t C_bio_·yr^-1^] | Age  [yr] | C_bio_  growth  [t C_bio]_ | Annual C_bio_ stocking factors  [t C_bio_·yr^-1^] |
| --- | --- | --- | --- | --- | --- | --- | --- | --- |
| 0 | 0.000000 | 0.017892 | 60 | 0.817600 | 0.003812 | 120 | 0.958918 | 0.001619 |
| 1 | 0.017892 | 0.018097 | 61 | 0.821411 | 0.003722 | 121 | 0.960537 | 0.001604 |
| 2 | 0.035989 | 0.018294 | 62 | 0.825134 | 0.003631 | 122 | 0.962141 | 0.001587 |
| 3 | 0.054283 | 0.018484 | 63 | 0.828765 | 0.003536 | 123 | 0.963727 | 0.001568 |
| 4 | 0.072767 | 0.018664 | 64 | 0.832301 | 0.003435 | 124 | 0.965295 | 0.001546 |
| 5 | 0.091431 | 0.018835 | 65 | 0.835736 | 0.003322 | 125 | 0.966841 | 0.001522 |
| 6 | 0.110266 | 0.018994 | 66 | 0.839058 | 0.003140 | 126 | 0.968363 | 0.001483 |
| 7 | 0.129260 | 0.019141 | 67 | 0.842198 | 0.003098 | 127 | 0.969846 | 0.001468 |
| 8 | 0.148400 | 0.019273 | 68 | 0.845296 | 0.003060 | 128 | 0.971314 | 0.001452 |
| 9 | 0.167674 | 0.019391 | 69 | 0.848356 | 0.003020 | 129 | 0.972766 | 0.001434 |
| 10 | 0.187065 | 0.019492 | 70 | 0.851376 | 0.002978 | 130 | 0.974200 | 0.001414 |
| 11 | 0.206556 | 0.019574 | 71 | 0.854354 | 0.002934 | 131 | 0.975614 | 0.001392 |
| 12 | 0.226131 | 0.019637 | 72 | 0.857289 | 0.002890 | 132 | 0.977005 | 0.001367 |
| 13 | 0.245767 | 0.019677 | 73 | 0.860178 | 0.002843 | 133 | 0.978373 | 0.001341 |
| 14 | 0.265445 | 0.019695 | 74 | 0.863022 | 0.002796 | 134 | 0.979714 | 0.001312 |
| 15 | 0.285140 | 0.019687 | 75 | 0.865817 | 0.002745 | 135 | 0.981026 | 0.001281 |
| 16 | 0.304827 | 0.019653 | 76 | 0.868562 | 0.002690 | 136 | 0.982307 | 0.001246 |
| 17 | 0.324480 | 0.019590 | 77 | 0.871253 | 0.002629 | 137 | 0.983553 | 0.001207 |
| 18 | 0.344070 | 0.019497 | 78 | 0.873882 | 0.002529 | 138 | 0.984760 | 0.001156 |
| 19 | 0.363567 | 0.019373 | 79 | 0.876411 | 0.002505 | 139 | 0.985917 | 0.001123 |
| 20 | 0.382940 | 0.019215 | 80 | 0.878916 | 0.002483 | 140 | 0.987040 | 0.001088 |
| 21 | 0.402155 | 0.019023 | 81 | 0.881400 | 0.002459 | 141 | 0.988128 | 0.001051 |
| 22 | 0.421178 | 0.018796 | 82 | 0.883859 | 0.002434 | 142 | 0.989179 | 0.001010 |
| 23 | 0.439973 | 0.018532 | 83 | 0.886293 | 0.002407 | 143 | 0.990189 | 0.000967 |
| 24 | 0.458506 | 0.018233 | 84 | 0.888700 | 0.002380 | 144 | 0.991156 | 0.000922 |
| 25 | 0.476738 | 0.017897 | 85 | 0.891080 | 0.002351 | 145 | 0.992078 | 0.000875 |
| 26 | 0.494635 | 0.017525 | 86 | 0.893432 | 0.002321 | 146 | 0.992953 | 0.000825 |
| 27 | 0.512161 | 0.017119 | 87 | 0.895753 | 0.002289 | 147 | 0.993778 | 0.000773 |
| 28 | 0.529280 | 0.016680 | 88 | 0.898042 | 0.002254 | 148 | 0.994551 | 0.000719 |
| 29 | 0.545960 | 0.016210 | 89 | 0.900295 | 0.002214 | 149 | 0.995269 | 0.000662 |
| 30 | 0.562170 | 0.015713 | 90 | 0.902509 | 0.002147 | 150 | 0.995931 | 0.000596 |
| 31 | 0.577883 | 0.015191 | 91 | 0.904656 | 0.002132 | 151 | 0.996527 | 0.000547 |
| 32 | 0.593074 | 0.014648 | 92 | 0.906788 | 0.002118 | 152 | 0.997074 | 0.000498 |
| 33 | 0.607722 | 0.014090 | 93 | 0.908907 | 0.002103 | 153 | 0.997572 | 0.000450 |
| 34 | 0.621812 | 0.013520 | 94 | 0.911010 | 0.002086 | 154 | 0.998021 | 0.000402 |
| 35 | 0.635332 | 0.012941 | 95 | 0.913096 | 0.002069 | 155 | 0.998423 | 0.000356 |
| 36 | 0.648273 | 0.012357 | 96 | 0.915165 | 0.002050 | 156 | 0.998779 | 0.000311 |
| 37 | 0.660630 | 0.011769 | 97 | 0.917215 | 0.002031 | 157 | 0.999090 | 0.000268 |
| 38 | 0.672399 | 0.011174 | 98 | 0.919245 | 0.002010 | 158 | 0.999358 | 0.000225 |
| 39 | 0.683573 | 0.010565 | 99 | 0.921256 | 0.001988 | 159 | 0.999583 | 0.000183 |
| 40 | 0.694139 | 0.009930 | 100 | 0.923243 | 0.001963 | 160 | 0.999767 | 0.000139 |
| 41 | 0.704069 | 0.009247 | 101 | 0.925206 | 0.001934 | 161 | 0.999906 | 0.000091 |
| 42 | 0.713316 | 0.008241 | 102 | 0.927140 | 0.001886 | 162 | 0.999996 | 0.000004 |
| 43 | 0.721556 | 0.007917 | 103 | 0.929026 | 0.001876 | 163 | 1.000000 | 0.000000 |
| 44 | 0.729473 | 0.007619 | 104 | 0.930902 | 0.001866 |  |  |  |
| 45 | 0.737092 | 0.007317 | 105 | 0.932768 | 0.001855 |  |  |  |
| 46 | 0.744410 | 0.007011 | 106 | 0.934623 | 0.001843 |  |  |  |
| 47 | 0.751421 | 0.006697 | 107 | 0.936467 | 0.001831 |  |  |  |
| 48 | 0.758117 | 0.006368 | 108 | 0.938297 | 0.001817 |  |  |  |
| 49 | 0.764486 | 0.006015 | 109 | 0.940114 | 0.001802 |  |  |  |
| 50 | 0.770500 | 0.005500 | 110 | 0.941916 | 0.001786 |  |  |  |
| 51 | 0.776000 | 0.005324 | 111 | 0.943702 | 0.001769 |  |  |  |
| 52 | 0.781324 | 0.005160 | 112 | 0.945471 | 0.001749 |  |  |  |
| 53 | 0.786484 | 0.004994 | 113 | 0.947219 | 0.001726 |  |  |  |
| 54 | 0.791478 | 0.004826 | 114 | 0.948945 | 0.001687 |  |  |  |
| 55 | 0.796303 | 0.004653 | 115 | 0.950632 | 0.001678 |  |  |  |
| 56 | 0.800956 | 0.004472 | 116 | 0.952310 | 0.001669 |  |  |  |
| 57 | 0.805428 | 0.004278 | 117 | 0.953979 | 0.001658 |  |  |  |
| 58 | 0.809706 | 0.003994 | 118 | 0.955637 | 0.001647 |  |  |  |
| 59 | 0.813700 | 0.003899 | 119 | 0.957284 | 0.001634 |  |  |  |

## Scots pine (*Pinus sylvestri*)

Regression analysis

Age-height and age-circumference mean tree growth development provided in yield tables of experimental silviculture plots by from Décourt (1965) [18] at Sologne. Data for growth estimations taken from productivity class 3 (out of five yield classes), as listed in Table 17.

Table 17: Mean growth of Scots pine (*p. sylvestri*), productivity class 3, Sologne

| Year | Top height | Circumference |
| --- | --- | --- |
| [yr] | [m] | [cm] |
| 25 | 10.4 | 32 |
| 30 | 12.6 | 40 |
| 35 | 14.6 | 49 |
| 40 | 16.4 | 59 |
| 50 | 19.5 | 74 |
| 60 | 22.1 | 88 |
| 70 | 24.2 | 101 |
| 80 | 25.9 | 112 |
| Source: [11, p. 20] | | |

Non-linear regression based on the Chapman-Richards curve [7]. Model parameter results are illustrated in Table 18. The goodness of fit (GoF) for both age-height and age-circumference corresponds to 1. Results are plotted in Figure 18 and Figure 19, showing a comparison of the GoF from experimental yield table values and the fitted model.

Table 18: Characteristics and model statistics of Scots pine (*p. sylvestri*)

|  | A | k | p | RSS | GoF |
| --- | --- | --- | --- | --- | --- |
| Age-height growth | 32.049 | 0.02487 | 1.455 | 0.008909 | 1 |
| Age-circumference growth | 165.24315 | 0.02043 | 1.79868 | 1.92 | 1 |

| 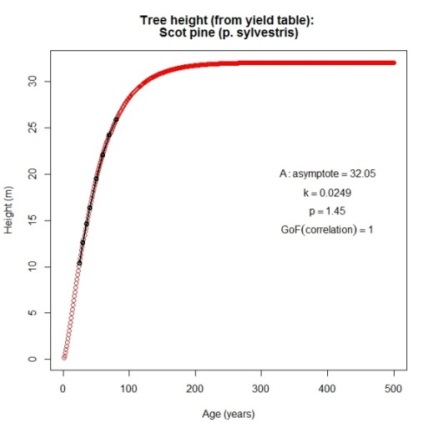 | 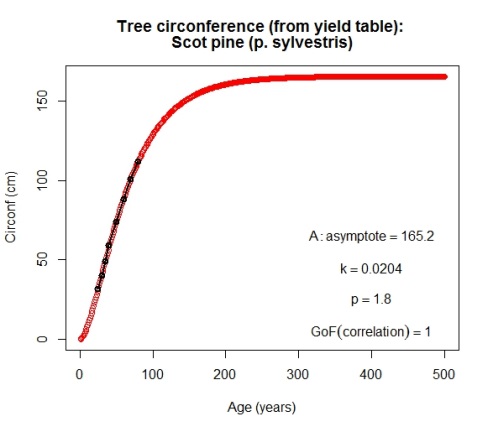 |
| --- | --- |
| Figure 18: Age-height mean growth curve and model parameter results of Scots pine (*p. sylvestri*). Black dots represent yield table data, red circles model-predicted values | Figure 19: Age-circumference mean growth curve and model parameter results of Scots pine *(p. sylvestri*). Black dots represent yield table data, red circles model-predicted values |

Biomass growth

Cutting/thinning operations in accordance with the yield table (Table 19) to represent growth and carbon sequestration dynamics per species and year. Initial density from seedlings is 3534 stems and first thinning period at age 30 with a five-year rotation cycle for followed by ten-year rotation cycles. The dominant height is calculated from the Chapman-Richards model by non-linear regression. The total rotation length is 90 years which also represents the year of final cut (clear cut of all remaining stands). In total 8 cutting interventions are performed (including final cut).

Table 19: Forest management practices with rotation length

|  |  | Age | Top height | Density before cut | Stems removed | Stems removed |
| --- | --- | --- | --- | --- | --- | --- |
|  |  | [yr] | [m] | [stem·ha^-1^] | [stem·ha^-1^] | [%] |
| Rotation length | 90 | - | - | - | - | - |
| Total thinning operations | 8 | - | - | - | - | - |
| Initial density | 3542 |  | - | - | - | - |
| Cut 1 | - | 30 | 13 | 1815 | 1727 | 48.8% |
| Cut 2 | - | 35 | 15 | 1268 | 547 | 15.4% |
| Cut 3 | - | 40 | 16 | 970 | 298 | 8.4% |
| Cut 4 | - | 50 | 20 | 662 | 308 | 8.7% |
| Cut 5 | - | 60 | 22 | 492 | 170 | 4.8% |
| Cut 6 | - | 70 | 24 | 400 | 92 | 2.6% |
| Cut 7 | - | 80 | 26 | 348 | 52 | 1.5% |
| Final clear cut | - | 90 | 27 | 0 | 348 | 10.0% |
| Source: [11, p. 20] and fitted height | | | | | | |

Aboveground volume estimations based on allometric equation and coefficients. Growth dynamics of individual stem volume (Figure 20) and managed stand (Figure 21), with 1.61 m^3^ and 594 m^3^·ha^-1^ respectively over 90-years rotation cycle.

| 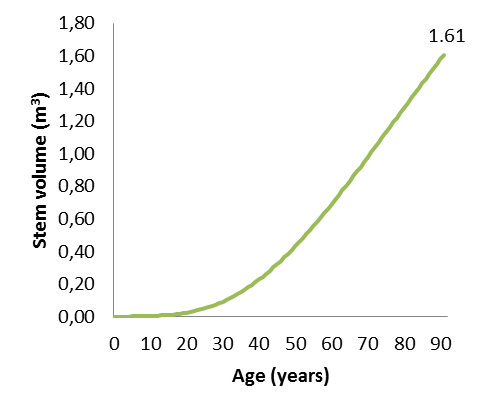 | 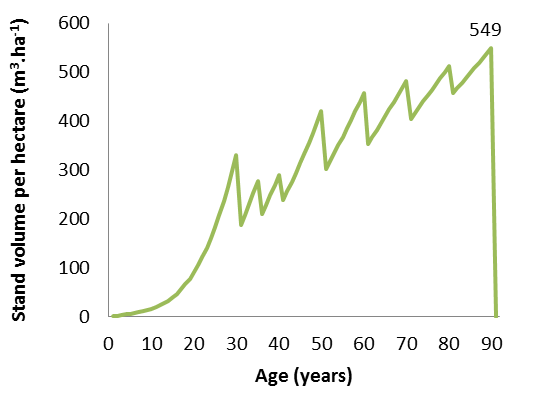 |
| --- | --- |
| Figure 20: Scots pine (*p. sylvestri*) mean aboveground individual stem volume (m^3^) over 90-year rotation cycle | Figure 21: Scots pine (*p. sylvestri*) mean aboveground individual stem volume (m^3^) over 90-year rotation cycle |

Carbon fixation

Carbon sequestration and storage of Scots pine (*p. sylvestri*), over a 90-year rotation cycle (Table 20). Stocking factors applicable for rotation cycles before harvest (historic modelling approach).

Table 20: C_bio_ stocking factors for Scots pine (*p. sylvestri*), 90-year rotation cycle

| Age  [yr] | C_bio_  growth  [t C_bio]_ | Annual C_bio_ stocking factors  [t C_bio_·yr^-1^] | Age  [yr] | C_bio_  growth  [t C_bio]_ | Annual C_bio_ stocking factors  [t C_bio_·yr^-1^] | Age  [yr] | C_bio_  growth  [t C_bio]_ | Annual C_bio_ stocking factors  [t C_bio_·yr^-1^] |
| --- | --- | --- | --- | --- | --- | --- | --- | --- |
| 0 | 0.000000 | 0.032485 | 40 | 0.819369 | 0.007223 | 80 | 0.993587 | 0.001333 |
| 1 | 0.032485 | 0.032426 | 41 | 0.826592 | 0.007054 | 81 | 0.994920 | 0.001163 |
| 2 | 0.064911 | 0.032319 | 42 | 0.833646 | 0.006877 | 82 | 0.996083 | 0.000994 |
| 3 | 0.097230 | 0.032162 | 43 | 0.840522 | 0.006694 | 83 | 0.997077 | 0.000828 |
| 4 | 0.129392 | 0.031952 | 44 | 0.847216 | 0.006508 | 84 | 0.997904 | 0.000667 |
| 5 | 0.161344 | 0.031687 | 45 | 0.853724 | 0.006320 | 85 | 0.998571 | 0.000514 |
| 6 | 0.193031 | 0.031367 | 46 | 0.860044 | 0.006134 | 86 | 0.999085 | 0.000373 |
| 7 | 0.224398 | 0.030988 | 47 | 0.866178 | 0.005955 | 87 | 0.999458 | 0.000249 |
| 8 | 0.255386 | 0.030550 | 48 | 0.872133 | 0.005791 | 88 | 0.999708 | 0.000152 |
| 9 | 0.285936 | 0.030051 | 49 | 0.877924 | 0.005695 | 89 | 0.999859 | 0.000141 |
| 10 | 0.315987 | 0.029491 | 50 | 0.883618 | 0.005476 | 90 | 1.000000 | 0.000000 |
| 11 | 0.345478 | 0.028869 | 51 | 0.889094 | 0.005374 |  |  |  |
| 12 | 0.374346 | 0.028184 | 52 | 0.894468 | 0.005266 |  |  |  |
| 13 | 0.402531 | 0.027438 | 53 | 0.899734 | 0.005151 |  |  |  |
| 14 | 0.429969 | 0.026631 | 54 | 0.904885 | 0.005031 |  |  |  |
| 15 | 0.456600 | 0.025766 | 55 | 0.909916 | 0.004907 |  |  |  |
| 16 | 0.482366 | 0.024844 | 56 | 0.914822 | 0.004780 |  |  |  |
| 17 | 0.507210 | 0.023868 | 57 | 0.919602 | 0.004654 |  |  |  |
| 18 | 0.531078 | 0.022844 | 58 | 0.924256 | 0.004532 |  |  |  |
| 19 | 0.553922 | 0.021776 | 59 | 0.928788 | 0.004443 |  |  |  |
| 20 | 0.575698 | 0.020670 | 60 | 0.933231 | 0.004283 |  |  |  |
| 21 | 0.596368 | 0.019535 | 61 | 0.937515 | 0.004184 |  |  |  |
| 22 | 0.615903 | 0.018379 | 62 | 0.941699 | 0.004076 |  |  |  |
| 23 | 0.634282 | 0.017215 | 63 | 0.945775 | 0.003961 |  |  |  |
| 24 | 0.651496 | 0.016055 | 64 | 0.949736 | 0.003838 |  |  |  |
| 25 | 0.667551 | 0.014916 | 65 | 0.953575 | 0.003709 |  |  |  |
| 26 | 0.682467 | 0.013820 | 66 | 0.957284 | 0.003574 |  |  |  |
| 27 | 0.696287 | 0.012794 | 67 | 0.960858 | 0.003435 |  |  |  |
| 28 | 0.709081 | 0.011886 | 68 | 0.964293 | 0.003293 |  |  |  |
| 29 | 0.720967 | 0.011403 | 69 | 0.967586 | 0.003166 |  |  |  |
| 30 | 0.732369 | 0.010276 | 70 | 0.970752 | 0.002996 |  |  |  |
| 31 | 0.742645 | 0.009852 | 71 | 0.973748 | 0.002855 |  |  |  |
| 32 | 0.752497 | 0.009444 | 72 | 0.976603 | 0.002706 |  |  |  |
| 33 | 0.761941 | 0.009067 | 73 | 0.979309 | 0.002549 |  |  |  |
| 34 | 0.771008 | 0.008819 | 74 | 0.981858 | 0.002386 |  |  |  |
| 35 | 0.779827 | 0.008362 | 75 | 0.984244 | 0.002217 |  |  |  |
| 36 | 0.788189 | 0.008124 | 76 | 0.986462 | 0.002044 |  |  |  |
| 37 | 0.796312 | 0.007888 | 77 | 0.988506 | 0.001868 |  |  |  |
| 38 | 0.804201 | 0.007664 | 78 | 0.990374 | 0.001691 |  |  |  |
| 39 | 0.811865 | 0.007504 | 79 | 0.992064 | 0.001523 |  |  |  |

## Other conifers (*Pinaceae spp*)

Regression analysis

Mean tree growth development based on rotation cycles (here age) from yield table of Norway spruce, productivity class 16 of experimental silviculture plot by P. Deplat and R. Bolliet (1979) (available in INRA/ONF/ENGREF 1984) at the South of Massif Central (Montagne Noire, Monts de Lacune-Sommail-Espinouse, Levezou and Aigoual). Mean height and circumference values from five fitted non-linear regression data of the five assessed conifer trees (Douglas fir, Norway spruce, Maritime pine, Silver fir and Scots pine) are shown in Table 21.

Table 21: Mean growth based on five conifers and fitted data

| Year | Top height | Circumference |
| --- | --- | --- |
| [yr] | [m] | [cm] |
| 30 | 14.7 | 61.0 |
| 37 | 17.9 | 75.1 |
| 44 | 20.7 | 88.3 |
| 51 | 23.2 | 100.2 |
| 58 | 25.3 | 111.0 |
| 65 | 27.2 | 120.7 |
| 72 | 28.8 | 129.3 |
| 79 | 30.1 | 137.0 |
| 86 | 31.3 | 143.7 |
| Source: adapted from [11, p. 134] and fitted data | | |

Model parameter results are illustrated in Table 22. The goodness of fit (GoF) for both age-height and age-circumference corresponds to 1. Results are plotted in Figure 22 and Figure 23.

Table 22: Characteristics and model fitting data for other conifer species

|  | A | k | p | RSS | GoF |
| --- | --- | --- | --- | --- | --- |
| Age-height growth | 37.87903 | 0.02422 | 1.43118 | 0.0001438 | 1 |
| Age-circumference growth | 192.55739 | 0.01951 | 1.41388 | 0.009865 | 1 |

| 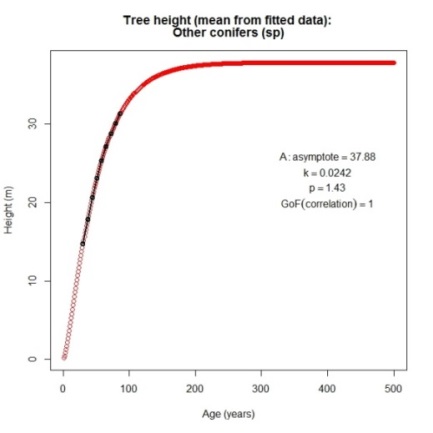  Figure 22: Age-height mean growth curve and model parameter results for other conifer species. Black dots represent yield table data, red circles model-predicted values | 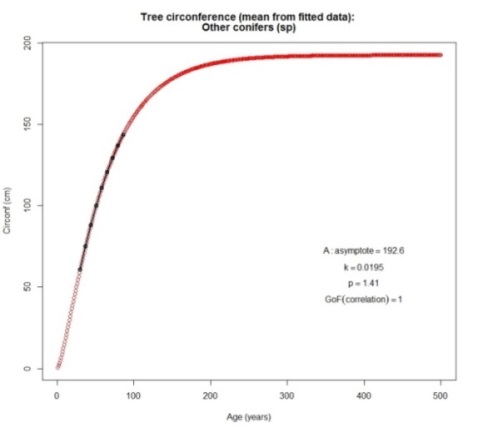  Figure 23: Age-circumference mean growth curve and model parameter results for other conifer species. Black dots represent yield table data, red circles model-predicted values |
| --- | --- |

Biomass growth

Same as Norway spruce (*p. abies*) with age-height growth adapted to Scots pine total aboveground volume coefficients. Aboveground volume estimations based on allometric equation and coefficients. Growth dynamics of individual stem volume (Figure 24) and managed stand (Figure 25), with 2.59 m^3^ and 588 m^3^·ha^-1^ respectively over 86-years rotation cycle.

| 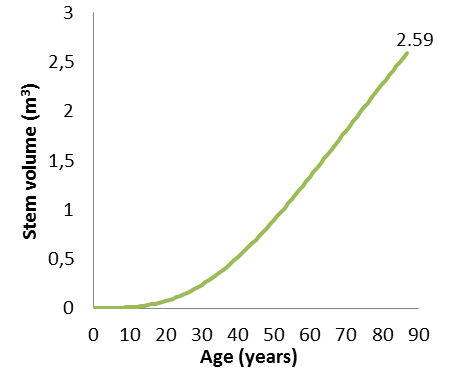 | 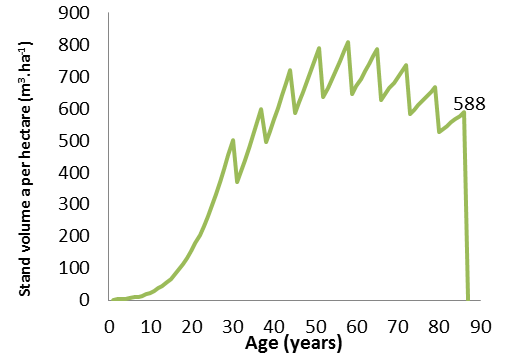 |
| --- | --- |
| Figure 24: Other conifers mean aboveground individual stem volume (m^3^) over 86-year rotation cycle | Figure 25: Other conifers mean aboveground individual stem volume (m^3^) over 86-year rotation cycle |

Carbon fixation

Carbon sequestration and storage of other conifers over a 86-year rotation cycle (Table 23). Stocking factors applicable for rotation cycles before harvest (historic modelling approach).

Table 23: C_bio_ stocking factors for other conifers, 86-year rotation cycle

| Age  [yr] | C_bio_  growth  [t C_bio]_ | Annual C_bio_ stocking factors  [t C_bio_·yr^-1^] | Age  [yr] | C_bio_  growth  [t C_bio]_ | Annual C_bio_ stocking factors  [tC_bio_·yr^-1^] | Age  [yr] | C_bio_  growth  [t C_bio]_ | Annual C_bio_ stocking factors  [t C_bio_·yr^-1^] |
| --- | --- | --- | --- | --- | --- | --- | --- | --- |
| 0 | 0.000000 | 0.029257 | 40 | 0.817265 | 0.009794 | 80 | 0.998652 | 0.000431 |
| 1 | 0.029257 | 0.029118 | 41 | 0.827059 | 0.009438 | 81 | 0.999083 | 0.000328 |
| 2 | 0.058375 | 0.028948 | 42 | 0.836497 | 0.009093 | 82 | 0.999411 | 0.000235 |
| 3 | 0.087324 | 0.028748 | 43 | 0.845590 | 0.008807 | 83 | 0.999646 | 0.000157 |
| 4 | 0.116071 | 0.028515 | 44 | 0.854397 | 0.008405 | 84 | 0.999803 | 0.000098 |
| 5 | 0.144586 | 0.028250 | 45 | 0.862802 | 0.008107 | 85 | 0.999901 | 0.000099 |
| 6 | 0.172836 | 0.027952 | 46 | 0.870909 | 0.007807 | 86 | 1.000000 | 0.000000 |
| 7 | 0.200788 | 0.027621 | 47 | 0.878716 | 0.007506 |  |  |  |
| 8 | 0.228409 | 0.027258 | 48 | 0.886223 | 0.007210 |  |  |  |
| 9 | 0.255667 | 0.026861 | 49 | 0.893432 | 0.006922 |  |  |  |
| 10 | 0.282527 | 0.026432 | 50 | 0.900354 | 0.006678 |  |  |  |
| 11 | 0.308959 | 0.025970 | 51 | 0.907032 | 0.006344 |  |  |  |
| 12 | 0.334929 | 0.025477 | 52 | 0.913375 | 0.006089 |  |  |  |
| 13 | 0.360406 | 0.024953 | 53 | 0.919464 | 0.005832 |  |  |  |
| 14 | 0.385359 | 0.024400 | 54 | 0.925296 | 0.005574 |  |  |  |
| 15 | 0.409759 | 0.023819 | 55 | 0.930870 | 0.005318 |  |  |  |
| 16 | 0.433578 | 0.023212 | 56 | 0.936188 | 0.005069 |  |  |  |
| 17 | 0.456790 | 0.022581 | 57 | 0.941257 | 0.004852 |  |  |  |
| 18 | 0.479370 | 0.021928 | 58 | 0.946109 | 0.004567 |  |  |  |
| 19 | 0.501298 | 0.021256 | 59 | 0.950676 | 0.004341 |  |  |  |
| 20 | 0.522554 | 0.020570 | 60 | 0.955017 | 0.004114 |  |  |  |
| 21 | 0.543124 | 0.019871 | 61 | 0.959131 | 0.003886 |  |  |  |
| 22 | 0.562995 | 0.019166 | 62 | 0.963017 | 0.003660 |  |  |  |
| 23 | 0.582161 | 0.018458 | 63 | 0.966677 | 0.003438 |  |  |  |
| 24 | 0.600619 | 0.017753 | 64 | 0.970115 | 0.003243 |  |  |  |
| 25 | 0.618372 | 0.017059 | 65 | 0.973358 | 0.002994 |  |  |  |
| 26 | 0.635431 | 0.016381 | 66 | 0.976353 | 0.002793 |  |  |  |
| 27 | 0.651813 | 0.015732 | 67 | 0.979146 | 0.002592 |  |  |  |
| 28 | 0.667544 | 0.015127 | 68 | 0.981737 | 0.002391 |  |  |  |
| 29 | 0.682672 | 0.014692 | 69 | 0.984128 | 0.002192 |  |  |  |
| 30 | 0.697364 | 0.013950 | 70 | 0.986320 | 0.002000 |  |  |  |
| 31 | 0.711314 | 0.013498 | 71 | 0.988320 | 0.001830 |  |  |  |
| 32 | 0.724812 | 0.013045 | 72 | 0.990150 | 0.001621 |  |  |  |
| 33 | 0.737856 | 0.012594 | 73 | 0.991771 | 0.001451 |  |  |  |
| 34 | 0.750451 | 0.012152 | 74 | 0.993222 | 0.001285 |  |  |  |
| 35 | 0.762603 | 0.011727 | 75 | 0.994507 | 0.001122 |  |  |  |
| 36 | 0.774330 | 0.011378 | 76 | 0.995629 | 0.000966 |  |  |  |
| 37 | 0.785708 | 0.010881 | 77 | 0.996596 | 0.000819 |  |  |  |
| 38 | 0.796590 | 0.010520 | 78 | 0.997414 | 0.000693 |  |  |  |
| 39 | 0.807109 | 0.010156 | 79 | 0.998108 | 0.000544 |  |  |  |

## Chestnut (*Castanea sativa*)

Regression analysis

Age-height and age-circumference mean tree growth development provided in yield tables of experimental silviculture plots with high stand density (i.e. number of trees per hectare) and a dominant height of 16 m at a reference age of 20 years (site index) at the North of Spain [19]. Data for growth estimations taken from productivity class 4 (out of eight yield classes), as shown in Table 24.

Table 24: Mean growth of Chestnut (*c. sativa*), productivity class 4, North of Spain

| Year | Top height | Diameter breast height | Circumference |
| --- | --- | --- | --- |
| [yr] | [m] | [cm] | [cm] |
| 10 | 11 | 9 | 28.27 |
| 15 | 14.9 | 12.8 | 40.21 |
| 20 | 18 | 16.1 | 50.58 |
| 25 | 20.5 | 19.1 | 60.00 |
| 30 | 22.5 | 21.8 | 68.49 |
| 35 | 24.2 | 24.2 | 76.03 |
| 40 | 25.7 | 26.5 | 83.25 |
| 45 | 26.9 | 28.6 | 89.85 |
| 50 | 28.6 | 30.5 | 95.82 |
| 55 | 28.9 | 32.3 | 101.47 |
| 60 | 29.7 | 34 | 106.81 |
| Source: [13, p. 131]  $Circumference: Ci \left( t \right)=DBH \times\pi$ | | | |

Model parameter results are illustrated in Table 25. The goodness of fit (GoF) for both age-height and age-circumference corresponds to 1. Results are plotted in Figure 26 and Figure 27, showing a comparison of the GoF from experimental yield table values and the fitted model.

Table 25: Characteristics and model fitting data of Chestnut (*c. sativa*)

|  | A | k | p | RSS | GoF |
| --- | --- | --- | --- | --- | --- |
| Age-height growth | 33.88159 | 0.03317 | 0.88211 | 0.3351 | 1 |
| Age-circumference growth | 166.70416 | 0.01591 | 0.91983 | 0.312 | 1 |

| 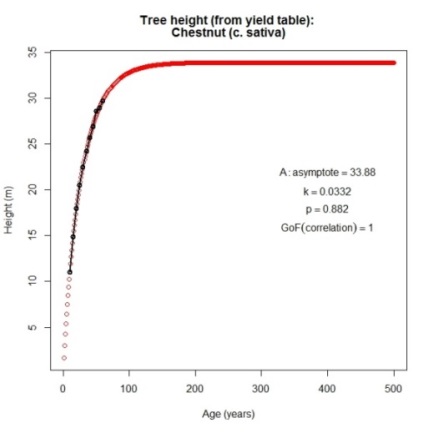 | 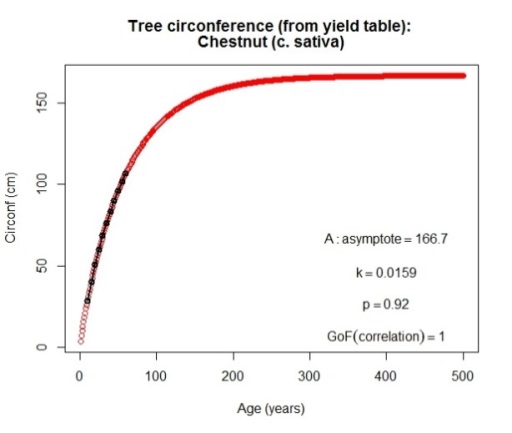 |
| --- | --- |
| Figure 26: Age-height mean growth curve and model parameter results of Chestnut (*c. sativa*). Black dots represent yield table data, red circles model-predicted values | Figure 27: Age-circumference mean growth curve and model parameter results of Chestnut (*c. sativa*). Black dots represent yield table data, red circles model-predicted values |

Biomass growth

Cutting/thinning operations in accordance with the yield table (Table 26) to represent growth and carbon sequestration dynamics per species and year. Initial density from seedlings is 3366 stems and first thinning period at age 10 with five-year rotation cycles. The dominant height is calculated from the Chapman-Richards model by non-linear regression. The total rotation length is 86 years which also represents the year of final cut (clear cut of all remaining stands). In total 11 cutting interventions are performed (including final cut).

Table 26: Chestnut even-aged forest stand forest management practices with rotation length of 60 years

|  |  | Age | Top height | Density before cut | Stems removed | Stems removed |
| --- | --- | --- | --- | --- | --- | --- |
|  |  | [yr] | [m] | [stem·ha^-1^] | [stem·ha^-1^] | [%] |
| Rotation length | 60 | - | - | - | - | - |
|  | 11 | - | - | - | - | - |
| Initial density | 3366 | - | - | - | - | - |
| Cut 1 | - | 10 | 11 | 2170 | 1196.0 | 55% |
| Cut 2 | - | 15 | 15 | 1589 | 581.0 | 27% |
| Cut 3 | - | 20 | 18 | 1248 | 341.0 | 16% |
| Cut 4 | - | 25 | 20 | 1024 | 224.0 | 10% |
| Cut 5 | - | 30 | 23 | 866 | 158.0 | 7% |
| Cut 6 | - | 35 | 24 | 749 | 117 | 5% |
| Cut 7 | - | 40 | 26 | 659 | 90 | 4% |
| Cut 8 | - | 45 | 27 | 590 | 69 | 3% |
| Cut 9 | - | 50 | 28 | 532 | 58 | 3% |
| Cut 10 | - | 55 | 29 | 484 | 48 | 2% |
| Final clear cut | - | 60 | 30 | 0 | 484 | 22% |
| Source: [13, p. 131] and fitted data | | | | | | |

Aboveground volume estimations based on allometric equation and coefficients. Growth dynamics of individual stem volume (Figure 28) and managed stand (Figure 29), with 1.36 m^3^ and 645 m^3^·ha^-1^ respectively over 60-years rotation cycle.

| 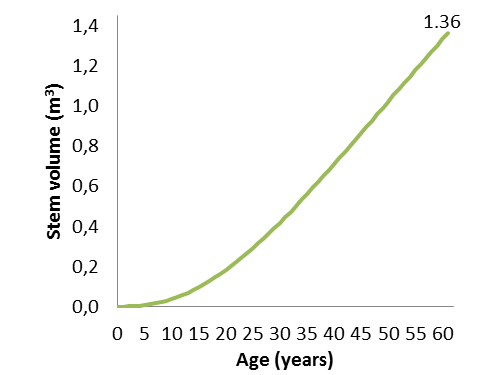 | 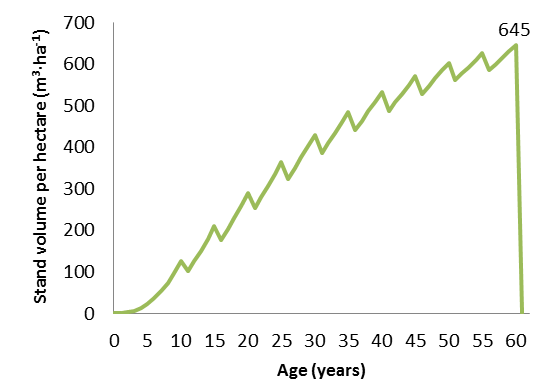 |
| --- | --- |
| Figure 28: Chestnut (*c. sativa*) mean aboveground individual stem volume (m^3^) over 60-year rotation cycle | Figure 29: Chestnut (*c. sativa*) mean aboveground volume per hectare (m^3^·ha^-1^) with thinning operations and clear cut at age 60 |

Carbon fixation

Carbon sequestration and storage of Chestnut (*c. sativa*) over a 60-year rotation cycle (Table 27). Stocking factors applicable for rotation cycles before harvest (historic modelling approach).

Table 27: C_bio_ stocking factors for Chestnut (*c. sativa*), 60-year rotation cycle

| Age  [yr] | C_bio_  growth  [t C_bio]_ | Annual C_bio_ stocking factors  [t C_bio_·yr^-1^] | Age  [yr] | C_bio_  growth  [t C_bio]_ | Annual C_bio_ stocking factors  [t C_bio_·yr^-1^] | Age  [yr] | C_bio_  growth  [t C_bio]_ | Annual C_bio_ stocking factors  [t C_bio_·yr^-1^] |
| --- | --- | --- | --- | --- | --- | --- | --- | --- |
| 0 | 0.000000 | 0.049109 | 21 | 0.669772 | 0.019538 | 42 | 0.948269 | 0.006849 |
| 1 | 0.049109 | 0.046894 | 22 | 0.689310 | 0.018824 | 43 | 0.955117 | 0.006299 |
| 2 | 0.096004 | 0.044689 | 23 | 0.708134 | 0.018126 | 44 | 0.961416 | 0.005819 |
| 3 | 0.140693 | 0.042509 | 24 | 0.726260 | 0.017652 | 45 | 0.967235 | 0.005229 |
| 4 | 0.183202 | 0.040373 | 25 | 0.743913 | 0.016812 | 46 | 0.972464 | 0.004707 |
| 5 | 0.223575 | 0.038302 | 26 | 0.760725 | 0.016177 | 47 | 0.977170 | 0.004192 |
| 6 | 0.261877 | 0.036318 | 27 | 0.776902 | 0.015544 | 48 | 0.981362 | 0.003689 |
| 7 | 0.298195 | 0.034449 | 28 | 0.792446 | 0.014919 | 49 | 0.985052 | 0.003255 |
| 8 | 0.332644 | 0.032730 | 29 | 0.807365 | 0.014448 | 50 | 0.988307 | 0.002740 |
| 9 | 0.365375 | 0.032263 | 30 | 0.821813 | 0.013715 | 51 | 0.991046 | 0.002295 |
| 10 | 0.397638 | 0.029892 | 31 | 0.835528 | 0.013124 | 52 | 0.993341 | 0.001871 |
| 11 | 0.427529 | 0.028660 | 32 | 0.848652 | 0.012531 | 53 | 0.995212 | 0.001475 |
| 12 | 0.456190 | 0.027477 | 33 | 0.861183 | 0.011941 | 54 | 0.996687 | 0.001156 |
| 13 | 0.483667 | 0.026360 | 34 | 0.873124 | 0.011463 | 55 | 0.997844 | 0.000793 |
| 14 | 0.510027 | 0.025845 | 35 | 0.884588 | 0.010790 | 56 | 0.998637 | 0.000515 |
| 15 | 0.535872 | 0.024400 | 36 | 0.895377 | 0.010219 | 57 | 0.999151 | 0.000285 |
| 16 | 0.560272 | 0.023506 | 37 | 0.905596 | 0.009647 | 58 | 0.999437 | 0.000116 |
| 17 | 0.583778 | 0.022634 | 38 | 0.915243 | 0.009076 | 59 | 0.999552 | 0.000448 |
| 18 | 0.606412 | 0.021795 | 39 | 0.924319 | 0.008591 | 60 | 1.000000 | 0.000000 |
| 19 | 0.628207 | 0.021304 | 40 | 0.932910 | 0.007956 |  |  |  |
| 20 | 0.649511 | 0.020261 | 41 | 0.940866 | 0.007402 |  |  |  |

## Hornbeam (*Carpinus betulu*s)

Regression analysis

Age-height and age-circumference mean tree growth development provided in yield tables of experimental silviculture plots of full stocked hornbeam stands of vegetative origin in the European part (ecoregions of zones of deciduous forests and forest steppe) [20] . Data for growth estimations taken from productivity class 2 (out of four yield classes), listed in Table 28.

Table 28: Mean growth of Hornbeam stands (*c. betulus*), productivity class 2, Europe

| Year | Top height | Diameter breast height | Circumference |
| --- | --- | --- | --- |
| [yr] | [m] | [cm] | [cm] |
| 30 | 15.0 | 12.4 | 38.96 |
| 35 | 16.6 | 14.1 | 44.30 |
| 40 | 18.0 | 15.6 | 49.01 |
| 45 | 19.2 | 17.1 | 53.72 |
| 50 | 20.2 | 18.3 | 57.49 |
| 55 | 21.1 | 19.5 | 61.26 |
| 60 | 21.8 | 20.5 | 64.40 |
| 65 | 22.4 | 21.5 | 67.54 |
| 70 | 23.0 | 22.4 | 70.37 |
| 75 | 23.5 | 23.1 | 72.57 |
| 80 | 23.9 | 23.8 | 74.77 |
| 85 | 24.2 | 24.5 | 76.97 |
| 90 | 24.5 | 25.0 | 78.54 |
| 95 | 24.8 | 25.5 | 80.11 |
| 100 | 25.0 | 26.0 | 81.68 |
| Source: [14, p. 375] | | | |

Non-linear regression based on the Chapman-Richards curve [7]. Model parameter results are illustrated in Table 29. The goodness of fit (GoF) for both age-height and age-circumference corresponds to 1. Results are plotted in Figure 30 and Figure 31, showing a comparison of the GoF from experimental yield table values and the fitted model.

Table 29: Characteristics and model fitting data of Hornbeam (*c. betulus*)

|  | A | k | p | RSS | GoF |
| --- | --- | --- | --- | --- | --- |
| Age-height growth | 26.2398 | 0.03175 | 1.1452 | 0.00906 | 1 |
| Age-circumference growth | 94.29 | 0.0218 | 1.2066 | 0.1398 | 1 |

| 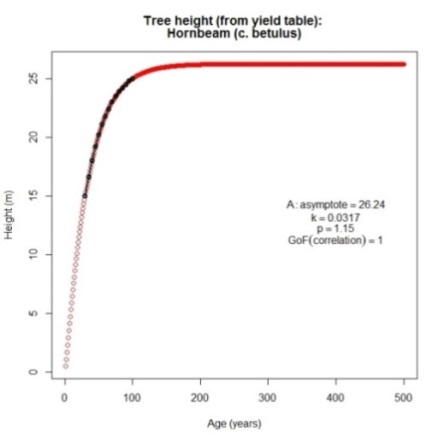 | 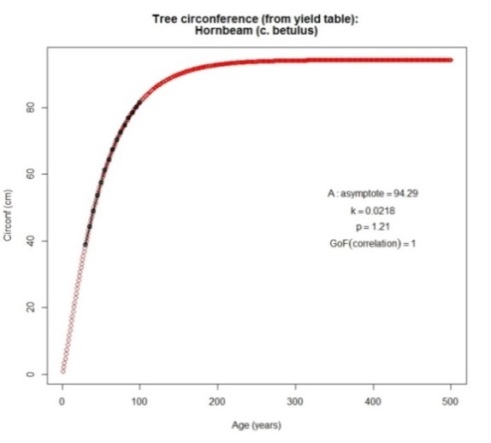 |
| --- | --- |
| Figure 30: Age-height mean growth curve and model parameter results. Black dots represent yield table data, red circles model-predicted values | Figure 31: Age-circumference mean growth curve and model parameter results. Black dots represent yield table data, red circles model-predicted values |

Carbon fixation for rotation cycles before harvest

Cutting/thinning operations in accordance with the yield table (Table 30) to represent growth and carbon sequestration dynamics per species and year. Initial density from seedlings is 1973 stems and first thinning period at age 30 with five-year rotation cycles. The dominant height is calculated from the Chapman-Richards model by non-linear regression. The total rotation length is 86 years which also represents the year of final cut (clear cut of all remaining stands). In total 15 cutting interventions are performed (including final cut).

Table 30: Hornbeam even-aged forest stand forest management practices with rotation length of 100 years

|  |  | Age | Top height | Density before cut | Stems removed | Stems removed |
| --- | --- | --- | --- | --- | --- | --- |
|  |  | [yr] | [m] | [stem·ha^-1^] | [stem·ha^-1^] | [%] |
| Rotation length | 100 | - | - | - | - | - |
| Total thinning operations | 15 | - | - | - | - | - |
| Initial density | 1973 | - | - |  | - | - |
| Cut 1 | - | 30 | 15 | 1659 | 314 | 19% |
| Cut 2 | - | 35 | 17 | 1438 | 221 | 13% |
| Cut 3 | - | 40 | 18 | 1276 | 162 | 10% |
| Cut 4 | - | 45 | 19 | 1152 | 124 | 7% |
| Cut 5 | - | 50 | 20 | 1055 | 97 | 6% |
| Cut 6 | - | 55 | 21 | 978 | 77 | 5% |
| Cut 7 | - | 60 | 22 | 915 | 63 | 4% |
| Cut 8 | - | 65 | 22 | 864 | 51 | 3% |
| Cut 9 | - | 70 | 23 | 821 | 43 | 3% |
| Cut 10 | - | 75 | 23 | 785 | 36 | 2% |
| Cut 11 | - | 80 | 24 | 754 | 31 | 2% |
| Cut 12 | - | 85 | 24 | 728 | 26 | 2% |
| Cut 13 | - | 90 | 25 | 705 | 23 | 1% |
| Cut 14 | - | 95 | 25 | 685 | 20 | 1% |
| Final clear cut | - | 100 | 25 |  | 685 | 41% |
| Source: [14, p. 375] and fitted data | | | | | | |

Aboveground volume estimations based on allometric equation and coefficients. Growth dynamics of individual stem volume (Figure 32) and managed stand (Figure 33), with 2.15 m^3^ and 1447 m^3^·ha^-1^ respectively over 86-years rotation cycle.

| 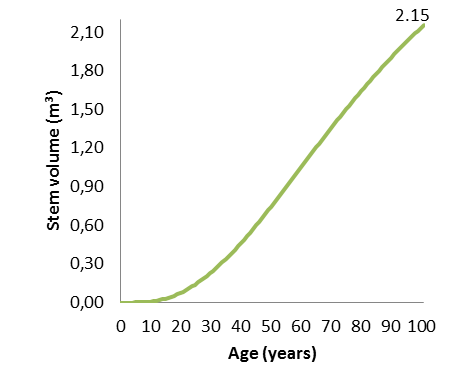 | 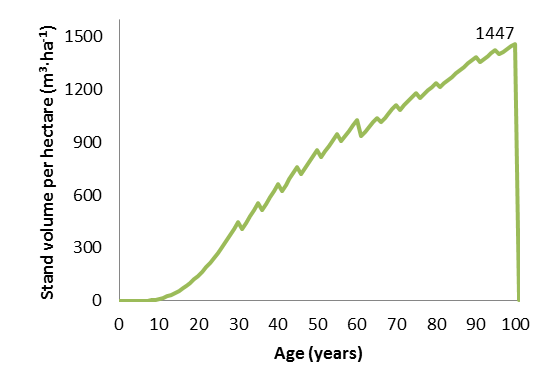 |
| --- | --- |
| Figure 32: Hornbeam (*c. betulus*) mean aboveground individual stem volume (m^3^) over 100-year rotation cycle | Figure 33: Hornbeam (*c. be*tulus) mean aboveground volume per hectare (m^3^·ha^-1^) with thinning operations and clear cut at age 100 |

Carbon fixation

Carbon sequestration and storage of Hornbeam (*c. betulus*) over a 100-year rotation cycle (Table 31). Stocking factors applicable for rotation cycles before harvest (historic modelling approach).

Table 31: C_bio_ stocking factors for Hornbeam (*c. betulus*), 100-year rotation cycle

| Age  [yr] | C_bio_  growth  [t C_bio]_ | Annual C_bio_ stocking factors  [t C_bio_·yr^-1^] | Age  [yr] | C_bio_  growth  [t C_bio]_ | Annual C_bio_ stocking factors  [t C_bio_·yr^-1^] | Age  [yr] | C_bio_  growth  [t C_bio]_ | Annual C_bio_ stocking factors  [t C_bio_·yr^-1^] |
| --- | --- | --- | --- | --- | --- | --- | --- | --- |
| 0 | 0.000000 | 0.017077 | 40 | 0.582340 | 0.010966 | 80 | 0.951402 | 0.005589 |
| 1 | 0.017077 | 0.017126 | 41 | 0.593307 | 0.010889 | 81 | 0.956991 | 0.005260 |
| 2 | 0.034203 | 0.017159 | 42 | 0.604196 | 0.010815 | 82 | 0.962252 | 0.004921 |
| 3 | 0.051362 | 0.017176 | 43 | 0.615010 | 0.010744 | 83 | 0.967173 | 0.004573 |
| 4 | 0.068538 | 0.017177 | 44 | 0.625755 | 0.010727 | 84 | 0.971746 | 0.004228 |
| 5 | 0.085716 | 0.017161 | 45 | 0.636482 | 0.010621 | 85 | 0.975974 | 0.003857 |
| 6 | 0.102877 | 0.017128 | 46 | 0.647103 | 0.010562 | 86 | 0.979831 | 0.003492 |
| 7 | 0.120005 | 0.017076 | 47 | 0.657665 | 0.010503 | 87 | 0.983324 | 0.003125 |
| 8 | 0.137081 | 0.017006 | 48 | 0.668168 | 0.010444 | 88 | 0.986449 | 0.002759 |
| 9 | 0.154088 | 0.016918 | 49 | 0.678612 | 0.010425 | 89 | 0.989208 | 0.002406 |
| 10 | 0.171006 | 0.016810 | 50 | 0.689037 | 0.010334 | 90 | 0.991614 | 0.002044 |
| 11 | 0.187816 | 0.016684 | 51 | 0.699371 | 0.010277 | 91 | 0.993658 | 0.001702 |
| 12 | 0.204500 | 0.016539 | 52 | 0.709648 | 0.010216 | 92 | 0.995360 | 0.001376 |
| 13 | 0.221039 | 0.016375 | 53 | 0.719865 | 0.010154 | 93 | 0.996736 | 0.001070 |
| 14 | 0.237413 | 0.016192 | 54 | 0.730018 | 0.010119 | 94 | 0.997807 | 0.000800 |
| 15 | 0.253605 | 0.015992 | 55 | 0.740138 | 0.010024 | 95 | 0.998606 | 0.000546 |
| 16 | 0.269598 | 0.015775 | 56 | 0.750162 | 0.009953 | 96 | 0.999152 | 0.000338 |
| 17 | 0.285373 | 0.015543 | 57 | 0.760116 | 0.009876 | 97 | 0.999491 | 0.000175 |
| 18 | 0.300916 | 0.015295 | 58 | 0.769992 | 0.009794 | 98 | 0.999666 | 0.000064 |
| 19 | 0.316211 | 0.015036 | 59 | 0.779786 | 0.009731 | 99 | 0.999730 | 0.000270 |
| 20 | 0.331247 | 0.014765 | 60 | 0.789517 | 0.009615 | 100 | 1.000000 | 0.000000 |
| 21 | 0.346011 | 0.014486 | 61 | 0.799132 | 0.009514 |  |  |  |
| 22 | 0.360497 | 0.014201 | 62 | 0.808646 | 0.009405 |  |  |  |
| 23 | 0.374699 | 0.013915 | 63 | 0.818051 | 0.009287 |  |  |  |
| 24 | 0.388613 | 0.013629 | 64 | 0.827339 | 0.009181 |  |  |  |
| 25 | 0.402243 | 0.013350 | 65 | 0.836520 | 0.009028 |  |  |  |
| 26 | 0.415592 | 0.013080 | 66 | 0.845548 | 0.008883 |  |  |  |
| 27 | 0.428672 | 0.012826 | 67 | 0.854431 | 0.008727 |  |  |  |
| 28 | 0.441499 | 0.012593 | 68 | 0.863157 | 0.008559 |  |  |  |
| 29 | 0.454092 | 0.012505 | 69 | 0.871716 | 0.008398 |  |  |  |
| 30 | 0.466597 | 0.012201 | 70 | 0.880114 | 0.008192 |  |  |  |
| 31 | 0.478798 | 0.012027 | 71 | 0.888306 | 0.007990 |  |  |  |
| 32 | 0.490825 | 0.011863 | 72 | 0.896296 | 0.007774 |  |  |  |
| 33 | 0.502688 | 0.011711 | 73 | 0.904070 | 0.007546 |  |  |  |
| 34 | 0.514399 | 0.011661 | 74 | 0.911616 | 0.007319 |  |  |  |
| 35 | 0.526060 | 0.011457 | 75 | 0.918934 | 0.007052 |  |  |  |
| 36 | 0.537518 | 0.011344 | 76 | 0.925986 | 0.006785 |  |  |  |
| 37 | 0.548861 | 0.011236 | 77 | 0.932771 | 0.006504 |  |  |  |
| 38 | 0.560097 | 0.011135 | 78 | 0.939275 | 0.006210 |  |  |  |
| 39 | 0.571232 | 0.011108 | 79 | 0.945485 | 0.005917 |  |  |  |

## Ash (Fraxinus *excelsior*)

Regression analysis

Age-height and age-circumference mean tree growth development provided in yield tables (general tables) of experimental silviculture plots of fully stocked (normal) Ash stands of Northern Eurasia [20] . Data for growth estimations taken from productivity class 2 (out of four yield classes), listed In Table 32.

Table 32: Mean growth of Ash (*f. excelsior*) stands, productivity class 2, Northern Eurasia

| Year | Top height | Diameter breast height | Circumference |
| --- | --- | --- | --- |
| [yr] | [m] | [cm] | [cm] |
| 20 | 12.1 | 11.3 | 35.50 |
| 30 | 17.8 | 17.1 | 53.72 |
| 40 | 22.3 | 22.3 | 70.06 |
| 50 | 25.6 | 26.9 | 84.51 |
| 60 | 28 | 30.8 | 96.76 |
| 70 | 29.8 | 34.2 | 107.44 |
| 80 | 31.1 | 37 | 116.24 |
| 90 | 32 | 39.4 | 123.78 |
| 100 | 32.6 | 41.5 | 130.38 |
| 110 | 33.1 | 43.2 | 135.72 |
| 120 | 33.4 | 44.6 | 140.12 |
| 130 | 33.7 | 45.9 | 144.20 |
| 140 | 33.8 | 46.9 | 147.34 |
| 150 | 33.9 | 47.7 | 149.85 |
| 160 | 34 | 48.4 | 152.05 |
| Source: [14, p. 108] | | | |

Model parameter results are illustrated in Table 33. The goodness of fit (GoF) for both age-height and age-circumference corresponds to 1. Results are plotted in Figure 34 and Figure 35, showing a comparison of the GoF from experimental yield table values and the fitted model.

Table 33: Characteristics and model fitting data of Ash (*f. excelsior*)

|  | A | k | p | RSS | GoF |
| --- | --- | --- | --- | --- | --- |
| Age-height growth | 34.19608 | 0.03488 | 1.50764 | 0.008973 | 1 |
| Age-circumference growth | 163.26052 | 0.01832 | 1.29068 | 0.1096 | 1 |

| 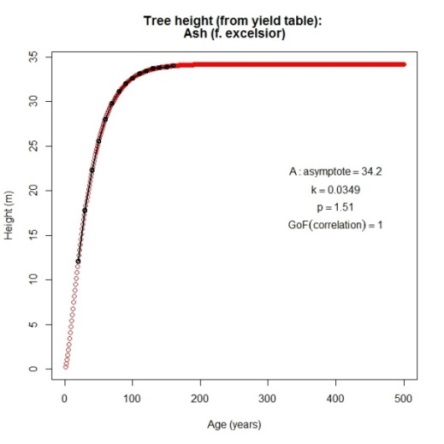 | 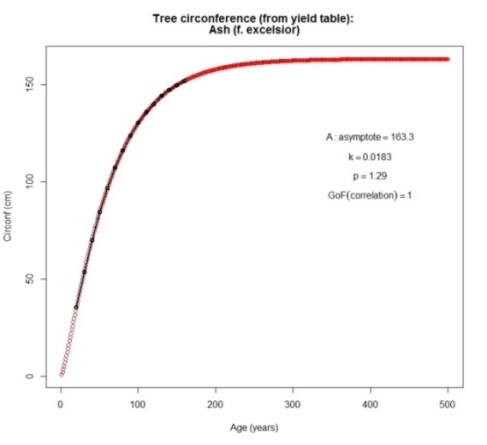 |
| --- | --- |
| Figure 34: Age-height mean growth curve and model parameter results. Black dots represent yield table data, red circles model-predicted values | Figure 35: Age-circumference mean growth curve and model parameter results. Black dots represent yield table data, red circles model-predicted values |

Biomass growth

Cutting/thinning operations in accordance with the yield table (Table 34) to represent growth and carbon sequestration dynamics per species and year. Initial density from seedlings is 1649 stems and first thinning period at age 20 with ten-year rotation cycles. The dominant height is calculated from the Chapman-Richards model by non-linear regression. The model is flexible and adaptable to any management interventions. The total rotation length is 86 years which also represents the year of final cut (clear cut of all remaining stands). In total 15 cutting interventions are performed (including final cut).

Table 34: Ash even-aged forest stand forest management practices with rotation length of 160 years

|  |  | Age | Top height | Density before cut | Stems removed | Stems removed |
| --- | --- | --- | --- | --- | --- | --- |
|  |  | [yr] | [m] | [stem·ha^-1^] | [stem·ha^-1^] | [%] |
| Rotation length | 160 | - | - | - | - | - |
| Total thinning operations | 15 | - | - | - | - | - |
| Initial density | 1649 | - | - | - | - | - |
| Cut 1 | - | 20 | 12 | 922 | 727 | 44% |
| Cut 2 | - | 30 | 18 | 623 | 299 | 18% |
| Cut 3 | - | 40 | 22 | 468 | 155 | 9% |
| Cut 4 | - | 50 | 26 | 376 | 92 | 6% |
| Cut 5 | - | 60 | 28 | 316 | 60 | 4% |
| Cut 6 | - | 70 | 30 | 276 | 40 | 2% |
| Cut 7 | - | 80 | 31 | 247 | 29 | 2% |
| Cut 8 | - | 90 | 32 | 225 | 22 | 1% |
| Cut 9 | - | 100 | 33 | 209 | 16 | 1% |
| Cut 10 | - | 110 | 33 | 197 | 12 | 1% |
| Cut 11 | - | 120 | 33 | 187 | 10 | 1% |
| Cut 12 | - | 130 | 34 | 180 | 7 | 0% |
| Cut 13 | - | 140 | 34 | 174 | 6 | 0% |
| Cut 14 | - | 150 | 34 | 169 | 5 | 0% |
| Final clear cut | - | 160 | 34 | 0 | 169 | 10% |
| Source: [14, p. 108] and fitted data | | | | | | |

Aboveground volume estimations based on allometric equation and coefficients. Growth dynamics of individual stem volume (Figure 36) and managed stand (Figure 37), with 2.49 m^3^ and 419 m^3^·ha^-1^ respectively over 160-years rotation cycle.

| 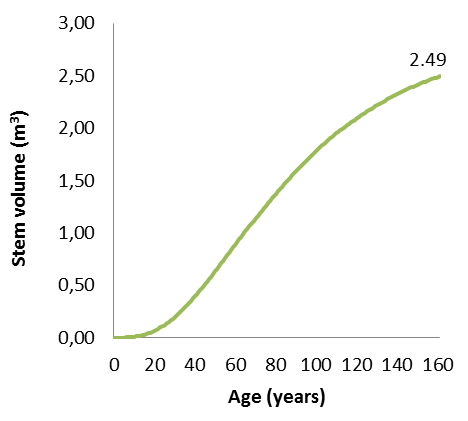 | 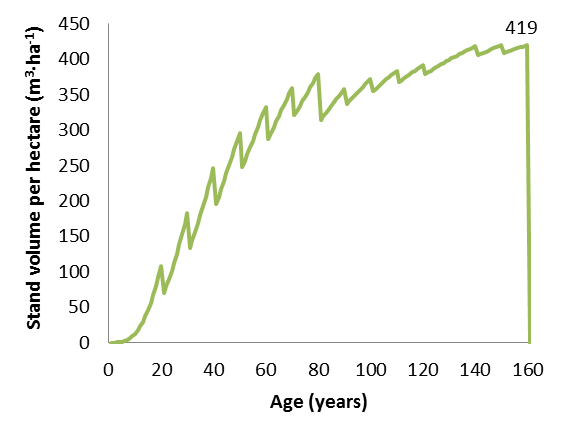 |
| --- | --- |
| Figure 36: Ash (*f. excelsior*) mean aboveground individual stem volume (m^3^) over 160-year rotation cycle | Figure 37: Ash (*f. excelsior*) mean aboveground volume per hectare (m^3^·ha^-1^) with thinning operations and clear cut at age 160 |

**Carbon** fixation

Carbon sequestration and storage of Ash (*f. excelsior*) over a 160-year rotation cycle (Table 35). Stocking factors applicable for rotation cycles before harvest (historic modelling approach).

Table 35: C_bio_ stocking factors for Ash (*f. excelsior*), 160-year rotation cycle

| Age  [yr] | C_bio_  growth  [t C_bio]_ | Annual C_bio_ stocking factors  [t C_bio_·yr^-1^] | Age  [yr] | C_bio_  growth  [t C_bio]_ | Annual C_bio_ stocking factors  [t C_bio_·yr^-1^] | Age  [yr] | C_bio_  growth  [t C_bio]_ | Annual C_bio_ stocking factors  [t C_bio_·yr^-1^] |
| --- | --- | --- | --- | --- | --- | --- | --- | --- |
| 0 | 0.000000 | 0.027996 | 60 | 0.648851 | 0.004131 | 120 | 0.899237 | 0.004432 |
| 1 | 0.027996 | 0.027287 | 61 | 0.652983 | 0.004104 | 121 | 0.903670 | 0.004406 |
| 2 | 0.055283 | 0.026531 | 62 | 0.657087 | 0.004076 | 122 | 0.908076 | 0.004374 |
| 3 | 0.081814 | 0.025731 | 63 | 0.661162 | 0.004048 | 123 | 0.912450 | 0.004337 |
| 4 | 0.107545 | 0.024888 | 64 | 0.665210 | 0.004021 | 124 | 0.916787 | 0.004294 |
| 5 | 0.132433 | 0.024007 | 65 | 0.669231 | 0.003996 | 125 | 0.921081 | 0.004245 |
| 6 | 0.156441 | 0.023091 | 66 | 0.673227 | 0.003973 | 126 | 0.925326 | 0.004190 |
| 7 | 0.179532 | 0.022144 | 67 | 0.677199 | 0.003954 | 127 | 0.929516 | 0.004130 |
| 8 | 0.201676 | 0.021173 | 68 | 0.681153 | 0.003939 | 128 | 0.933646 | 0.004063 |
| 9 | 0.222849 | 0.020183 | 69 | 0.685092 | 0.003930 | 129 | 0.937709 | 0.003991 |
| 10 | 0.243031 | 0.019182 | 70 | 0.689022 | 0.003926 | 130 | 0.941700 | 0.003912 |
| 11 | 0.262214 | 0.018180 | 71 | 0.692948 | 0.003922 | 131 | 0.945613 | 0.003827 |
| 12 | 0.280394 | 0.017186 | 72 | 0.696869 | 0.003917 | 132 | 0.949439 | 0.003733 |
| 13 | 0.297580 | 0.016213 | 73 | 0.700787 | 0.003913 | 133 | 0.953172 | 0.003633 |
| 14 | 0.313793 | 0.015272 | 74 | 0.704700 | 0.003910 | 134 | 0.956805 | 0.003525 |
| 15 | 0.329065 | 0.014378 | 75 | 0.708610 | 0.003908 | 135 | 0.960330 | 0.003410 |
| 16 | 0.343443 | 0.013547 | 76 | 0.712518 | 0.003907 | 136 | 0.963740 | 0.003287 |
| 17 | 0.356990 | 0.012796 | 77 | 0.716425 | 0.003910 | 137 | 0.967027 | 0.003158 |
| 18 | 0.369786 | 0.012142 | 78 | 0.720335 | 0.003915 | 138 | 0.970185 | 0.003022 |
| 19 | 0.381928 | 0.011600 | 79 | 0.724250 | 0.003925 | 139 | 0.973207 | 0.002880 |
| 20 | 0.393528 | 0.011156 | 80 | 0.728174 | 0.003937 | 140 | 0.976087 | 0.002732 |
| 21 | 0.404684 | 0.010728 | 81 | 0.732112 | 0.003950 | 141 | 0.978819 | 0.002577 |
| 22 | 0.415412 | 0.010299 | 82 | 0.736062 | 0.003963 | 142 | 0.981396 | 0.002417 |
| 23 | 0.425712 | 0.009876 | 83 | 0.740025 | 0.003976 | 143 | 0.983813 | 0.002251 |
| 24 | 0.435588 | 0.009464 | 84 | 0.744001 | 0.003989 | 144 | 0.986064 | 0.002080 |
| 25 | 0.445052 | 0.009068 | 85 | 0.747990 | 0.004003 | 145 | 0.988144 | 0.001906 |
| 26 | 0.454119 | 0.008695 | 86 | 0.751993 | 0.004018 | 146 | 0.990049 | 0.001729 |
| 27 | 0.462814 | 0.008351 | 87 | 0.756011 | 0.004035 | 147 | 0.991778 | 0.001551 |
| 28 | 0.471165 | 0.008046 | 88 | 0.760046 | 0.004054 | 148 | 0.993329 | 0.001373 |
| 29 | 0.479211 | 0.007784 | 89 | 0.764100 | 0.004075 | 149 | 0.994701 | 0.001196 |
| 30 | 0.486996 | 0.007561 | 90 | 0.768175 | 0.004099 | 150 | 0.995898 | 0.001023 |
| 31 | 0.494556 | 0.007342 | 91 | 0.772274 | 0.004122 | 151 | 0.996921 | 0.000855 |
| 32 | 0.501898 | 0.007123 | 92 | 0.776396 | 0.004145 | 152 | 0.997776 | 0.000694 |
| 33 | 0.509021 | 0.006905 | 93 | 0.780541 | 0.004167 | 153 | 0.998471 | 0.000543 |
| 34 | 0.515926 | 0.006691 | 94 | 0.784709 | 0.004189 | 154 | 0.999014 | 0.000403 |
| 35 | 0.522617 | 0.006485 | 95 | 0.788898 | 0.004211 | 155 | 0.999417 | 0.000280 |
| 36 | 0.529101 | 0.006289 | 96 | 0.793108 | 0.004232 | 156 | 0.999697 | 0.000174 |
| 37 | 0.535391 | 0.006108 | 97 | 0.797341 | 0.004255 | 157 | 0.999871 | 0.000091 |
| 38 | 0.541499 | 0.005946 | 98 | 0.801595 | 0.004278 | 158 | 0.999962 | 0.000034 |
| 39 | 0.547445 | 0.005806 | 99 | 0.805873 | 0.004302 | 159 | 0.999995 | 0.000005 |
| 40 | 0.553251 | 0.005684 | 100 | 0.810175 | 0.004326 | 160 | 1.000000 | 0.000000 |
| 41 | 0.558935 | 0.005565 | 101 | 0.814501 | 0.004349 |  |  |  |
| 42 | 0.564500 | 0.005445 | 102 | 0.818850 | 0.004371 |  |  |  |
| 43 | 0.569945 | 0.005325 | 103 | 0.823221 | 0.004391 |  |  |  |
| 44 | 0.575270 | 0.005207 | 104 | 0.827612 | 0.004410 |  |  |  |
| 45 | 0.580478 | 0.005094 | 105 | 0.832022 | 0.004427 |  |  |  |
| 46 | 0.585571 | 0.004986 | 106 | 0.836449 | 0.004442 |  |  |  |
| 47 | 0.590558 | 0.004887 | 107 | 0.840891 | 0.004457 |  |  |  |
| 48 | 0.595445 | 0.004799 | 108 | 0.845348 | 0.004470 |  |  |  |
| 49 | 0.600244 | 0.004723 | 109 | 0.849818 | 0.004483 |  |  |  |
| 50 | 0.604967 | 0.004659 | 110 | 0.854301 | 0.004494 |  |  |  |
| 51 | 0.609626 | 0.004595 | 111 | 0.858796 | 0.004503 |  |  |  |
| 52 | 0.614221 | 0.004531 | 112 | 0.863299 | 0.004508 |  |  |  |
| 53 | 0.618752 | 0.004467 | 113 | 0.867807 | 0.004510 |  |  |  |
| 54 | 0.623220 | 0.004405 | 114 | 0.872317 | 0.004509 |  |  |  |
| 55 | 0.627624 | 0.004345 | 115 | 0.876826 | 0.004504 |  |  |  |
| 56 | 0.631969 | 0.004289 | 116 | 0.881330 | 0.004497 |  |  |  |
| 57 | 0.636258 | 0.004239 | 117 | 0.885827 | 0.004486 |  |  |  |
| 58 | 0.640497 | 0.004195 | 118 | 0.890313 | 0.004471 |  |  |  |
| 59 | 0.644692 | 0.004160 | 119 | 0.894784 | 0.004454 |  |  |  |

## European beech (*Fagus sylvatica*)

Regression analysis

Age-height and age-circumference mean tree growth development provided in yield tables (general tables) of experimental silviculture plots by Waters and Christies (1958) [18] at the North-West of France. Data for growth estimations taken from productivity class 6 (out of four yield classes), listed in Table 36.

Table 36: Mean growth of European beech (*f. sylvatica*), productivity class 6, North-West (Bretagne)

| Year | Top height | Diameter breast height | Circumference |
| --- | --- | --- | --- |
| [yr] | [m] | [cm] | [cm] |
| 30 | 11.4 | 6.9 | 21.68 |
| 35 | 13.1 | 7.5 | 23.56 |
| 40 | 14.5 | 8.6 | 27.02 |
| 45 | 15.9 | 9.9 | 31.10 |
| 50 | 17.1 | 11.5 | 36.13 |
| 55 | 18.2 | 13.2 | 41.47 |
| 60 | 19.1 | 15.1 | 47.44 |
| 65 | 20.0 | 17.3 | 54.35 |
| 70 | 20.9 | 19.5 | 61.26 |
| 75 | 21.6 | 21.8 | 68.49 |
| 80 | 22.3 | 24.0 | 75.40 |
| 85 | 22.9 | 26.3 | 82.62 |
| 90 | 23.5 | 28.6 | 89.85 |
| 95 | 24.0 | 30.9 | 97.08 |
| 100 | 24.4 | 33.1 | 103.99 |
| 105 | 24.8 | 35.2 | 110.58 |
| 110 | 25.2 | 37.4 | 117.50 |
| 115 | 25.5 | 39.3 | 123.46 |
| 120 | 25.7 | 41.4 | 130.06 |
| 125 | 25.9 | 43.3 | 136.03 |
| 130 | 26.1 | 45.2 | 142.00 |
| 135 | 26.2 | 47.1 | 147.97 |
| 140 | 26.3 | 48.8 | 153.31 |
| 145 | 26.4 | 50.7 | 159.28 |
| 150 | 26.5 | 52.4 | 164.62 |
| Source: [11, p. 84] | | | |

Model parameter results are illustrated in Table 37. The goodness of fit (GoF) for both age-height and age-circumference corresponds to 1 and 0.999 respectively. Results are plotted in Figure 38 and Figure 39, showing a comparison of the GoF from experimental yield table values and the fitted model.

Table 37: Characteristics and model fitting data of Beech (*f. sylvatica*)

|  | A | k | p | RSS | GoF |
| --- | --- | --- | --- | --- | --- |
| Age-height growth | 27.74348 | 0.02352 | 1.31107 | 76.58 | 1 |
| Age-circumference growth | 361.4 | 0.007341 | 1.921 | 0.2185 | 0.999 |

| 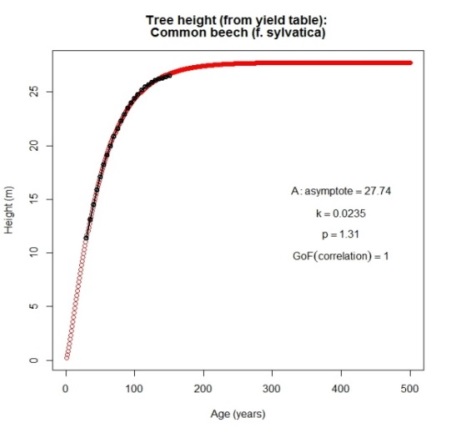 | 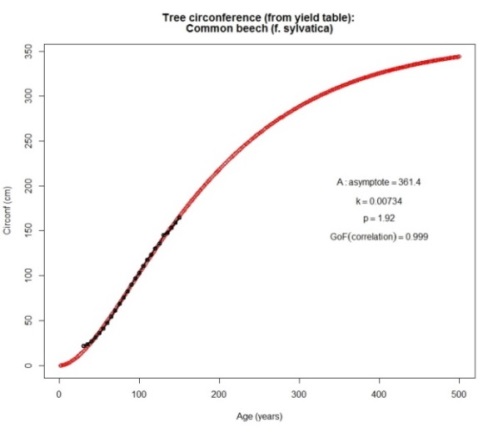 |
| --- | --- |
| Figure 38: Age-height mean growth curve and model parameter results of Beech (*f. sylvatica*). Black dots represent yield table data, red circles model-predicted values | Figure 39: Age-circumference mean growth curve and model parameter results Beech (*f. sylvatica*). Black dots represent yield table data, red circles model-predicted values |

Biomass growth

Cutting/thinning operations in accordance with the yield table (Table 38) to represent growth and carbon sequestration dynamics per species and year. Initial density from seedlings is 5035 trees and first thinning period at age 30 with five-year rotation cycles. The dominant height is calculated from the Chapman-Richards model by non-linear regression. The model is flexible and adaptable to any management interventions. The total rotation length is 86 years which also represents the year of final cut (clear cut of all remaining stands). In total25 cutting interventions are performed (including final cut).

Table 38: Beech even-aged forest stand forest management practices with rotation length of 150 years

| Stand interventions |  | Age | Top height | Density before cut | Stems removed | Stems removed |
| --- | --- | --- | --- | --- | --- | --- |
|  |  | [yr] | [m] | [stem·ha^-1^] | [stem·ha^-1^] | [%] |
| Rotation length | 150 | - | - | - | - | - |
| Total thinning operations | 25 | - | - | - | - | - |
| Initial density | 5035 | - | - | - | - | - |
| Cut 1 | - | 30 | 11.36 | 3946 | 1089.0 | 21.6% |
| Cut 2 | - | 35 | 13.00 | 2684 | 1262.0 | 25.1% |
| Cut 3 | - | 40 | 14.50 | 1928 | 756.0 | 15.0% |
| Cut 4 | - | 45 | 15.87 | 1423 | 505.0 | 10.0% |
| Cut 5 | - | 50 | 17.10 | 1087 | 336.0 | 6.7% |
| Cut 6 | - | 55 | 18.22 | 855 | 232 | 4.6% |
| Cut 7 | - | 60 | 19.23 | 691 | 164 | 3.3% |
| Cut 8 | - | 65 | 20.14 | 574 | 117 | 2.3% |
| Cut 9 | - | 70 | 20.95 | 487 | 87 | 1.7% |
| Cut 10 | - | 75 | 21.68 | 420 | 67 | 1.3% |
| Cut 11 | - | 80 | 22.34 | 367 | 53 | 1.1% |
| Cut 12 | - | 85 | 22.92 | 324 | 43 | 0.9% |
| Cut 13 | - | 90 | 23.45 | 289 | 35 | 0.7% |
| Cut 14 | - | 95 | 23.92 | 260 | 29 | 0.6% |
| Cut 15 | - | 100 | 24.33 | 237 | 23 | 0.5% |
| Cut 16 | - | 105 | 24.71 | 217 | 20 | 0.4% |
| Cut 17 | - | 110 | 25.04 | 201 | 16 | 0.3% |
| Cut 18 | - | 115 | 25.34 | 187 | 14 | 0.3% |
| Cut 19 | - | 120 | 25.60 | 174 | 13 | 0.3% |
| Cut 20 | - | 125 | 25.84 | 164 | 10 | 0.2% |
| Cut 21 | - | 130 | 26.05 | 154 | 10 | 0.2% |
| Cut 22 | - | 135 | 26.23 | 145 | 9 | 0.2% |
| Cut 23 | - | 140 | 26.40 | 137 | 8 | 0.2% |
| Cut 24 | - | 145 | 26.55 | 129 | 8 | 0.2% |
| Final clear cut | - | 150 | 26.68 | 0 | 129 | 2.6% |
| Source: [11, p. 84] and fitted data | | | | | | |

Aboveground volume estimations based on allometric equation and coefficients. Growth dynamics of individual stem volume (Figure 40) and managed stand (Figure 41), with 3.84 m^3^ and 487 m^3^·ha^-1^ respectively over 150-years rotation cycle.

| 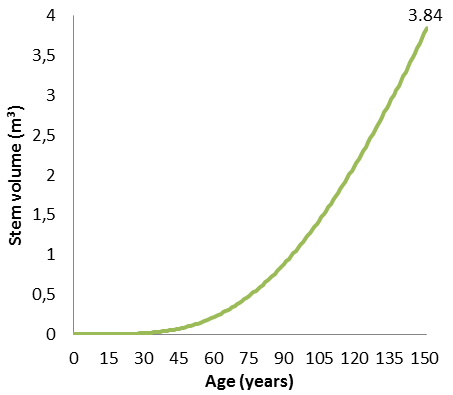  Figure 40: Beech (*f. sylvatica*) mean aboveground individual stem volume (m^3^) over 150-year rotation cycle | 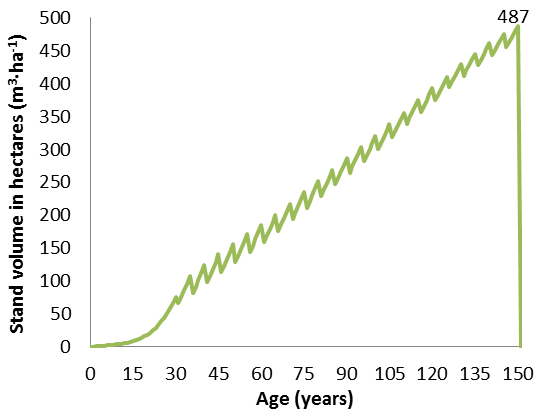  Figure 41: Beech (*f. sylvatica*) mean aboveground volume per hectare (m^3^·ha^-1^) with thinning operations and clear cut at age 150 |
| --- | --- |

Carbon fixation

Carbon sequestration and storage of Beech (*f. sylvatica*) over a 86-year rotation cycle (Table 39). Stocking factors applicable for rotation cycles before harvest (historic modelling approach).

Table 39: C_bio_ stocking factors for Beech (*f. sylvatica*), 150-year rotation cycle

| Age  [yr] | C_bio_  growth  [t C_bio]_ | Annual C_bio_ stocking factors  [t C_bio_·yr^-1^] | Age  [yr] | C_bio_  growth  [t C_bio]_ | Annual C_bio_ stocking factors  [t C_bio_·yr^-1^] | Age  [yr] | C_bio_  growth  [t C_bio]_ | Annual C_bio_ stocking factors  [t C_bio_·yr^-1^] |
| --- | --- | --- | --- | --- | --- | --- | --- | --- |
| 0 | 0.000000 | 0.035017 | 60 | 0.878328 | 0.003251 | 120 | 0.989952 | 0.000790 |
| 1 | 0.035017 | 0.034359 | 61 | 0.881579 | 0.003172 | 121 | 0.990742 | 0.000756 |
| 2 | 0.069375 | 0.033678 | 62 | 0.884751 | 0.003095 | 122 | 0.991498 | 0.000721 |
| 3 | 0.103053 | 0.032975 | 63 | 0.887846 | 0.003020 | 123 | 0.992219 | 0.000686 |
| 4 | 0.136028 | 0.032251 | 64 | 0.890866 | 0.002951 | 124 | 0.992905 | 0.000652 |
| 5 | 0.168279 | 0.031505 | 65 | 0.893817 | 0.002882 | 125 | 0.993557 | 0.000617 |
| 6 | 0.199784 | 0.030740 | 66 | 0.896699 | 0.002819 | 126 | 0.994175 | 0.000583 |
| 7 | 0.230524 | 0.029955 | 67 | 0.899518 | 0.002757 | 127 | 0.994758 | 0.000549 |
| 8 | 0.260480 | 0.029152 | 68 | 0.902275 | 0.002697 | 128 | 0.995307 | 0.000515 |
| 9 | 0.289632 | 0.028332 | 69 | 0.904972 | 0.002641 | 129 | 0.995822 | 0.000481 |
| 10 | 0.317964 | 0.027496 | 70 | 0.907612 | 0.002585 | 130 | 0.996303 | 0.000448 |
| 11 | 0.345460 | 0.026645 | 71 | 0.910197 | 0.002533 | 131 | 0.996750 | 0.000415 |
| 12 | 0.372105 | 0.025781 | 72 | 0.912730 | 0.002482 | 132 | 0.997165 | 0.000382 |
| 13 | 0.397886 | 0.024905 | 73 | 0.915211 | 0.002432 | 133 | 0.997547 | 0.000350 |
| 14 | 0.422792 | 0.024020 | 74 | 0.917643 | 0.002385 | 134 | 0.997897 | 0.000319 |
| 15 | 0.446812 | 0.023126 | 75 | 0.920028 | 0.002338 | 135 | 0.998216 | 0.000288 |
| 16 | 0.469938 | 0.022227 | 76 | 0.922366 | 0.002294 | 136 | 0.998504 | 0.000258 |
| 17 | 0.492165 | 0.021324 | 77 | 0.924659 | 0.002250 | 137 | 0.998762 | 0.000229 |
| 18 | 0.513489 | 0.020420 | 78 | 0.926910 | 0.002208 | 138 | 0.998992 | 0.000202 |
| 19 | 0.533909 | 0.019517 | 79 | 0.929117 | 0.002167 | 139 | 0.999193 | 0.000175 |
| 20 | 0.553427 | 0.018619 | 80 | 0.931284 | 0.002126 | 140 | 0.999368 | 0.000149 |
| 21 | 0.572046 | 0.017728 | 81 | 0.933411 | 0.002088 | 141 | 0.999517 | 0.000125 |
| 22 | 0.589774 | 0.016848 | 82 | 0.935498 | 0.002050 | 142 | 0.999643 | 0.000103 |
| 23 | 0.606622 | 0.015982 | 83 | 0.937548 | 0.002012 | 143 | 0.999745 | 0.000082 |
| 24 | 0.622604 | 0.015134 | 84 | 0.939560 | 0.001976 | 144 | 0.999827 | 0.000063 |
| 25 | 0.637737 | 0.014308 | 85 | 0.941536 | 0.001940 | 145 | 0.999890 | 0.000046 |
| 26 | 0.652045 | 0.013508 | 86 | 0.943476 | 0.001905 | 146 | 0.999936 | 0.000031 |
| 27 | 0.665553 | 0.012741 | 87 | 0.945380 | 0.001870 | 147 | 0.999967 | 0.000019 |
| 28 | 0.678294 | 0.012012 | 88 | 0.947250 | 0.001836 | 148 | 0.999986 | 0.000009 |
| 29 | 0.690305 | 0.011340 | 89 | 0.949086 | 0.001802 | 149 | 0.999995 | 0.000005 |
| 30 | 0.701645 | 0.010692 | 90 | 0.950889 | 0.001769 | 150 | 1.000000 | 0.000000 |
| 31 | 0.712336 | 0.010107 | 91 | 0.952657 | 0.001736 |  |  |  |
| 32 | 0.722444 | 0.009553 | 92 | 0.954394 | 0.001704 |  |  |  |
| 33 | 0.731997 | 0.009035 | 93 | 0.956097 | 0.001671 |  |  |  |
| 34 | 0.741031 | 0.008572 | 94 | 0.957768 | 0.001639 |  |  |  |
| 35 | 0.749603 | 0.008125 | 95 | 0.959408 | 0.001607 |  |  |  |
| 36 | 0.757728 | 0.007738 | 96 | 0.961015 | 0.001576 |  |  |  |
| 37 | 0.765465 | 0.007368 | 97 | 0.962590 | 0.001544 |  |  |  |
| 38 | 0.772834 | 0.007021 | 98 | 0.964134 | 0.001512 |  |  |  |
| 39 | 0.779854 | 0.006707 | 99 | 0.965646 | 0.001481 |  |  |  |
| 40 | 0.786561 | 0.006403 | 100 | 0.967127 | 0.001449 |  |  |  |
| 41 | 0.792963 | 0.006135 | 101 | 0.968575 | 0.001417 |  |  |  |
| 42 | 0.799099 | 0.005879 | 102 | 0.969993 | 0.001386 |  |  |  |
| 43 | 0.804978 | 0.005638 | 103 | 0.971378 | 0.001354 |  |  |  |
| 44 | 0.810615 | 0.005418 | 104 | 0.972732 | 0.001322 |  |  |  |
| 45 | 0.816033 | 0.005205 | 105 | 0.974054 | 0.001290 |  |  |  |
| 46 | 0.821238 | 0.005016 | 106 | 0.975344 | 0.001258 |  |  |  |
| 47 | 0.826254 | 0.004834 | 107 | 0.976602 | 0.001226 |  |  |  |
| 48 | 0.831089 | 0.004662 | 108 | 0.977828 | 0.001193 |  |  |  |
| 49 | 0.835751 | 0.004505 | 109 | 0.979021 | 0.001161 |  |  |  |
| 50 | 0.840256 | 0.004351 | 110 | 0.980182 | 0.001128 |  |  |  |
| 51 | 0.844607 | 0.004214 | 111 | 0.981309 | 0.001095 |  |  |  |
| 52 | 0.848821 | 0.004081 | 112 | 0.982404 | 0.001062 |  |  |  |
| 53 | 0.852902 | 0.003955 | 113 | 0.983466 | 0.001028 |  |  |  |
| 54 | 0.856857 | 0.003838 | 114 | 0.984494 | 0.000995 |  |  |  |
| 55 | 0.860695 | 0.003725 | 115 | 0.985488 | 0.000961 |  |  |  |
| 56 | 0.864420 | 0.003622 | 116 | 0.986449 | 0.000927 |  |  |  |
| 57 | 0.868041 | 0.003522 | 117 | 0.987376 | 0.000893 |  |  |  |
| 58 | 0.871563 | 0.003427 | 118 | 0.988269 | 0.000859 |  |  |  |
| 59 | 0.874990 | 0.003338 | 119 | 0.989128 | 0.000824 |  |  |  |

## Sessile oak (*Quercus petraea*)

Regression analysis

Age-height and age-circumference mean tree growth development provided in yield table (unique table) of experimental silviculture plot by J. P. Parde (1962) [18] at the Loire, as listed in Table 40.

Table 40: Mean growth of Sessile oak (*q. petraea*), unique table, Loire

| Year | Top height | Diameter breast height | Circumference |
| --- | --- | --- | --- |
| [yr] | [m] | [cm] | [cm] |
| 30 | 12.0 | 10.6 | 33.30 |
| 36 | 14.0 | 12.4 | 38.96 |
| 42 | 15.6 | 14 | 43.98 |
| 48 | 17.4 | 15.5 | 48.69 |
| 54 | 18.8 | 16.8 | 52.78 |
| 60 | 20.0 | 18 | 56.55 |
| 66 | 21.0 | 19.1 | 60.00 |
| 72 | 22.0 | 20.3 | 63.77 |
| 78 | 23.0 | 21.4 | 67.23 |
| 84 | 23.9 | 22.4 | 70.37 |
| 90 | 24.8 | 23.5 | 73.83 |
| 98 | 25.8 | 24.7 | 77.60 |
| 106 | 26.8 | 25.9 | 81.37 |
| 114 | 27.7 | 27 | 84.82 |
| 122 | 28.7 | 28.1 | 88.28 |
| 130 | 29.5 | 29 | 91.11 |
| 140 | 30.5 | 30 | 94.25 |
| 150 | 31.3 | 30.9 | 97.08 |
| 160 | 32.0 | 31.7 | 99.59 |
| 170 | 32.7 | 32.4 | 101.79 |
| 180 | 33.2 | 33 | 103.67 |
| 190 | 33.8 | 33.6 | 105.56 |
| 200 | 34.3 | 34.1 | 107.13 |
| Source: [18] | | | |

Model parameter results are illustrated in (Table 41). The goodness of fit (GoF) for both age-height and age-circumference corresponds to 1. Results are plotted in Figure 42 and Figure 43, showing a comparison of the GoF from experimental yield table values and the fitted model.

Table 41: Characteristics and model fitting data of Sessile oak (*q. petraea*)

|  | A | k | p | RSS | GoF |
| --- | --- | --- | --- | --- | --- |
| Age-height growth | 36.30192 | 0.02312 | 1.09369 | 0.00999 | 1 |
| Age-circumference growth | 230.50 | 0.007336 | 0.6648 | 0.02313 | 1 |

| 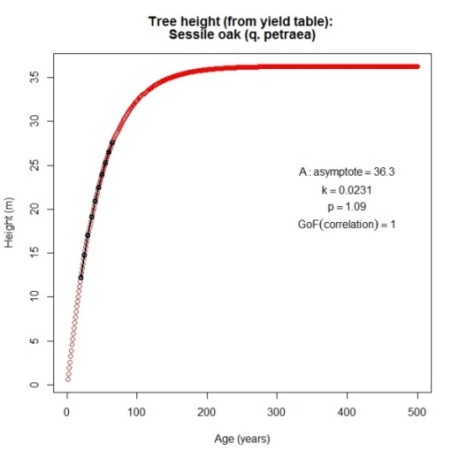 | 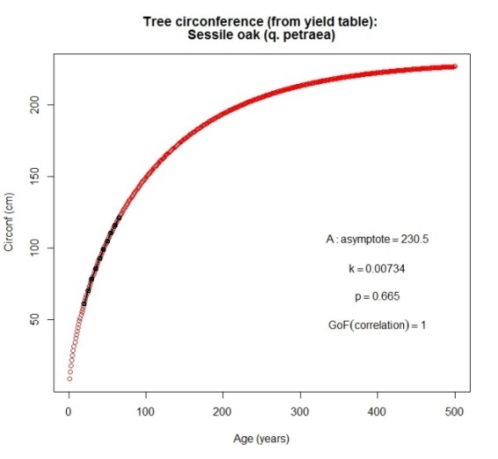 |
| --- | --- |
| Figure 42: Age-height mean growth curve and model parameter results of Sessile oak (q. petraea). Black dots represent yield table data, red circles model-predicted values | Figure 43: Age-circumference mean growth curve and model parameter results of Sessile oak (q. petraea). Black dots represent yield table data, red circles model-predicted values |

Biomass growth

Management regimes Cutting/thinning operations in accordance with the yield table (Table 42) to represent growth and carbon sequestration dynamics per species and year. Initial density from seedlings is 2740 trees and first thinning period at age 30 with mixed rotation cycles, it is to say six-year cycle for young stands, eight-year cycle for adult stands and ten-year cycle for mature trees. The dominant height is calculated from the Chapman-Richards model by non-linear regression. The total rotation length is 200 years which also represents the year of final cut (clear cut of all remaining stands). In total 23 cutting interventions are performed (including final cut).

Table 42: Sessile oak (q. petraea) even-aged forest stand forest management practices with rotation length of 200 years

| Stand interventions |  | Age | Top height | Density before cut | Stems removed | Stems removed |
| --- | --- | --- | --- | --- | --- | --- |
|  |  | [yr] | [m] | [stem·ha^-1^] | [stem·ha^-1^] | [%] |
| Rotation length | 200 | - | - | - | - | - |
| Total thinning operations | 23 | - | - | - | - | - |
| Initial density | 2740 | - | - |  | - | - |
| Cut 1 | - | 30 | 17 | 2240 | 500.0 | 18% |
| Cut 2 | - | 36 | 19 | 1840 | 400.0 | 15% |
| Cut 3 | - | 42 | 22 | 1520 | 320.0 | 12% |
| Cut 4 | - | 48 | 23 | 1200 | 320.0 | 12% |
| Cut 5 | - | 54 | 25 | 880 | 320.0 | 12% |
| Cut 6 | - | 60 | 27 | 736 | 144 | 5% |
| Cut 7 | - | 66 | 28 | 636 | 100 | 4% |
| Cut 8 | - | 72 | 29 | 552 | 84 | 3% |
| Cut 9 | - | 78 | 30 | 492 | 60 | 2% |
| Cut 10 | - | 84 | 31 | 444 | 48 | 2% |
| Cut 11 | - | 90 | 31 | 400 | 44 | 2% |
| Cut 12 | - | 98 | 32 | 356 | 44 | 2% |
| Cut 13 | - | 106 | 33 | 314 | 42 | 2% |
| Cut 14 | - | 114 | 33 | 272 | 42 | 2% |
| Cut 15 | - | 122 | 34 | 244 | 28 | 1% |
| Cut 16 | - | 130 | 34 | 220 | 24 | 1% |
| Cut 17 | - | 140 | 35 | 194 | 26 | 1% |
| Cut 18 | - | 150 | 35 | 176 | 18 | 1% |
| Cut 19 | - | 160 | 35 | 160 | 16 | 1% |
| Cut 20 | - | 170 | 36 | 148 | 12 | 0% |
| Cut 21 | - | 180 | 36 | 135 | 13 | 0% |
| Cut 22 | - | 190 | 36 | 127 | 8 | 0% |
| Final clear cut | - | 200 | 36 | 0 | 127 | 5% |
| Source: [18] and fitted data | | | | | | |

Aboveground volume estimations based on allometric equation and coefficients. Growth dynamics of individual stem volume (Figure 44) and managed stand (Figure 45), with 5.92 m^3^ and 749 m^3^·ha^-1^ respectively over 200-years rotation cycle.

| 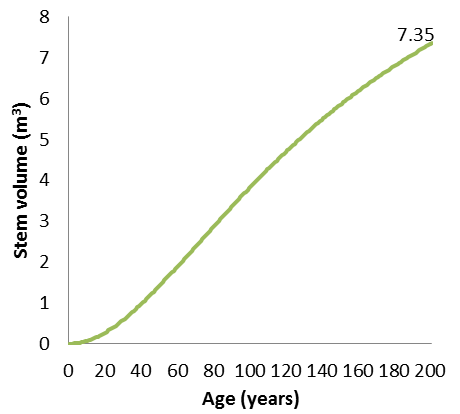 | 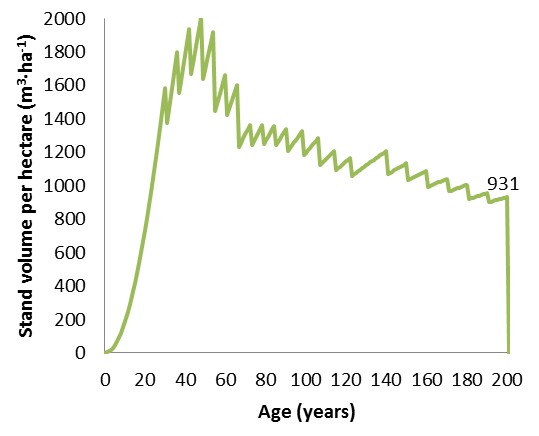 |
| --- | --- |
| Figure 44: Sessile oak (*q. petraea*) mean aboveground individual stem volume (m^3^) over 200-year rotation cycle | Figure 45: Sessile oak (*q. petraea*) mean aboveground volume per hectare (m^3^·ha^-1^) with thinning operations and clear cut at age 200 |

Carbon fixation

Carbon sequestration and storage of Sessile oak *(q. petraea*) over a 200-year rotation cycle (Table 43). Stocking factors applicable for rotation cycles before harvest (historic modelling approach).

Table 43: C_bio_ stocking factors for Sessile oak (*q. petraea),* 200-year rotation cycle

| Age  [yr] | C_bio_  growth  [t C_bio]_ | Annual C_bio_ stocking factors  [t C_bio_·yr^-1^] | Age  [yr] | C_bio_  growth  [t C_bio]_ | Annual C_bio_ stocking factors  [t C_bio_·yr^-1^] | Age  [yr] | C_bio_  growth  [t C_bio]_ | Annual C_bio_ stocking factors  [t C_bio_·yr^-1^] |
| --- | --- | --- | --- | --- | --- | --- | --- | --- |
| 0 | 0.000000 | 0.022844 | 70 | 0.767363 | 0.004403 | 140 | 0.961562 | 0.001500 |
| 1 | 0.022844 | 0.022429 | 71 | 0.771766 | 0.004337 | 141 | 0.963062 | 0.001469 |
| 2 | 0.045273 | 0.022010 | 72 | 0.776104 | 0.004273 | 142 | 0.964531 | 0.001439 |
| 3 | 0.067282 | 0.021587 | 73 | 0.780377 | 0.004210 | 143 | 0.965970 | 0.001408 |
| 4 | 0.088869 | 0.021160 | 74 | 0.784587 | 0.004148 | 144 | 0.967377 | 0.001377 |
| 5 | 0.110029 | 0.020731 | 75 | 0.788735 | 0.004087 | 145 | 0.968754 | 0.001346 |
| 6 | 0.130760 | 0.020298 | 76 | 0.792822 | 0.004027 | 146 | 0.970100 | 0.001315 |
| 7 | 0.151058 | 0.019863 | 77 | 0.796849 | 0.003969 | 147 | 0.971415 | 0.001284 |
| 8 | 0.170921 | 0.019426 | 78 | 0.800818 | 0.003912 | 148 | 0.972699 | 0.001253 |
| 9 | 0.190347 | 0.018988 | 79 | 0.804730 | 0.003856 | 149 | 0.973951 | 0.001222 |
| 10 | 0.209334 | 0.018548 | 80 | 0.808586 | 0.003800 | 150 | 0.975173 | 0.001191 |
| 11 | 0.227882 | 0.018107 | 81 | 0.812386 | 0.003746 | 151 | 0.976364 | 0.001160 |
| 12 | 0.245989 | 0.017666 | 82 | 0.816132 | 0.003692 | 152 | 0.977525 | 0.001129 |
| 13 | 0.263655 | 0.017225 | 83 | 0.819824 | 0.003639 | 153 | 0.978654 | 0.001098 |
| 14 | 0.280880 | 0.016785 | 84 | 0.823463 | 0.003588 | 154 | 0.979753 | 0.001067 |
| 15 | 0.297666 | 0.016347 | 85 | 0.827051 | 0.003537 | 155 | 0.980820 | 0.001036 |
| 16 | 0.314013 | 0.015911 | 86 | 0.830587 | 0.003486 | 156 | 0.981856 | 0.001005 |
| 17 | 0.329923 | 0.015477 | 87 | 0.834073 | 0.003437 | 157 | 0.982862 | 0.000974 |
| 18 | 0.345400 | 0.015047 | 88 | 0.837510 | 0.003388 | 158 | 0.983836 | 0.000943 |
| 19 | 0.360447 | 0.014621 | 89 | 0.840898 | 0.003339 | 159 | 0.984779 | 0.000913 |
| 20 | 0.375068 | 0.014200 | 90 | 0.844237 | 0.003292 | 160 | 0.985692 | 0.000882 |
| 21 | 0.389268 | 0.013785 | 91 | 0.847529 | 0.003246 | 161 | 0.986574 | 0.000851 |
| 22 | 0.403053 | 0.013376 | 92 | 0.850775 | 0.003199 | 162 | 0.987425 | 0.000821 |
| 23 | 0.416429 | 0.012976 | 93 | 0.853974 | 0.003153 | 163 | 0.988246 | 0.000790 |
| 24 | 0.429405 | 0.012584 | 94 | 0.857128 | 0.003108 | 164 | 0.989036 | 0.000759 |
| 25 | 0.441988 | 0.012201 | 95 | 0.860236 | 0.003064 | 165 | 0.989795 | 0.000729 |
| 26 | 0.454190 | 0.011830 | 96 | 0.863300 | 0.003020 | 166 | 0.990524 | 0.000699 |
| 27 | 0.466019 | 0.011470 | 97 | 0.866319 | 0.002977 | 167 | 0.991223 | 0.000668 |
| 28 | 0.477490 | 0.011124 | 98 | 0.869296 | 0.002934 | 168 | 0.991891 | 0.000638 |
| 29 | 0.488613 | 0.010792 | 99 | 0.872230 | 0.002893 | 169 | 0.992530 | 0.000609 |
| 30 | 0.499405 | 0.010476 | 100 | 0.875123 | 0.002852 | 170 | 0.993138 | 0.000579 |
| 31 | 0.509882 | 0.010171 | 101 | 0.877975 | 0.002811 | 171 | 0.993718 | 0.000550 |
| 32 | 0.520052 | 0.009874 | 102 | 0.880785 | 0.002770 | 172 | 0.994268 | 0.000521 |
| 33 | 0.529926 | 0.009588 | 103 | 0.883555 | 0.002730 | 173 | 0.994789 | 0.000492 |
| 34 | 0.539514 | 0.009312 | 104 | 0.886286 | 0.002691 | 174 | 0.995282 | 0.000464 |
| 35 | 0.548826 | 0.009049 | 105 | 0.888977 | 0.002653 | 175 | 0.995746 | 0.000436 |
| 36 | 0.557875 | 0.008798 | 106 | 0.891630 | 0.002615 | 176 | 0.996182 | 0.000408 |
| 37 | 0.566673 | 0.008557 | 107 | 0.894245 | 0.002578 | 177 | 0.996590 | 0.000381 |
| 38 | 0.575230 | 0.008323 | 108 | 0.896824 | 0.002541 | 178 | 0.996971 | 0.000354 |
| 39 | 0.583554 | 0.008098 | 109 | 0.899365 | 0.002505 | 179 | 0.997325 | 0.000328 |
| 40 | 0.591652 | 0.007882 | 110 | 0.901870 | 0.002469 | 180 | 0.997654 | 0.000303 |
| 41 | 0.599534 | 0.007677 | 111 | 0.904338 | 0.002433 | 181 | 0.997957 | 0.000278 |
| 42 | 0.607210 | 0.007482 | 112 | 0.906771 | 0.002398 | 182 | 0.998235 | 0.000254 |
| 43 | 0.614693 | 0.007296 | 113 | 0.909169 | 0.002363 | 183 | 0.998489 | 0.000230 |
| 44 | 0.621988 | 0.007116 | 114 | 0.911532 | 0.002329 | 184 | 0.998719 | 0.000207 |
| 45 | 0.629104 | 0.006944 | 115 | 0.913861 | 0.002296 | 185 | 0.998926 | 0.000185 |
| 46 | 0.636048 | 0.006780 | 116 | 0.916157 | 0.002262 | 186 | 0.999111 | 0.000164 |
| 47 | 0.642828 | 0.006625 | 117 | 0.918419 | 0.002229 | 187 | 0.999275 | 0.000144 |
| 48 | 0.649453 | 0.006480 | 118 | 0.920648 | 0.002195 | 188 | 0.999419 | 0.000124 |
| 49 | 0.655933 | 0.006342 | 119 | 0.922843 | 0.002162 | 189 | 0.999543 | 0.000106 |
| 50 | 0.662275 | 0.006208 | 120 | 0.925005 | 0.002129 | 190 | 0.999649 | 0.000089 |
| 51 | 0.668483 | 0.006081 | 121 | 0.927135 | 0.002097 | 191 | 0.999738 | 0.000073 |
| 52 | 0.674564 | 0.005959 | 122 | 0.929231 | 0.002065 | 192 | 0.999812 | 0.000059 |
| 53 | 0.680523 | 0.005845 | 123 | 0.931296 | 0.002033 | 193 | 0.999870 | 0.000045 |
| 54 | 0.686369 | 0.005738 | 124 | 0.933329 | 0.002001 | 194 | 0.999916 | 0.000034 |
| 55 | 0.692107 | 0.005635 | 125 | 0.935330 | 0.001969 | 195 | 0.999950 | 0.000023 |
| 56 | 0.697742 | 0.005535 | 126 | 0.937299 | 0.001937 | 196 | 0.999973 | 0.000015 |
| 57 | 0.703277 | 0.005437 | 127 | 0.939236 | 0.001905 | 197 | 0.999988 | 0.000008 |
| 58 | 0.708714 | 0.005342 | 128 | 0.941141 | 0.001874 | 198 | 0.999996 | 0.000003 |
| 59 | 0.714057 | 0.005251 | 129 | 0.943015 | 0.001842 | 199 | 0.999999 | 0.000001 |
| 60 | 0.719308 | 0.005164 | 130 | 0.944857 | 0.001811 | 200 | 1.000000 | 0.000000 |
| 61 | 0.724472 | 0.005079 | 131 | 0.946668 | 0.001780 |  |  |  |
| 62 | 0.729551 | 0.004996 | 132 | 0.948448 | 0.001749 |  |  |  |
| 63 | 0.734547 | 0.004914 | 133 | 0.950196 | 0.001717 |  |  |  |
| 64 | 0.739461 | 0.004835 | 134 | 0.951914 | 0.001686 |  |  |  |
| 65 | 0.744297 | 0.004758 | 135 | 0.953600 | 0.001655 |  |  |  |
| 66 | 0.749055 | 0.004684 | 136 | 0.955254 | 0.001623 |  |  |  |
| 67 | 0.753739 | 0.004612 | 137 | 0.956878 | 0.001592 |  |  |  |
| 68 | 0.758351 | 0.004541 | 138 | 0.958470 | 0.001561 |  |  |  |
| 69 | 0.762892 | 0.004471 | 139 | 0.960031 | 0.001530 |  |  |  |

## English oak (*Quercus robur*)

Regression analysis

Age-height and age-circumference mean tree growth development provided in yield tables of experimental silviculture plots, growth of full-stocked oak stands of optimal oak stands in the European part (ecoregions of zones of mixed forests, deciduous forests and forest steppe) [20]. Data for growth estimations taken from productivity class 1a (out of four yield classes), as listed in Table 44.

Table 44: Mean growth of English oak (*q. robur*), productivity class 1a, European part

| Year | Top height | Diameter breast height | Circumference |
| --- | --- | --- | --- |
| [yr] | [m] | [cm] | [cm] |
| 10 | 3.1 | 1.8 | 5.65 |
| 15 | 5.0 | 3.2 | 10.05 |
| 20 | 6.8 | 4.7 | 14.77 |
| 25 | 8.6 | 6.3 | 19.79 |
| 30 | 10.3 | 8 | 25.13 |
| 35 | 11.9 | 9.8 | 30.79 |
| 40 | 13.4 | 11.5 | 36.13 |
| 45 | 14.9 | 13.3 | 41.78 |
| 50 | 16.2 | 15.2 | 47.75 |
| 60 | 18.6 | 18.8 | 59.06 |
| 70 | 20.6 | 22.4 | 70.37 |
| 80 | 22.3 | 25.9 | 81.37 |
| 90 | 23.8 | 29.3 | 92.05 |
| 100 | 25.1 | 32.7 | 102.73 |
| 110 | 26.1 | 35.9 | 112.78 |
| 120 | 27.0 | 38.9 | 122.21 |
| 130 | 27.8 | 41.8 | 131.32 |
| 140 | 28.4 | 44.6 | 140.12 |
| 150 | 28.9 | 47.3 | 148.60 |
| 160 | 29.4 | 49.8 | 156.45 |
| Source: (Shvidenko et al. 2008, p. 294) | | | |

Model parameter results are illustrated in Table 45. The goodness of fit (GoF) for both age-height and age-circumference corresponds to 1. Results are plotted in Figure 46 and Figure 47, showing a comparison of the GoF from experimental yield table values and the fitted model.

Table 45: Characteristics and model fitting data of English oak (*q. robur*)

|  | A | k | p | RSS | GoF |
| --- | --- | --- | --- | --- | --- |
| Age-height growth | 31.64465 | 0.017950 | 1.281020 | 0.1685 | 1 |
| Age-circumference growth | 279.80000 | 0.00686 | 1.432000 | 0.01385 | 1 |

| 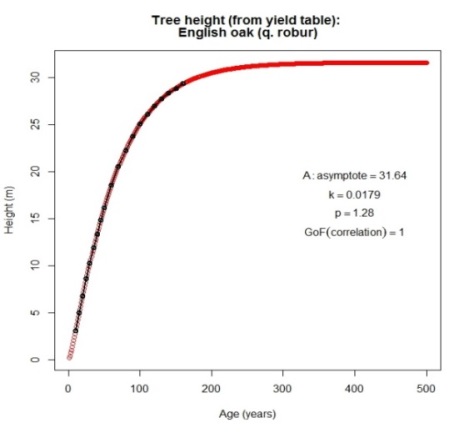 | 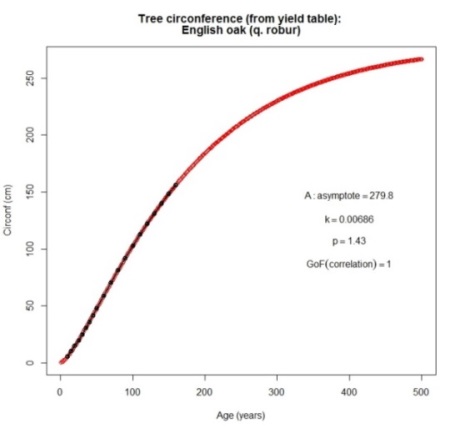 |
| --- | --- |
| Figure 46: Age-height mean growth curve and model parameter results of English oak (*q. robur*). Black dots represent yield table data, red circles model-predicted values | Figure 47: Age-circumference mean growth curve and model parameter results of English oak (*q. robur*). Black dots represent yield table data, red circles model-predicted values |

Biomass growth

Cutting/thinning operations in accordance with the yield table (Table 46) to represent growth and carbon sequestration dynamics per species and year. Initial density from seedlings is 28620 stems and first thinning period at age 30 with five-year rotation cycles followed by ten-year cycles. The dominant height is calculated from the Chapman-Richards model by non-linear regression. The total rotation length is 160 years which also represents the year of final cut (clear cut of all remaining stands). In total 20 cutting interventions are performed (including final cut).

Table 46: English oak (*q. robur*) even-aged forest stand forest management practices with rotation length of 160 years

| Stand interventions |  | Age | Top height | Density before cut | Stems removed | Stems removed |
| --- | --- | --- | --- | --- | --- | --- |
|  |  | [yr] | [m] | [stem·ha^-1^] | [stem·ha^-1^] | [%] |
| Rotation length | 160 | - | - | - | - | - |
| Total thinning operations | 20 | - | - | - | - | - |
| Initial density | 28620 | - | - | - | - | - |
| Cut 1 | - | 10 | 6 | 13570 | 15050 | 52.6% |
| Cut 2 | - | 15 | 9 | 7971 | 5599 | 19.6% |
| Cut 3 | - | 20 | 12 | 5268 | 2703 | 9.4% |
| Cut 4 | - | 25 | 15 | 3752 | 1516 | 5.3% |
| Cut 5 | - | 30 | 17 | 2815 | 937 | 3.3% |
| Cut 6 | - | 35 | 19 | 2195 | 620 | 2.2% |
| Cut 7 | - | 40 | 21 | 2195 | 0 | 0.0% |
| Cut 8 | - | 45 | 23 | 1762 | 433 | 1.5% |
| Cut 9 | - | 50 | 24 | 1442 | 320 | 1.1% |
| Cut 10 | - | 60 | 27 | 1033 | 409 | 1.4% |
| Cut 11 | - | 70 | 29 | 778 | 255 | 0.9% |
| Cut 12 | - | 80 | 30 | 610 | 168 | 0.6% |
| Cut 13 | - | 90 | 31 | 492 | 118 | 0.4% |
| Cut 14 | - | 100 | 32 | 410 | 82 | 0.3% |
| Cut 15 | - | 110 | 33 | 348 | 62 | 0.2% |
| Cut 16 | - | 120 | 34 | 300 | 48 | 0.2% |
| Cut 17 | - | 130 | 34 | 234 | 66 | 0.2% |
| Cut 18 | - | 140 | 35 | 210 | 24 | 0.1% |
| Cut 19 | - | 150 | 35 | 190 | 20 | 0.1% |
| Final clear cut | - | 160 | 35 | 0 | 190 | 0.7% |
| Source: [14, p. 294] and fitted data | | | | | | |

Aboveground volume estimations based on allometric equation and coefficients. Growth dynamics of individual stem volume (Figure 48) and managed stand (Figure 49), with 2.65 m^3^ and 194 m^3^·ha^-1^ respectively over 160-years rotation cycle.

| 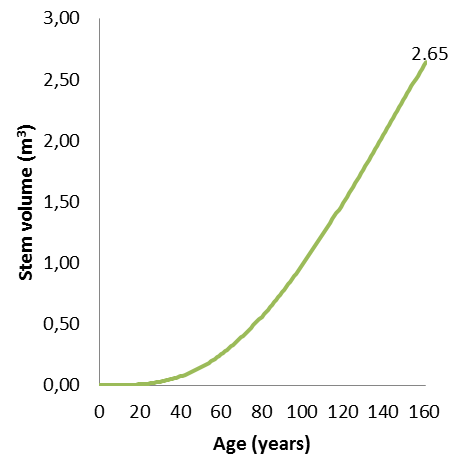 | 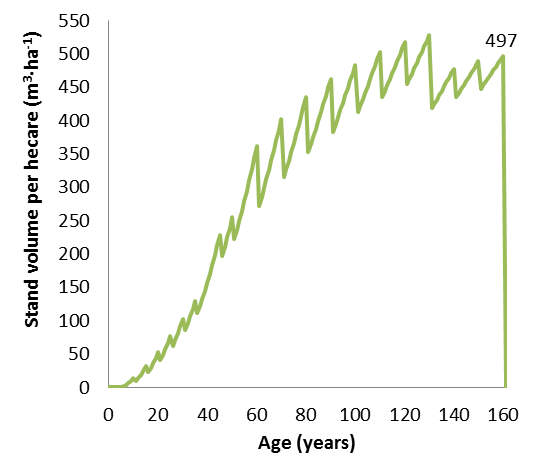 |
| --- | --- |
| Figure 48: English oak (*q. robur*) mean aboveground individual stem volume (m^3^) over 160-year rotation cycle | Figure 49: English oak (*q. robur*) mean aboveground volume per hectare (m^3^·ha^-1^) with thinning operations and clear cut at age 160 |

Carbon fixation

Carbon sequestration and storage of English oak (*q. robur*), 160-year rotation cycle (Table 47). Stocking factors applicable for rotation cycles before harvest (historic modelling approach).

Table 47: C_bio_ stocking factors for English oak (*q. robur*), 160-year rotation cycle

| Age  [yr] | C_bio_  growth  [t C_bio]_ | Annual C_bio_ stocking factors  [t C_bio_·yr^-1^] | Age  [yr] | C_bio_  growth  [t C_bio]_ | Annual C_bio_ stocking factors  [t C_bio_·yr^-1^] | Age  [yr] | C_bio_  growth  [ tC_bio]_ | Annual C_bio_ stocking factors  [t C_bio_·yr^-1^] |
| --- | --- | --- | --- | --- | --- | --- | --- | --- |
| 0 | 0.000000 | 0.033045 | 60 | 0.821904 | 0.005550 | 120 | 0.990642 | 0.000766 |
| 1 | 0.033045 | 0.031234 | 61 | 0.827455 | 0.005423 | 121 | 0.991408 | 0.000722 |
| 2 | 0.064279 | 0.029570 | 62 | 0.832878 | 0.005299 | 122 | 0.992130 | 0.000680 |
| 3 | 0.093848 | 0.028046 | 63 | 0.838177 | 0.005177 | 123 | 0.992811 | 0.000640 |
| 4 | 0.121894 | 0.026657 | 64 | 0.843353 | 0.005058 | 124 | 0.993451 | 0.000600 |
| 5 | 0.148551 | 0.025395 | 65 | 0.848411 | 0.004941 | 125 | 0.994051 | 0.000562 |
| 6 | 0.173946 | 0.024251 | 66 | 0.853352 | 0.004826 | 126 | 0.994613 | 0.000525 |
| 7 | 0.198197 | 0.023215 | 67 | 0.858178 | 0.004714 | 127 | 0.995138 | 0.000489 |
| 8 | 0.221412 | 0.022274 | 68 | 0.862893 | 0.004605 | 128 | 0.995628 | 0.000455 |
| 9 | 0.243686 | 0.021415 | 69 | 0.867497 | 0.004497 | 129 | 0.996083 | 0.000422 |
| 10 | 0.265101 | 0.020622 | 70 | 0.871994 | 0.004391 | 130 | 0.996504 | 0.000390 |
| 11 | 0.285722 | 0.019884 | 71 | 0.876385 | 0.004287 | 131 | 0.996894 | 0.000359 |
| 12 | 0.305606 | 0.019197 | 72 | 0.880672 | 0.004184 | 132 | 0.997253 | 0.000330 |
| 13 | 0.324803 | 0.018556 | 73 | 0.884856 | 0.004083 | 133 | 0.997583 | 0.000301 |
| 14 | 0.343359 | 0.017956 | 74 | 0.888939 | 0.003984 | 134 | 0.997884 | 0.000274 |
| 15 | 0.361315 | 0.017391 | 75 | 0.892923 | 0.003887 | 135 | 0.998159 | 0.000249 |
| 16 | 0.378705 | 0.016856 | 76 | 0.896810 | 0.003791 | 136 | 0.998408 | 0.000225 |
| 17 | 0.395562 | 0.016351 | 77 | 0.900601 | 0.003697 | 137 | 0.998632 | 0.000202 |
| 18 | 0.411913 | 0.015871 | 78 | 0.904299 | 0.003605 | 138 | 0.998834 | 0.000180 |
| 19 | 0.427783 | 0.015415 | 79 | 0.907903 | 0.003513 | 139 | 0.999014 | 0.000160 |
| 20 | 0.443199 | 0.014980 | 80 | 0.911417 | 0.003424 | 140 | 0.999173 | 0.000140 |
| 21 | 0.458178 | 0.014564 | 81 | 0.914840 | 0.003335 | 141 | 0.999314 | 0.000123 |
| 22 | 0.472742 | 0.014165 | 82 | 0.918175 | 0.003248 | 142 | 0.999436 | 0.000106 |
| 23 | 0.486907 | 0.013782 | 83 | 0.921423 | 0.003162 | 143 | 0.999543 | 0.000091 |
| 24 | 0.500689 | 0.013415 | 84 | 0.924584 | 0.003077 | 144 | 0.999634 | 0.000078 |
| 25 | 0.514104 | 0.013061 | 85 | 0.927661 | 0.002993 | 145 | 0.999712 | 0.000065 |
| 26 | 0.527165 | 0.012720 | 86 | 0.930654 | 0.002911 | 146 | 0.999777 | 0.000054 |
| 27 | 0.539885 | 0.012390 | 87 | 0.933565 | 0.002830 | 147 | 0.999830 | 0.000044 |
| 28 | 0.552275 | 0.012071 | 88 | 0.936395 | 0.002750 | 148 | 0.999874 | 0.000035 |
| 29 | 0.564345 | 0.011762 | 89 | 0.939145 | 0.002671 | 149 | 0.999909 | 0.000027 |
| 30 | 0.576108 | 0.011463 | 90 | 0.941817 | 0.002594 | 150 | 0.999937 | 0.000021 |
| 31 | 0.587571 | 0.011173 | 91 | 0.944410 | 0.002517 | 151 | 0.999957 | 0.000015 |
| 32 | 0.598744 | 0.010891 | 92 | 0.946927 | 0.002441 | 152 | 0.999973 | 0.000011 |
| 33 | 0.609635 | 0.010617 | 93 | 0.949369 | 0.002367 | 153 | 0.999984 | 0.000007 |
| 34 | 0.620252 | 0.010352 | 94 | 0.951735 | 0.002293 | 154 | 0.999991 | 0.000005 |
| 35 | 0.630604 | 0.010093 | 95 | 0.954029 | 0.002221 | 155 | 0.999996 | 0.000003 |
| 36 | 0.640697 | 0.009842 | 96 | 0.956250 | 0.002150 | 156 | 0.999998 | 0.000001 |
| 37 | 0.650540 | 0.009598 | 97 | 0.958399 | 0.002080 | 157 | 0.999999 | 0.000000 |
| 38 | 0.660138 | 0.009361 | 98 | 0.960479 | 0.002010 | 158 | 1.000000 | 0.000000 |
| 39 | 0.669499 | 0.009132 | 99 | 0.962489 | 0.001942 | 159 | 1.000000 | 0.000000 |
| 40 | 0.678631 | 0.008909 | 100 | 0.964432 | 0.001875 | 160 | 1.000000 | 0.000000 |
| 41 | 0.687540 | 0.008693 | 101 | 0.966307 | 0.001809 |  |  |  |
| 42 | 0.696232 | 0.008483 | 102 | 0.968117 | 0.001744 |  |  |  |
| 43 | 0.704715 | 0.008280 | 103 | 0.969861 | 0.001680 |  |  |  |
| 44 | 0.712995 | 0.008082 | 104 | 0.971541 | 0.001618 |  |  |  |
| 45 | 0.721077 | 0.007890 | 105 | 0.973159 | 0.001556 |  |  |  |
| 46 | 0.728967 | 0.007703 | 106 | 0.974715 | 0.001495 |  |  |  |
| 47 | 0.736670 | 0.007522 | 107 | 0.976210 | 0.001436 |  |  |  |
| 48 | 0.744192 | 0.007345 | 108 | 0.977646 | 0.001377 |  |  |  |
| 49 | 0.751537 | 0.007173 | 109 | 0.979023 | 0.001320 |  |  |  |
| 50 | 0.758710 | 0.007005 | 110 | 0.980343 | 0.001264 |  |  |  |
| 51 | 0.765715 | 0.006842 | 111 | 0.981607 | 0.001209 |  |  |  |
| 52 | 0.772557 | 0.006683 | 112 | 0.982816 | 0.001155 |  |  |  |
| 53 | 0.779240 | 0.006528 | 113 | 0.983971 | 0.001102 |  |  |  |
| 54 | 0.785768 | 0.006377 | 114 | 0.985073 | 0.001050 |  |  |  |
| 55 | 0.792145 | 0.006231 | 115 | 0.986123 | 0.001000 |  |  |  |
| 56 | 0.798375 | 0.006088 | 116 | 0.987123 | 0.000951 |  |  |  |
| 57 | 0.804463 | 0.005948 | 117 | 0.988074 | 0.000903 |  |  |  |
| 58 | 0.810411 | 0.005813 | 118 | 0.988976 | 0.000856 |  |  |  |
| 59 | 0.816224 | 0.005680 | 119 | 0.989832 | 0.000810 |  |  |  |

## White oak (*Quercus pubescens*)

Regression analysis

Age-height and age-circumference mean tree growth development provided in yield tables of experimental silviculture plots, growth of full-stocked oak stands of optimal oak stands in the European part (ecoregions of zones of mixed forests, deciduous forests and forest steppe) [20]. Data for growth estimations taken from productivity class 2 (out of four yield classes), as listed in Table 48.

Table 48: Mean growth of English oak (*q. pubescent*), productivity class 2, European part

| Year | Top height | Diameter breast height | Circumference |
| --- | --- | --- | --- |
| [yr] | [m] | [cm] | [cm] |
| 10 | 2.3 | 2.1 | 6.60 |
| 15 | 4.2 | 4.0 | 12.57 |
| 20 | 6.2 | 6.1 | 19.16 |
| 25 | 8.2 | 8.4 | 26.39 |
| 30 | 10.1 | 10.6 | 33.30 |
| 35 | 12.0 | 12.8 | 40.21 |
| 40 | 13.7 | 15.0 | 47.12 |
| 45 | 15.2 | 17.0 | 53.41 |
| 50 | 16.7 | 18.9 | 59.38 |
| 55 | 17.9 | 20.7 | 65.03 |
| 60 | 19.1 | 22.3 | 70.06 |
| 65 | 20.1 | 23.8 | 74.77 |
| 70 | 21.0 | 25.2 | 79.17 |
| 75 | 21.8 | 26.4 | 82.94 |
| 80 | 22.5 | 27.6 | 86.71 |
| 85 | 23.2 | 28.6 | 89.85 |
| 90 | 23.7 | 29.6 | 92.99 |
| 95 | 24.2 | 30.4 | 95.50 |
| 100 | 24.6 | 31.2 | 98.02 |
| 105 | 25.0 | 31.9 | 100.22 |
| 110 | 25.4 | 32.5 | 102.10 |
| 115 | 25.6 | 33.0 | 103.67 |
| 120 | 25.9 | 33.5 | 105.24 |
| Source: [14, p. 295] | | | |

Model parameter results are illustrated in Table 49. The goodness of fit (GoF) for both age-height and age-circumference corresponds to 1. Results are plotted in Figure 50 and Figure 51, showing a comparison of the GoF from experimental yield table values and the fitted model.

Table 49: Characteristics and model fitting data of White oak (*q. pubescent*)

|  | A | k | p | RSS | GoF |
| --- | --- | --- | --- | --- | --- |
| Age-height growth | 27.624010 | 0.027530 | 1.739690 | 0.1508 | 1 |
| Age-circumference growth | 118.430950 | 0.023180 | 1.833670 | 0.01765 | 1 |

| 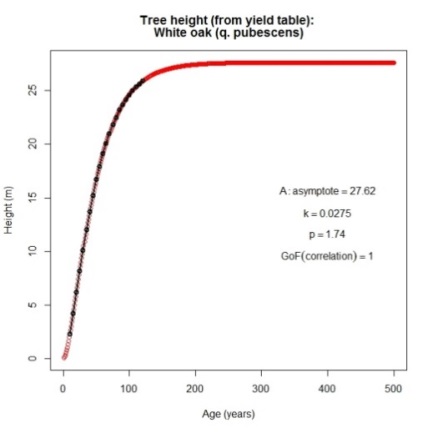 | 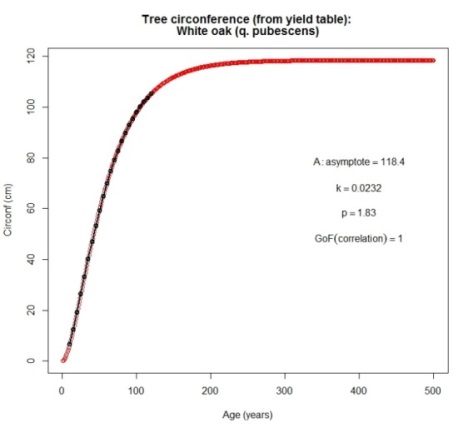 |
| --- | --- |
| Figure 50: Age-height mean growth curve and model parameter results of White oak (*q. pubescent*). Black dots represent yield table data, red circles model-predicted values | Figure 51: Age-circumference mean growth curve and model parameter results of White oak (*q. pubescent*). Black dots represent yield table data, red circles model-predicted values |

Biomass growth

Cutting/thinning operations in accordance with the yield table (Table 50) to represent growth and carbon sequestration dynamics per species and year. Initial density from seedlings is 8747 trees and first thinning period at age 10 with five-year rotation cycles. The dominant height is calculated from the Chapman-Richards model by non-linear regression. The total rotation length is 120 years which also represents the year of final cut (clear cut of all remaining stands). In total 23 cutting interventions are performed (including final cut).

Table 50: White oak (*q. pubescent*) even-aged forest stand forest management practices with rotation length of 120 years

| Stand interventions |  | Age | Top height | Density before cut | Stems removed | Stems removed |
| --- | --- | --- | --- | --- | --- | --- |
|  |  | [yr] | [m] | [stem·ha^-1^] | [stem·ha^-1^] | [%] |
| Rotation length | 120 | - | - | - | - | - |
| Total thinning operations | 23 | - | - | - | - | - |
| Initial density | 8747 | - | - | - | - | - |
| Cut 1 | - | 10 | 2 | 5030 | 3717 | 42.5% |
| Cut 2 | - | 15 | 4 | 3365 | 1665 | 19.0% |
| Cut 3 | - | 20 | 6 | 2452 | 913 | 10.4% |
| Cut 4 | - | 25 | 8 | 1891 | 561 | 6.4% |
| Cut 5 | - | 30 | 10 | 1519 | 372 | 4.3% |
| Cut 6 | - | 35 | 12 | 1219 | 300 | 3.4% |
| Cut 7 | - | 40 | 14 | 1069 | 150 | 1.7% |
| Cut 8 | - | 45 | 15 | 927 | 142 | 1.6% |
| Cut 9 | - | 50 | 17 | 817 | 110 | 1.3% |
| Cut 10 | - | 55 | 18 | 731 | 86 | 1.0% |
| Cut 11 | - | 60 | 19 | 663 | 68 | 0.8% |
| Cut 12 | - | 65 | 20 | 607 | 56 | 0.6% |
| Cut 13 | - | 70 | 21 | 562 | 45 | 0.5% |
| Cut 14 | - | 75 | 22 | 524 | 38 | 0.4% |
| Cut 15 | - | 80 | 23 | 493 | 31 | 0.4% |
| Cut 16 | - | 85 | 23 | 467 | 26 | 0.3% |
| Cut 17 | - | 90 | 24 | 444 | 23 | 0.3% |
| Cut 18 | - | 95 | 24 | 425 | 19 | 0.2% |
| Cut 19 | - | 100 | 25 | 409 | 16 | 0.2% |
| Cut 20 | - | 105 | 25 | 395 | 14 | 0.2% |
| Cut 21 | - | 110 | 25 | 383 | 12 | 0.1% |
| Cut 22 | - | 115 | 26 | 373 | 10 | 0.1% |
| Final clear cut | - | 120 | 26 | 120 | 373 | 4.3% |

Source: [14, p. 295] and fitted data

Aboveground volume estimations based on allometric equation and coefficients. Growth dynamics of individual stem volume (Figure 52) and managed stand (Figure 53), with 1.42 m^3^ and 526 m^3^·ha^-1^ respectively over 120-years rotation cycle.

| 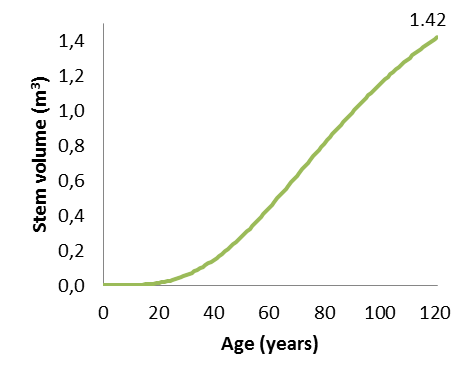 | 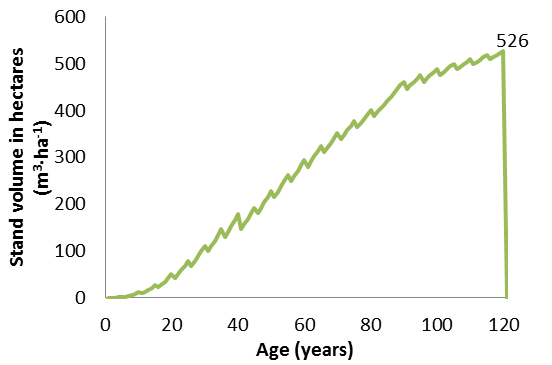 |
| --- | --- |
| Figure 52: White oak (*q. pubescent*) mean aboveground individual stem volume (m^3^) over 120-year rotation cycle | Figure 53: White oak (*q. pubescent*) mean aboveground volume per hectare (m^3^·ha^-1^) with thinning operations and clear cut at age 120 |

Carbon fixation

Carbon sequestration and storage of Norway White oak (*q. pubescent*) over a 120-year rotation cycle (Table 51). Stocking factors applicable for rotation cycles before harvest (historic modelling approach).

Table 51: C_bio_ stocking factors for White oak (*q. pubescent*), 120-year rotation cycle

| Age  [yr] | C_bio_  growth  [t C_bio]_ | Annual C_bio_ stocking factors  [t C_bio_·yr^-1^] | Age  [yr] | C_bio_  growth  [t C_bio]_ | Annual C_bio_ stocking factors  [t C_bio_·yr^-1^] | Age  [yr] | C_bio_  growth  [t C_bio]_ | Annual C_bio_ stocking factors  [t C_bio_·yr^-1^] |
| --- | --- | --- | --- | --- | --- | --- | --- | --- |
| 0 | 0.000000 | 0.045005 | 50 | 0.733562 | 0.005737 | 100 | 0.976582 | 0.002733 |
| 1 | 0.045005 | 0.042105 | 51 | 0.739299 | 0.005690 | 101 | 0.979315 | 0.002557 |
| 2 | 0.087110 | 0.039261 | 52 | 0.744989 | 0.005646 | 102 | 0.981873 | 0.002378 |
| 3 | 0.126371 | 0.036498 | 53 | 0.750636 | 0.005605 | 103 | 0.984251 | 0.002197 |
| 4 | 0.162869 | 0.033840 | 54 | 0.756240 | 0.005569 | 104 | 0.986448 | 0.002014 |
| 5 | 0.196709 | 0.031310 | 55 | 0.761809 | 0.005530 | 105 | 0.988462 | 0.001831 |
| 6 | 0.228019 | 0.028930 | 56 | 0.767340 | 0.005503 | 106 | 0.990293 | 0.001650 |
| 7 | 0.256949 | 0.026720 | 57 | 0.772843 | 0.005477 | 107 | 0.991943 | 0.001471 |
| 8 | 0.283669 | 0.024695 | 58 | 0.778319 | 0.005452 | 108 | 0.993414 | 0.001296 |
| 9 | 0.308364 | 0.022973 | 59 | 0.783771 | 0.005431 | 109 | 0.994710 | 0.001126 |
| 10 | 0.331337 | 0.021180 | 60 | 0.789202 | 0.005407 | 110 | 0.995837 | 0.000963 |
| 11 | 0.352517 | 0.019905 | 61 | 0.794609 | 0.005391 | 111 | 0.996800 | 0.000809 |
| 12 | 0.372422 | 0.018704 | 62 | 0.800000 | 0.005375 | 112 | 0.997609 | 0.000665 |
| 13 | 0.391126 | 0.017584 | 63 | 0.805375 | 0.005359 | 113 | 0.998273 | 0.000532 |
| 14 | 0.408710 | 0.016601 | 64 | 0.810734 | 0.005346 | 114 | 0.998805 | 0.000412 |
| 15 | 0.425311 | 0.015588 | 65 | 0.816080 | 0.005328 | 115 | 0.999217 | 0.000305 |
| 16 | 0.440898 | 0.014811 | 66 | 0.821408 | 0.005316 | 116 | 0.999522 | 0.000214 |
| 17 | 0.455710 | 0.014073 | 67 | 0.826725 | 0.005303 | 117 | 0.999736 | 0.000139 |
| 18 | 0.469783 | 0.013379 | 68 | 0.832028 | 0.005289 | 118 | 0.999875 | 0.000079 |
| 19 | 0.483162 | 0.012759 | 69 | 0.837316 | 0.005274 | 119 | 0.999954 | 0.000046 |
| 20 | 0.495922 | 0.012123 | 70 | 0.842591 | 0.005256 | 120 | 1.000000 | 0.000000 |
| 21 | 0.508045 | 0.011618 | 71 | 0.847847 | 0.005240 |  |  |  |
| 22 | 0.519663 | 0.011136 | 72 | 0.853086 | 0.005221 |  |  |  |
| 23 | 0.530799 | 0.010680 | 73 | 0.858307 | 0.005200 |  |  |  |
| 24 | 0.541479 | 0.010270 | 74 | 0.863507 | 0.005176 |  |  |  |
| 25 | 0.551749 | 0.009850 | 75 | 0.868683 | 0.005148 |  |  |  |
| 26 | 0.561599 | 0.009510 | 76 | 0.873831 | 0.005119 |  |  |  |
| 27 | 0.571109 | 0.009186 | 77 | 0.878950 | 0.005086 |  |  |  |
| 28 | 0.580295 | 0.008879 | 78 | 0.884036 | 0.005049 |  |  |  |
| 29 | 0.589174 | 0.008602 | 79 | 0.889086 | 0.005008 |  |  |  |
| 30 | 0.597777 | 0.008319 | 80 | 0.894094 | 0.004961 |  |  |  |
| 31 | 0.606096 | 0.008089 | 81 | 0.899055 | 0.004911 |  |  |  |
| 32 | 0.614185 | 0.007870 | 82 | 0.903965 | 0.004855 |  |  |  |
| 33 | 0.622055 | 0.007662 | 83 | 0.908821 | 0.004793 |  |  |  |
| 34 | 0.629717 | 0.007476 | 84 | 0.913614 | 0.004726 |  |  |  |
| 35 | 0.637193 | 0.007282 | 85 | 0.918340 | 0.004651 |  |  |  |
| 36 | 0.644474 | 0.007128 | 86 | 0.922992 | 0.004572 |  |  |  |
| 37 | 0.651602 | 0.006979 | 87 | 0.927564 | 0.004486 |  |  |  |
| 38 | 0.658581 | 0.006836 | 88 | 0.932050 | 0.004392 |  |  |  |
| 39 | 0.665418 | 0.006706 | 89 | 0.936442 | 0.004292 |  |  |  |
| 40 | 0.672124 | 0.006572 | 90 | 0.940735 | 0.004184 |  |  |  |
| 41 | 0.678696 | 0.006460 | 91 | 0.944918 | 0.004070 |  |  |  |
| 42 | 0.685156 | 0.006353 | 92 | 0.948988 | 0.003948 |  |  |  |
| 43 | 0.691509 | 0.006252 | 93 | 0.952937 | 0.003819 |  |  |  |
| 44 | 0.697761 | 0.006161 | 94 | 0.956756 | 0.003683 |  |  |  |
| 45 | 0.703923 | 0.006068 | 95 | 0.960439 | 0.003539 |  |  |  |
| 46 | 0.709990 | 0.005994 | 96 | 0.963979 | 0.003390 |  |  |  |
| 47 | 0.715984 | 0.005923 | 97 | 0.967369 | 0.003235 |  |  |  |
| 48 | 0.721907 | 0.005857 | 98 | 0.970604 | 0.003073 |  |  |  |
| 49 | 0.727764 | 0.005798 | 99 | 0.973676 | 0.002906 |  |  |  |

## Other broadleaves (*Fagacea spp*)

Regression analysis

Mean tree growth development based on age values from yield table of Sessile oak, (unique table) of experimental silviculture plot by J. P. Parde (1962) [18] at the Sector Ligérien and mean height and circumference values from five fitted non-linear regression data of the eight broadleaved trees (Chestnut, Hornbeam, Ash, Common Beech, Sessile oak, English oak and White oak) (Table 52).

Table 52: Mean growth based on eight broadleaved trees and fitted data

| Age | Top height | Circumference |
| --- | --- | --- |
| [yr] | [m] | [cm] |
| 30 | 14.89 | 44.87 |
| 36 | 17.07 | 52.77 |
| 42 | 18.97 | 60.27 |
| 48 | 20.62 | 67.37 |
| 54 | 22.05 | 74.08 |
| 60 | 23.29 | 80.40 |
| 66 | 24.35 | 86.37 |
| 72 | 25.27 | 91.99 |
| 78 | 26.06 | 97.28 |
| 84 | 26.73 | 102.28 |
| 90 | 27.32 | 106.99 |
| 98 | 27.98 | 112.87 |
| 106 | 28.51 | 118.32 |
| 114 | 28.96 | 123.37 |
| 122 | 29.32 | 128.08 |
| 130 | 29.62 | 132.46 |
| 140 | 29.92 | 137.52 |
| 150 | 30.16 | 142.17 |
| Source: adapted from [18] | | |

Model parameter results are illustrated in Table 53. The goodness of fit (GoF) for both age-height and age-circumference corresponds to 1. Results are plotted in Figure 54 and Figure 55, showing a comparison of the GoF from experimental yield table values and the fitted model.

Table 53: Characteristics and model fitting data of other broadleaved trees (*Fagacea spp*)

|  | A | k | p | RSS | GoF |
| --- | --- | --- | --- | --- | --- |
| Age-height growth | 30.91614 | 0.02558 | 1.16912 | 0.02313 | 1 |
| Age-circumference growth | 185.60 | 0.01 | 1.04 | 0.00999 | 1 |

| 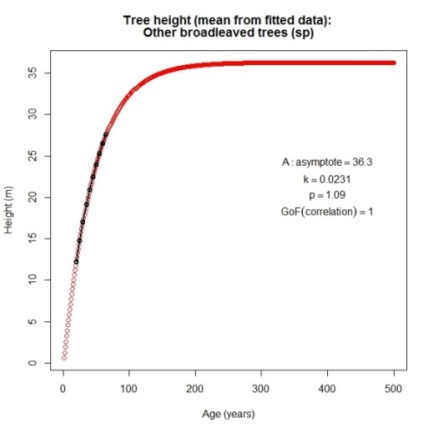 | 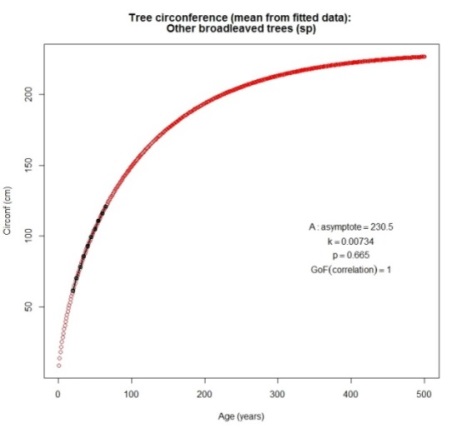 |
| --- | --- |
| Figure 54: Age-height mean growth curve and model parameter results of other broadleaved trees. Black dots represent yield table data, red circles model-predicted values | Figure 55: Age-circumference mean growth curve and model parameter results of other broadleaved trees. Black dots represent yield table data, red circles model-predicted values |

Biomass growth

Same as Sessile oak (*q. petraea*) with age-height growth. Aboveground volume estimations based on allometric equation and coefficients. Growth dynamics of individual stem volume (Figure 56) and managed stand (Figure 57), with 2.7 m^3^ and 532 m^3^·ha^-1^ respectively over 150-years rotation cycle.

| 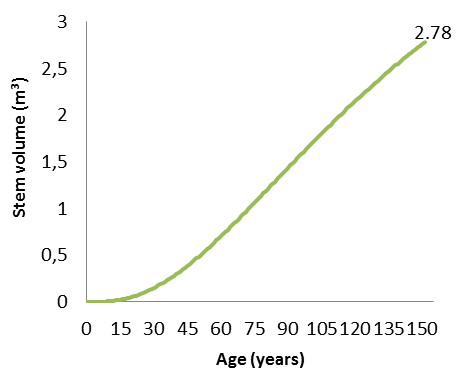 | 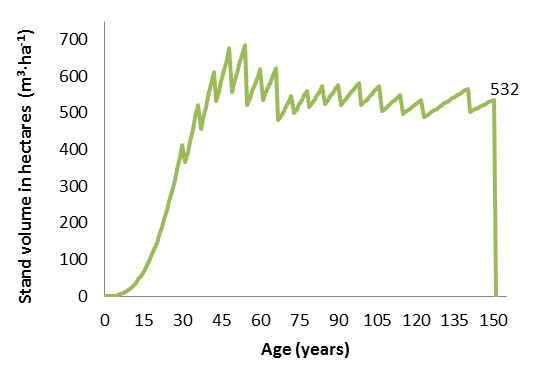 |
| --- | --- |
| Figure 56: Other broadleaved (*Fagacea spp*) mean aboveground individual stem volume (m^3^) over 150-year rotation cycle | Figure 57: Other broadleaved (*Fagacea spp*) mean aboveground volume per hectare (m^3^·ha^-1^) with thinning operations and clear cut at age 150 |

Carbon fixation

Carbon sequestration and storage of other broadleaved trees (*Fagacea spp*) over a 150-year rotation cycle (Table 54). Stocking factors applicable for rotation cycles before harvest (historic modelling approach).

Table 54: C_bio_ stocking factors for other broadleaved trees (*Fagacea spp*), 150-year rotation cycle

| Age  [yr] | C_bio_  growth  [t C_bio]_ | Annual C_bio_ stocking factors  [t C_bio_·yr^-1^] | Age  [yr] | C_bio_  growth  [t C_bio]_ | Annual C_bio_ stocking factors  [t C_bio_·yr^-1^] | Age  [yr] | C_bio_  growth  [t C_bio]_ | Annual C_bio_ stocking factors  [t C_bio_·yr^-1^] |
| --- | --- | --- | --- | --- | --- | --- | --- | --- |
| 0 | 0.000000 | 0.024965 | 60 | 0.791313 | 0.005306 | 120 | 0.984320 | 0.001286 |
| 1 | 0.024965 | 0.024594 | 61 | 0.796619 | 0.005214 | 121 | 0.985606 | 0.001225 |
| 2 | 0.049559 | 0.024212 | 62 | 0.801833 | 0.005123 | 122 | 0.986832 | 0.001165 |
| 3 | 0.073771 | 0.023820 | 63 | 0.806956 | 0.005035 | 123 | 0.987997 | 0.001105 |
| 4 | 0.097591 | 0.023418 | 64 | 0.811991 | 0.004948 | 124 | 0.989102 | 0.001046 |
| 5 | 0.121009 | 0.023005 | 65 | 0.816939 | 0.004864 | 125 | 0.990148 | 0.000986 |
| 6 | 0.144014 | 0.022583 | 66 | 0.821804 | 0.004784 | 126 | 0.991134 | 0.000927 |
| 7 | 0.166597 | 0.022152 | 67 | 0.826588 | 0.004705 | 127 | 0.992061 | 0.000868 |
| 8 | 0.188749 | 0.021712 | 68 | 0.831293 | 0.004627 | 128 | 0.992929 | 0.000810 |
| 9 | 0.210460 | 0.021263 | 69 | 0.835920 | 0.004550 | 129 | 0.993740 | 0.000754 |
| 10 | 0.231724 | 0.020807 | 70 | 0.840470 | 0.004475 | 130 | 0.994493 | 0.000698 |
| 11 | 0.252531 | 0.020344 | 71 | 0.844945 | 0.004401 | 131 | 0.995191 | 0.000643 |
| 12 | 0.272876 | 0.019875 | 72 | 0.849346 | 0.004330 | 132 | 0.995834 | 0.000589 |
| 13 | 0.292750 | 0.019400 | 73 | 0.853676 | 0.004260 | 133 | 0.996423 | 0.000536 |
| 14 | 0.312150 | 0.018920 | 74 | 0.857936 | 0.004190 | 134 | 0.996959 | 0.000484 |
| 15 | 0.331070 | 0.018436 | 75 | 0.862126 | 0.004121 | 135 | 0.997443 | 0.000434 |
| 16 | 0.349505 | 0.017949 | 76 | 0.866247 | 0.004052 | 136 | 0.997877 | 0.000385 |
| 17 | 0.367454 | 0.017460 | 77 | 0.870299 | 0.003985 | 137 | 0.998262 | 0.000339 |
| 18 | 0.384914 | 0.016970 | 78 | 0.874283 | 0.003918 | 138 | 0.998600 | 0.000294 |
| 19 | 0.401883 | 0.016480 | 79 | 0.878202 | 0.003853 | 139 | 0.998894 | 0.000252 |
| 20 | 0.418363 | 0.015992 | 80 | 0.882055 | 0.003787 | 140 | 0.999147 | 0.000213 |
| 21 | 0.434355 | 0.015507 | 81 | 0.885842 | 0.003722 | 141 | 0.999360 | 0.000176 |
| 22 | 0.449861 | 0.015026 | 82 | 0.889563 | 0.003656 | 142 | 0.999536 | 0.000142 |
| 23 | 0.464887 | 0.014551 | 83 | 0.893220 | 0.003592 | 143 | 0.999678 | 0.000111 |
| 24 | 0.479438 | 0.014084 | 84 | 0.896812 | 0.003529 | 144 | 0.999789 | 0.000083 |
| 25 | 0.493521 | 0.013626 | 85 | 0.900340 | 0.003465 | 145 | 0.999873 | 0.000058 |
| 26 | 0.507147 | 0.013179 | 86 | 0.903806 | 0.003402 | 146 | 0.999931 | 0.000038 |
| 27 | 0.520327 | 0.012746 | 87 | 0.907207 | 0.003338 | 147 | 0.999969 | 0.000021 |
| 28 | 0.533072 | 0.012328 | 88 | 0.910545 | 0.003275 | 148 | 0.999990 | 0.000009 |
| 29 | 0.545401 | 0.011929 | 89 | 0.913820 | 0.003212 | 149 | 0.999998 | 0.000002 |
| 30 | 0.557329 | 0.011550 | 90 | 0.917031 | 0.003149 | 150 | 1.000000 | 0.001286 |
| 31 | 0.568879 | 0.011183 | 91 | 0.920180 | 0.003087 |  |  |  |
| 32 | 0.580063 | 0.010826 | 92 | 0.923267 | 0.003024 |  |  |  |
| 33 | 0.590889 | 0.010481 | 93 | 0.926291 | 0.002960 |  |  |  |
| 34 | 0.601370 | 0.010148 | 94 | 0.929251 | 0.002897 |  |  |  |
| 35 | 0.611518 | 0.009831 | 95 | 0.932148 | 0.002834 |  |  |  |
| 36 | 0.621349 | 0.009531 | 96 | 0.934982 | 0.002771 |  |  |  |
| 37 | 0.630879 | 0.009241 | 97 | 0.937752 | 0.002708 |  |  |  |
| 38 | 0.640121 | 0.008960 | 98 | 0.940460 | 0.002646 |  |  |  |
| 39 | 0.649081 | 0.008690 | 99 | 0.943106 | 0.002584 |  |  |  |
| 40 | 0.657771 | 0.008430 | 100 | 0.945690 | 0.002521 |  |  |  |
| 41 | 0.666201 | 0.008184 | 101 | 0.948211 | 0.002459 |  |  |  |
| 42 | 0.674385 | 0.007953 | 102 | 0.950670 | 0.002396 |  |  |  |
| 43 | 0.682337 | 0.007731 | 103 | 0.953066 | 0.002333 |  |  |  |
| 44 | 0.690069 | 0.007518 | 104 | 0.955399 | 0.002270 |  |  |  |
| 45 | 0.697587 | 0.007314 | 105 | 0.957669 | 0.002208 |  |  |  |
| 46 | 0.704901 | 0.007121 | 106 | 0.959877 | 0.002147 |  |  |  |
| 47 | 0.712022 | 0.006940 | 107 | 0.962024 | 0.002085 |  |  |  |
| 48 | 0.718962 | 0.006772 | 108 | 0.964110 | 0.002023 |  |  |  |
| 49 | 0.725734 | 0.006613 | 109 | 0.966133 | 0.001961 |  |  |  |
| 50 | 0.732347 | 0.006460 | 110 | 0.968094 | 0.001899 |  |  |  |
| 51 | 0.738808 | 0.006315 | 111 | 0.969993 | 0.001837 |  |  |  |
| 52 | 0.745122 | 0.006177 | 112 | 0.971830 | 0.001775 |  |  |  |
| 53 | 0.751300 | 0.006049 | 113 | 0.973605 | 0.001714 |  |  |  |
| 54 | 0.757349 | 0.005932 | 114 | 0.975319 | 0.001653 |  |  |  |
| 55 | 0.763282 | 0.005820 | 115 | 0.976972 | 0.001592 |  |  |  |
| 56 | 0.769101 | 0.005710 | 116 | 0.978564 | 0.001531 |  |  |  |
| 57 | 0.774811 | 0.005603 | 117 | 0.980095 | 0.001470 |  |  |  |
| 58 | 0.780414 | 0.005499 | 118 | 0.981565 | 0.001408 |  |  |  |
| 59 | 0.785913 | 0.005400 | 119 | 0.982973 | 0.001347 |  |  |  |

# References

[1] H. Pretzsch, Forest Dynamics, Growth and Yield- From Measurement to Model, Springer-Verlag, Berlin Heidelberg, 2009. doi:10.1007/978-3-540-88307-4.

[2] L. Zhang, Cross-validation of non-linear growth functions for modelling tree height-diameter relationships, Ann. Bot. 79 (1997) 251–257. doi:10.1006/anbo.1996.0334.

[3] A. Pommerening, A. Muszta, Relative plant growth revisited: Towards a mathematical standardisation of separate approaches, Ecol. Modell. 320 (2016) 383–392. doi:10.1016/j.ecolmodel.2015.10.015.

[4] L. Zhao-gang, L. Feng-ri, The generalized Chapman-Richards function and applications to tree and stand growth, J. For. Res. 14 (2003) 19–26. doi:10.1007/BF02856757.

[5] Y. Yan, Integrate carbon dynamic models in analyzing carbon sequestration impact of forest biomass harvest, Sci. Total Environ. 615 (2018) 581–587. doi:10.1016/j.scitotenv.2017.09.326.

[6] J. Bouchon, J. Trencia, Sylviculture et production du chêne, Seichamps: INRA- Centre de recherches forestieères, 1990.

[7] D. Fekedulegn, M.P. Mac Siurtain, J.J. Colbert, Parameter estimation of nonlinear growth models in forestry, Silva Fenn. 33 (1999) 327–336. doi:10.14214/sf.653.

[8] L.V.. Pienaar, K.J. Turnbull, The Chapman-Richards generalization of Von Bertalanffy’s growth model for basal area growth and yield in even-aged stands, For. Sci. 19 (1973) 2–22. doi:https://doi.org/10.1093/forestscience/19.1.2.

[9] A. Pommerening, Arne Pommerening’s Webblog on Forest Biometrics, Swedish Univ. Agric. Sci. (2017). https://blogg.slu.se/forest-biometrics/2017/03/11/the-chapman-richards-growth-function/ (accessed November 15, 2017).

[10] R Core Team, R: A language and environment for statistical computing., R Found. Stat. Comput. (2018). http://www.r-project.org/.

[11] datascience+, First steps with Non-Linear Regression in R, Off. Website. (2018). https://datascienceplus.com/first-steps-with-non-linear-regression-in-r/ (accessed December 4, 2017).

[12] A.N. Spiess, N. Neumeyer, An evaluation of R2 as an inadequate measure for nonlinear models in pharmacological and biochemical research: A Monte Carlo approach, BMC Pharmacol. 10 (2010) 1–11. doi:10.1186/1471-2210-10-6.

[13] H. Oswald, Production et sylviculture du Douglas en plantations, Rev. For. Française. (1984) 268–278. http://documents.irevues.inist.fr/bitstream/handle/2042/21737/RFF_1984_4_268.pdf?sequence=1.

[14] S. González-García, V. Bonnesoeur, A. Pizzi, G. Feijoo, M.T. Moreira, The influence of forest management systems on the environmental impacts for Douglas-fir production in France, Sci. Total Environ. 461–462 (2013) 681–692. doi:10.1016/j.scitotenv.2013.05.069.

[15] France Douglas, Le Douglas: Resource/Matériau, Assoc. Fr. Douglas. (2017). https://www.france-douglas.com/le-douglas/ressource.html (accessed November 10, 2017).

[16] IGN, Le mémento inventaire forestier édition 2017, IGN-Institut Natl. l’information Géographique For. 2017 (2017) 30. https://inventaire-forestier.ign.fr/IMG/pdf/memento_2017.pdf (accessed July 20, 2018).

[17] IGN, L’IF: Prélèvement de bois en forêt et production biologique : des estimations directes et compatibles, Château des Barres, 2011. http://www.fnbois.com/sites/default/files/mediatheque/IFN_2011.pdf.

[18] INRA/ONF/ENGREF, Tables de production pour les forêts françaises, 2e édition, INRA-Centre National de Recherche Forestières, ONF- Office National des Forêts, EGREF- Ecole Nationale du Génie rural, des Eaix et des Forêts, Nancy, 1984.

[19] M. Menéndez-Miguélez, P. Álvarez-álvarez, J. Majada, E. Canga, Management tools for Castanea sativa coppice stands in northwestern Spain [Spanish: Herramientas de gestión para masas de monte bajo de Castanea sativa en el noroeste de España], Bosque. 37 (2016) 119–133. doi:10.4067/S0717-92002016000100012.

[20] A. Shvidenko, D. Schepaschenko, S. Nilsson, Y. Boului, Federal Agency of Forest Management International Institute for Applied Systems Analysis Tables and Models of Growth and Productivity of Forests of Major Forest Forming Species of Northern Eurasia (standard and reference materials), Moscow, 2008. http://webarchive.iiasa.ac.at/Research/FOR/forest_cdrom/Articles/THR.pdf.
